# Expert consensus statements for the management of COVID-19-related acute respiratory failure using a Delphi method

**DOI:** 10.1186/s13054-021-03491-y

**Published:** 2021-03-16

**Authors:** Prashant Nasa, Elie Azoulay, Ashish K. Khanna, Ravi Jain, Sachin Gupta, Yash Javeri, Deven Juneja, Pradeep Rangappa, Krishnaswamy Sundararajan, Waleed Alhazzani, Massimo Antonelli, Yaseen M. Arabi, Jan Bakker, Laurent J. Brochard, Adam M. Deane, Bin Du, Sharon Einav, Andrés Esteban, Ognjen Gajic, Samuel M. Galvagno, Claude Guérin, Samir Jaber, Gopi C. Khilnani, Younsuck Koh, Jean-Baptiste Lascarrou, Flavia R. Machado, Manu L. N. G. Malbrain, Jordi Mancebo, Michael T. McCurdy, Brendan A. McGrath, Sangeeta Mehta, Armand Mekontso-Dessap, Mervyn Mer, Michael Nurok, Pauline K. Park, Paolo Pelosi, John V. Peter, Jason Phua, David V. Pilcher, Lise Piquilloud, Peter Schellongowski, Marcus J. Schultz, Manu Shankar-Hari, Suveer Singh, Massimiliano Sorbello, Ravindranath Tiruvoipati, Andrew A. Udy, Tobias Welte, Sheila N. Myatra

**Affiliations:** 1 Critical Care Medicine, NMC Speciality Hospital, Dubai, United Arab Emirates; 2 Saint-Louis teaching hospital – APHP – and University of Paris, Paris, France; 3 Wake Forest University School of Medicine, Winston-Salem, NC and Outcomes Research Consortium , Cleveland, USA; 4 Mahatma Gandhi Medical College and Hospital, Jaipur, India; 5 Narayana Super Speciality Hospital, Gurugram, India; 6 Regency Super Speciality Hospital, Lucknow, India; 7 Max Super Speciality Hospital, New Delhi, India; 8 Columbia Asia Referral Hospital, Bengaluru, India; 9 Royal Adelaide Hospital and The University of Adelaide, Adelaide, Australia; 10 McMaster University, Hamilton, Canada; 11 Fondazione Policlinico Universitario A. Gemelli IRCCS, Rome, Italy; 12 King Saud Bin Abdulaziz University for Health Sciences and King Abdullah International Medical Research Centre, Riyadh, Saudi Arabia; 13 New York University School of Medicine and Columbia University College of Physicians & Surgeons, New York, USA; 14 Erasmus MC University Medical Center, Rotterdam, The Netherlands; 15 Pontificia Universidad Catolica de Chile, Santiago, Chile; 16 Keenan Research Centre, Li Ka Shing Knowledge Institute, St Michael’s Hospital, Unity Health Toronto, and University of Toronto, Toronto, Canada; 17 Royal Melbourne Hospital and The University of Melbourne, Melbourne, Australia; 18 Peking Union Medical College Hospital, Peking, China; 19 The Shaare Zedek Medical Center, Jerusalem, Israel; 20 Hospital Universitario de Getafe, CIBER de Enfermedades Respiratorias, Madrid, Spain; 21 Mayo Clinic, Maryland, USA; 22 University of Maryland, Maryland, USA; 23 University de Lyon, Lyon, France; 24 Institut Mondor de Recherches Biomédicales, Medecine Intensive Réanimation Hôpital Edouard Herriot Lyon, and Medecine Intensive Réanimation Hôpital Edouard Herriot Lyon, Créteil, France; 25 Montpellier University Hospital, Montpellier, France; 26 Hôpital Saint-Éloi, CHU de Montpellier, Phy Med Exp, Université de Montpellier, Montpellier, France; 27 PSRI Hospital, New Delhi, India; 28 Asan Medical Center, University of Ulsan College of Medicine, Seoul, South Korea; 29 Nantes University Hospital, Nantes, France; 30 Federal University of São Paulo, São Paulo, Brazil; 31 International Fluid Academy, Lovenjoel, Belgium; 32 Faculty of Engineering, Department of Electronics and Informatics, Vrije Universiteit Brussel (VUB), Brussels, Belgium; 33 Hospital Universitari Sant Pau, Barcelona, Spain; 34 University of Maryland School of Medicine, Maryland, USA; 35 Manchester University NHS Foundation Trust, Manchester, UK; 36 Division of Infection, Immunity and Respiratory Medicine, School of Biological Sciences, Faculty of Biology Medicine and Health, University of Manchester, Academic Health Sciences Centre, Manchester, UK; 37 Sinai Health and the University of Toronto, Toronto, Canada; 38 Assistance Publique - Hôpitaux de Paris, Hôpitaux Universitaires Henri-Mondor, Service de Medicine Intensive Réanimation, and Univ Paris Est Créteil, CARMAS, Créteil, France; 39 Charlotte Maxeke Johannesburg Academic Hospital and Faculty of Health Sciences, University of the Witwatersrand, Johannesburg, South Africa; 40 Cedars-Sinai Medical Center, Smidt Heart Institute, Los Angeles, USA; 41 University of Michigan, Ann Arbor, USA; 42 San Martino Policlinico Hospital, IRCCS for Oncology and Neurosciences , Genoa, Italy; 43 Department of Surgical Sciences and Integrated Sciences, University of Genoa , Genoa, Italy; 44 Christian Medical College, Vellore, India; 45 Alexandra Hospital and National University Hospital, Singapore, Singapore; 46 Alfred Health, and Monash University, Melbourne, Australia; 47 Lausanne University Hospital and Lausanne University, Lausanne, Switzerland; 48 Medical University of Vienna, Vienna, Austria; 49 Amsterdam University Medical Center, Amsterdam, The Netherlands; 50 Mahidol University, Bangkok, Thailand; 51 University of Oxford, Oxford, UK; 52 Guy’s and St Thomas’ NHS Foundation Trust, London, UK; 53 King’s College London, London, UK; 54 Royal Brompton Hospital and Chelsea and Westminster Hospital, Imperial College, London, UK; 55 Anesthesia and Intensive Care , AOU Policlinico - San Marco, Catania, Italy; 56 Peninsula Health and Monash University, Melbourne, Australia; 57 Monash University, Melbourne, Australia; 58 Department of Respiratory Medicine, German Centre of Lung Research, Hannover, Germany; 59 Department of Anaesthesia, Critical Care and Pain, Tata Memorial Hospital, Homi Bhabha National Institute, Dr. Ernest Borges Road, Parel, Mumbai India

**Keywords:** Respiratory distress syndrome adult, COVID-19 ventilatory management, COVID-19 respiratory management, COVID-19 acute respiratory distress syndrome, COVID-19 high flow nasal oxygen, COVID 19 invasive mechanical ventilation

## Abstract

**Background:**

Coronavirus disease 2019 (COVID-19) pandemic has caused unprecedented pressure on healthcare system globally. Lack of high-quality evidence on the respiratory management of COVID-19-related acute respiratory failure (C-ARF) has resulted in wide variation in clinical practice.

**Methods:**

Using a Delphi process, an international panel of 39 experts developed clinical practice statements on the respiratory management of C-ARF in areas where evidence is absent or limited. Agreement was defined as achieved when > 70% experts voted for a given option on the Likert scale statement or > 80% voted for a particular option in multiple-choice questions. Stability was assessed between the two concluding rounds for each statement, using the non-parametric Chi-square (*χ*^2^) test (*p* < 0·05 was considered as unstable).

**Results:**

Agreement was achieved for 27 (73%) management strategies which were then used to develop expert clinical practice statements. Experts agreed that COVID-19-related acute respiratory distress syndrome (ARDS) is clinically similar to other forms of ARDS. The Delphi process yielded strong suggestions for use of systemic corticosteroids for critical COVID-19; awake self-proning to improve oxygenation and high flow nasal oxygen to potentially reduce tracheal intubation; non-invasive ventilation for patients with mixed hypoxemic-hypercapnic respiratory failure; tracheal intubation for poor mentation, hemodynamic instability or severe hypoxemia; closed suction systems; lung protective ventilation; prone ventilation (for 16–24 h per day) to improve oxygenation; neuromuscular blocking agents for patient-ventilator dyssynchrony; avoiding delay in extubation for the risk of reintubation; and similar timing of tracheostomy as in non-COVID-19 patients. There was no agreement on positive end expiratory pressure titration or the choice of personal protective equipment.

**Conclusion:**

Using a Delphi method, an agreement among experts was reached for 27 statements from which 20 expert clinical practice statements were derived on the respiratory management of C-ARF, addressing important decisions for patient management in areas where evidence is either absent or limited.

*Trial registration*: The study was registered with Clinical trials.gov Identifier: NCT04534569.

**Supplementary Information:**

The online version contains supplementary material available at 10.1186/s13054-021-03491-y.

## Introduction

Infection with the severe acute respiratory syndrome coronavirus 2 (SARS-CoV-2) has emerged as a pandemic, resulting in unprecedented pressure on healthcare systems globally. Although most patients present with mild symptoms including fever and malaise, 8–32% of patients presenting to hospital may require admission to the intensive care unit (ICU) [1–3], depending on the admission criteria and available resources, with an ICU mortality of 34–50% [3, 4].

Patients with coronavirus disease 2019 (COVID-19) acute respiratory failure (C-ARF) who are admitted to the ICU with hypoxaemia typically require some form of respiratory support [5]. COVID-19-related acute respiratory distress syndrome (ARDS) may differ from other causes of ARDS, since patients may present with profound hypoxaemia accompanied by a wide range of respiratory compliance [6–8]. However, whether ARDS due to COVID-19 is clinically similar to other forms of ARDS remains a matter of debate [7, 9–11]. Consequently, there is no uniform agreement on the optimal management of respiratory failure, including the most appropriate oxygenation and ventilation strategies that limit or prevent additional lung injury or other complications in these patients.

There are few published randomised controlled trials (RCTs) related to the respiratory management of C-ARF. As a result, clinical practice variations in the management of C-ARF exist, making the optimal therapeutic management unclear [12]. Given the dearth of evidence, we aimed to achieve agreement on the respiratory management of C-ARF using a Delphi process, defined by at least 70% agreement among experts who met pre-specified qualification criteria.

## Methods

### Delphi process

A steering committee of 10 critical care physicians actively involved in the management of patients with C-ARF was formed in August 2020. A Delphi process was used to generate agreement on the respiratory management of C-ARF [13, 14]. The study was registered with Clinical trials.gov Identifier: NCT04534569.

The steering committee recruited and convened an international group of intensivists with expertise in the field of acute respiratory failure. E-mail invitations were sent to 60 global experts to participate in the Delphi process. Upon acceptance, experts were included in the Delphi process to generate agreement. Surveys disseminated to the experts were prepared using Google Forms. The steering committee members did not participate in the Delphi process.

The overall scope of the project was determined through a search and review of available literature on C-ARF, published between 1^st^ January and 3^rd^ September 2020 by the steering committee (Additional file 1). A list of interventions for the respiratory management of C-ARF was prepared in areas where the committee felt clear evidence was lacking. The list was presented to experts in the form of a survey questionnaire, which included five sections: non-invasive respiratory interventions; invasive mechanical ventilation; refractory hypoxaemia; infection control; weaning and tracheostomy. The experts subsequently responded to several rounds of survey questionnaires conducted using an iterative approach using the Delphi method, to prioritise topics for inclusion, which were repeated until agreement and stability were achieved. Complete details of the Delphi process are given in Additional file 2.

### Agreement and stability

For statements with responses on an ordinal 7–point Likert scale, ‘agreement’ was defined as a score of 5–7, ‘neutral’ by a score of 4 and ‘disagreement’ by a score of 1–3. Agreement was defined as achieved when > 70% experts voted for a given option on the Likert scale for a statement [13, 14]. Median and interquartile range (IQR) were used to describe the central tendency and dispersion of responses. For multiple-choice questions (MCQs), agreement was defined as achieved if > 80% experts voted for a particular option. Stability in the responses was assessed from round three onwards. Stability was assessed between the two concluding rounds for each statement, using the non-parametric Chi-square (*χ*^2^) test. *p* < 0·05 was considered as a significant variation or unstable. Data from the last stable questionnaire round of the Delphi process for each statement were included for preparing the final clinical statements.

### Expert clinical practice statements

Expert clinical practice statements were derived by the steering committee from the clinical statements that generated agreements through the Delphi process. The expert clinical practice statements were considered to be “strong statements” when a median of ≥ 6 or ≤ 2 on the Likert scale or > 90% votes for any MCQ option were achieved [14]. For the expert clinical practice statements, the term “should” was used for the strong statements and “may” was used for the other statements.

The final results of this survey and the expert clinical practice statements were circulated among the experts. The manuscript was circulated among the experts for editing and approval before it was submitted for publication.

## Results

**Fig. 1:**
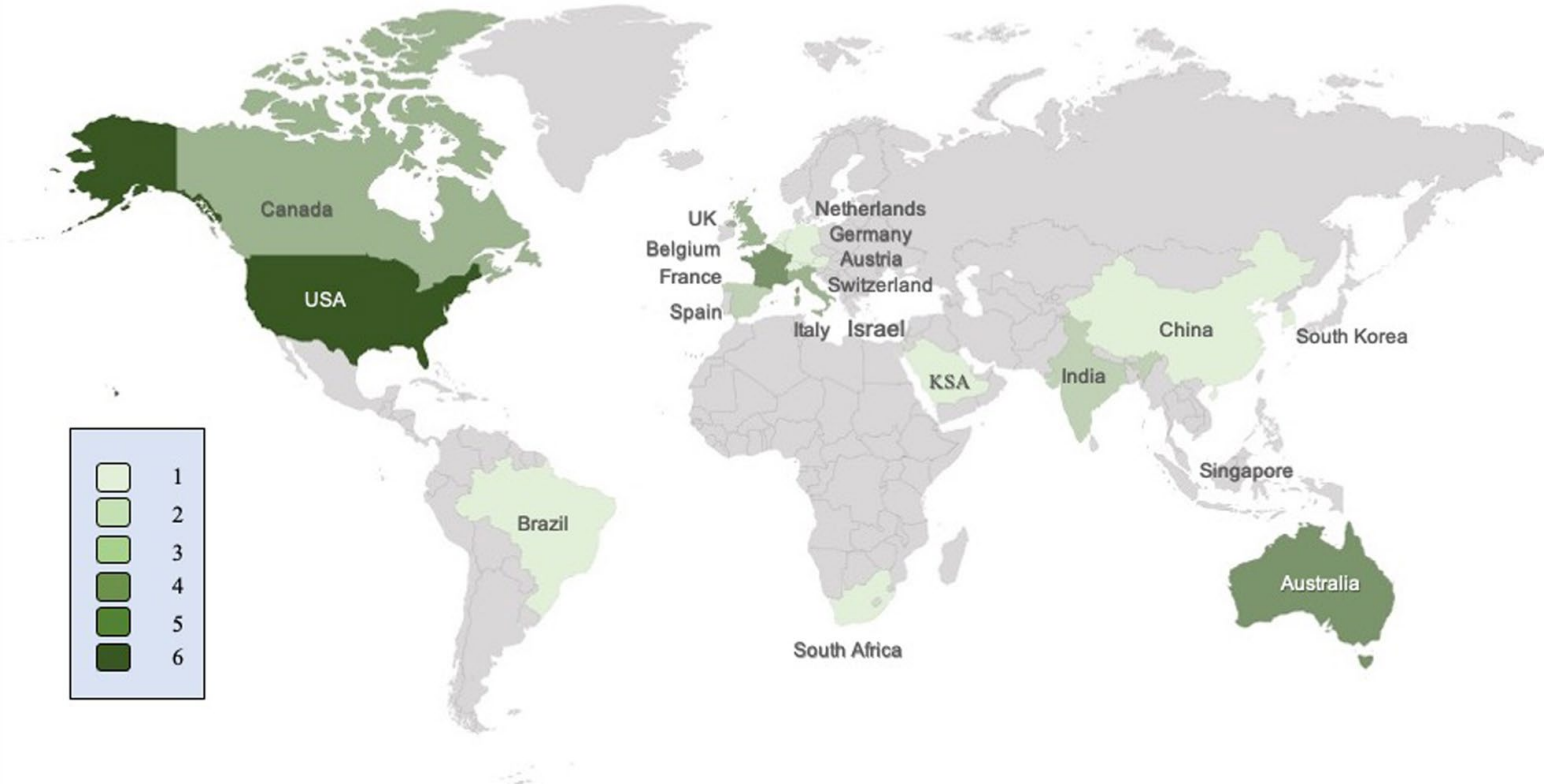
Geographical distribution of countries represented by the experts. KSA: Kingdom of Saudi Arabia; UK: United Kingdom; USA: United States of America. Different shades of green represent the number of experts

Of the 60 experts invited, 39 (65%) from 20 different countries and six continents participated in the Delphi process (Fig. 1); and 37 (95%) completed all rounds of the Delphi process. Median age of the experts was 53 (13) years and 5 (13%) were female. The majority (92%) were affiliated with university hospitals; and their median h-index was 33 (11–100).

**Fig. 2:**
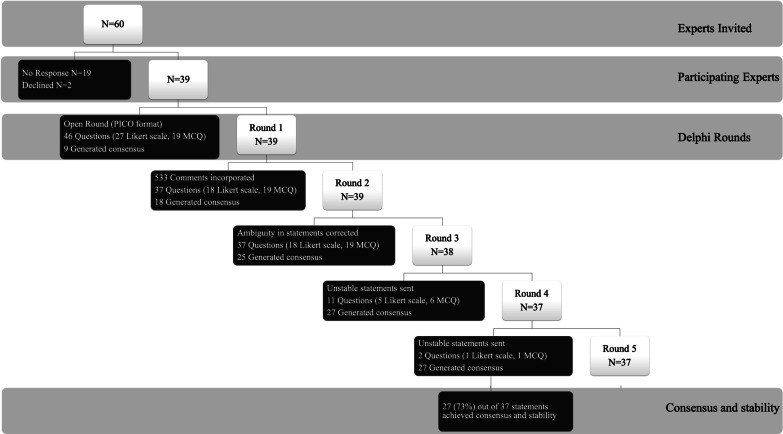
Flow diagram of the steps of the Delphi process. N: number of experts; MCQ: multiple choice question; PICO: Patient problem, Intervention: Comparison and Outcome

**Fig. 3:**
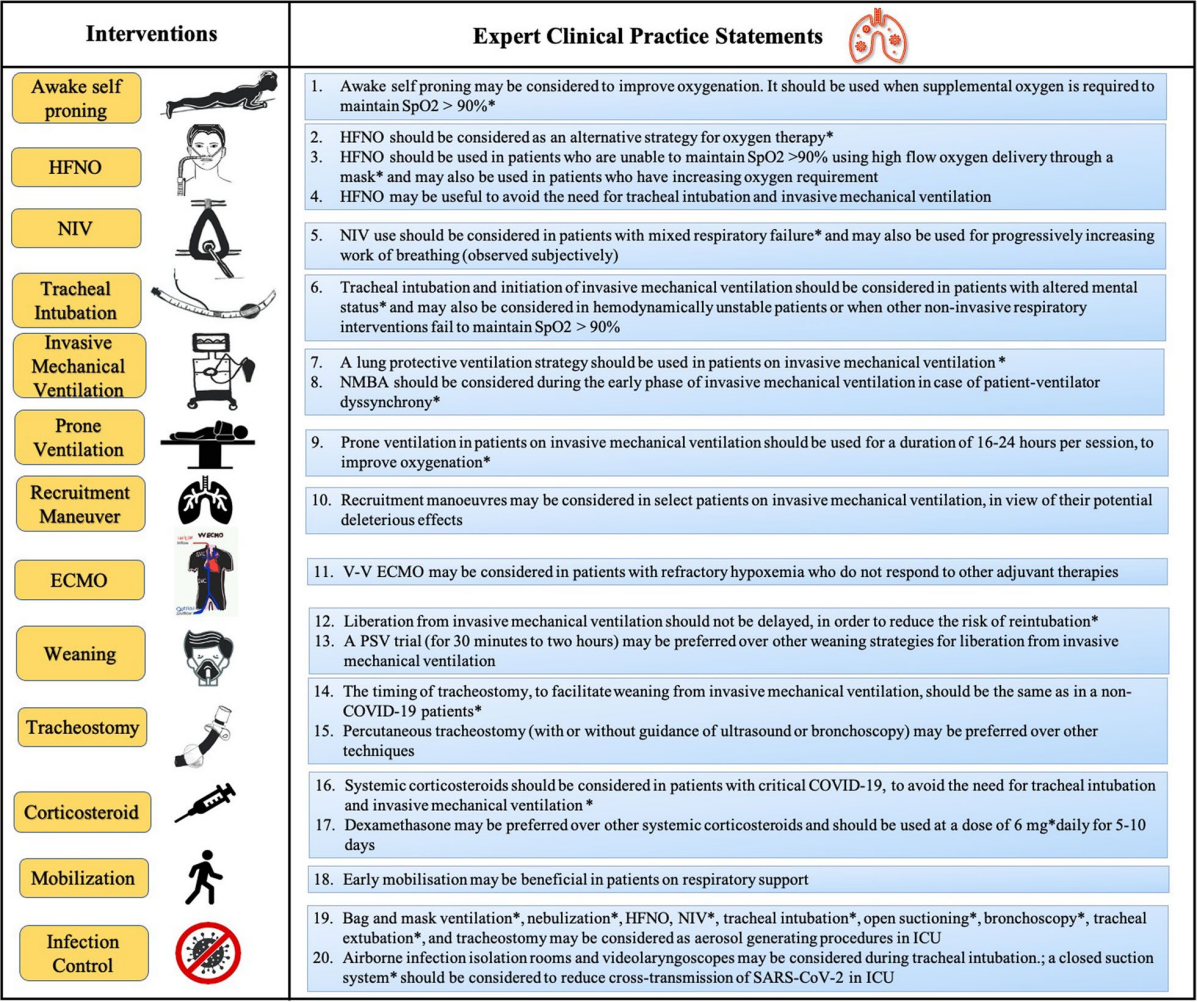
Expert clinical practice statements for the respiratory management of COVID-19-related acute respiratory failure. *Strong statement (a median of ≥ 6 or ≤ 2 on the Likert scale or > 90% votes for any MCQ option were achieved). HFNO: high flow nasal oxygen; NIV: non-invasive ventilation; NMBA: neuromuscular blocking agent; PEEP: positive end-expiratory pressure; VV-ECMO: veno-venous extracorporeal membrane oxygenation; PSV: pressure support ventilation; COVID-19: coronavirus disease 2019; ICU: intensive care unit; SARS-CoV-2: severe acute respiratory syndrome coronavirus 2; C-ARF: COVID-19-related acute respiratory failure

Five survey questionnaire rounds were conducted between 4th September and 5th October 2020. Details of the Delphi rounds are provided in Fig. 2. The results of all 37 survey questionnaire statements used in the Delphi process are given in Table 1. At the end of the Delphi process, 27 statements (73%) achieved agreement and stability from which 20 expert clinical practice statements were prepared (Fig. 3). Reports of the first four survey rounds are provided in the online supplement (Additional file 3: Survey Report 1, Additional file 4: Survey Report 2, Additional file 5: Survey Report 3, and Additional file 6: Survey Report 4).

**Table 1:** Consensus and stability analysis of the clinical statements on the respiratory management of C-ARF

	Agree. (%)	Neutral (%)	Disagree. (%)	Median (IQR)	*χ*^2^ p-value
**Section-1: Non-invasive respiratory interventions**					
1. The pathophysiology of C-ARF is similar to that of ARDS	86.5	0	13.5	5 (0)	0.05
2. Based on your experience, awake self-proning may improve oxygenation in patients with C-ARF	91.9	8.1	0	5 (1)	1.0
3. Based on your experience, awake self-proning may prevent the need for invasive mechanical ventilation in patients with C-ARF*	54.0	35.1	10.9	5 (1)	0.71
4. In which of the following clinical scenarios should awake self-proning be initiated in patients with C-ARF?				NA	0.21
Supplemental oxygen required to maintain SpO_2_ > 90%	97.8				
Moderate-to-severe COVID-19	73				
Increased work of breathing (observed subjectively)	45.9				
Tachypnea (respiratory rate ≥ 30/min)	37.8				
Never	0				
5. HFNO can be considered as an alternative strategy for oxygen support before invasive mechanical ventilation	97.3	2.7	0	6 (0)	0.09
6. When do you initiate HFNO in patients with C-ARF?				NA	0.28
Unable to maintain SpO_2_ > 90% using high flow oxygen delivery device through a mask	97.3				
Increasing oxygen requirement	81.1				
Moderate-to-severe COVID-19	73				
Tachypnea (respiratory rate ≥ 30/min)	56.8				
Increased work of breathing (observed subjectively)	54.1				
Never	0				
7. Based on your experience, HFNO may avoid the need for tracheal intubation and invasive mechanical ventilation in patients with C-ARF	81.1	18.9	0	5 (0)	0.35
8. NIV can be considered as an alternative strategy for oxygen support before invasive mechanical ventilation*	64.8	18·9	16.3	5 (2)	0.88
9. NIV may be considered in the following clinical scenarios in patients with C-ARF?				NA	0.44
Mixed Respiratory failure (hypercapnia and hypoxemia)	94.6				
Increased work of breathing (observed subjectively)	81.1				
Unable to maintain SpO_2_ more than 90% with high flow oxygen delivery through a mask	67.6				
Moderate-to-severe COVID-19	59.5				
Tachypnea (respiratory rate ≥ 30/min)	51.4				
Unable to maintain Spo2 more than 90% with HFNO	45.9				
10. Based on your experience, NIV may avoid the need for tracheal intubation and invasive mechanical ventilation in patients with C-ARF*	64.8	21.6	13.5	5 (1)	0.06
11. The use of systemic corticosteroids could potentially avoid the need for tracheal intubation and invasive mechanical ventilation in C-ARF	86.5	10.8			0.27
12. In which clinical context would you choose to initiate corticosteroids in C-ARF?				NA	0.35
Critical COVID-19	91.9				
Oxygen requirement to maintain SpO_2_ more than 92%	73				
Moderate-to-severe COVID-19	75.7				
All patients with C-ARF	37.8				
Taking into consideration of inflammatory markers (CRP etc.)	24.3				
Never	0				
13. Which corticosteroid is your preferred choice in patients with C-ARF?				NA	0.30
Dexamethasone	86.5				
Methylprednisolone	16.2				
Type of steroid is immaterial	16.2				
Hydrocortisone	5.4				
14. What daily dose of corticosteroid (equivalent dose of dexamethasone) you prescribe for C-ARF?				NA	0.22
6 mg (equal to 8 mg of dexamethasone phosphate)	91.9				
7 mg–20 mg	10.8				
> 20 mg	2.7				
Other	0				
15. What duration of corticosteroid use would you prefer for patients with C-ARF?				NA	0.81
5–10 days	86.5				
Extended duration for more than 10 days depending on the clinical response	13.5				
11–14 days	2.7				
More than 14 days	2.7				
**Section-2: Invasive mechanical ventilation**					
1. Which of the following options may be considered as an appropriate trigger for tracheal intubation in C-ARF?				NA	0.05
Altered mental status	91.9				
Hemodynamic instability	81.1				
Failure to maintain SpO_2_ > 90% with other non-invasive respiratory interventions	81.1				
Persistent respiratory distress	78.4				
PaO_2_/FiO_2_ less than 100	67.6				
Increased work of breathing (observed subjectively)	62.2				
PaO2/FiO2 less than 200	18.9				
Tachypnea (respiratory rate ≥ 30/min)	3.8				
2. “Lung protective ventilation” should be used for patients with C-ARF on IMV	100	0	0	6 (1)	1.0
3. A low PEEP strategy (≤ 10 cm of H2O) is usually considered during IMV of C-ARF*	29.7	51.4	18.9	4 (1)	0.003
4. How would you select PEEP in a patient of C-ARF on invasive mechanical ventilation with thorax CT scan showing bilateral pulmonary infiltrates, PaO_2_/FiO_2_ ratio less than 100 mm Hg, plateau pressure 27 cm of H_2_O and PEEP of 6 cm of H_2_O?*				NA	NA
Obtaining the best static compliance or lowest driving pressure	54.1				
Recruitment manoeuvre followed by PEEP set to either optimal SpO_2_ or static lung compliance	40.5				
Incremental PEEP to target plateau pressure less than 30 cm H_2_O	40.5				
Using ARDS-net protocol PEEP tables	37.8				
Based on pressure–volume curve	29.7				
Using esophageal balloon	16.2				
Other	8.1				
5. NMBA may be considered during early phase of the invasive mechanical ventilation of C-ARF to avoid patient-ventilator dyssynchrony	89.1	8.2	2.7	6 (1)	0·74
6. The invasive mechanical ventilation strategy in C-ARF should be targeted to the following?				NA	0.94
Tidal volume 4–6 ml/kg of predicted body weight	89.2				
Plateau pressure ≤ 30 cm of H_2_O	89.2				
Driving pressure ≤ 15 cm of H_2_O	78.4				
Oxygenation (PaO_2_/FiO_2_ ratio)	29.4				
Tidal volume 7–8 ml/kg of predicted body weight	10.8				
Other	0				
**Section-3: Refractory hypoxemia**					
1. The use of RM in patients with refractory hypoxemia in the setting of C-ARF needs to be personalized to the individual patient in view of its potential deleterious effects	89.2	5.4	5.4	5 (1)	0.26
2. Prone position during invasive mechanical ventilation of C-ARF improves oxygenation	97.3	2.7	0	6 (1)	0.09
3. Prone position during invasive mechanical ventilation of C-ARF is effective when done for (duration per session)?				NA	0.25
16–24 h	94.6				
12–15 h	16.2				
> 24 h	5.4				
12–16 h	0				
4. Advanced invasive mechanical ventilation (APRV, PRVC, etc.) modes may be beneficial in refractory hypoxemia with C-ARF*	16.3	43.2	40.5	4(2)	0.03
5. The following adjuvant therapies are effective in refractory hypoxemia with C-ARF?*				NA	0.1
None	54.1				
Inhaled nitric oxide	45.9				
Other	5.4				
Nebulized prostacyclin	8.1				
6. V-V ECMO may be considered in C-ARF patients on invasive mechanical ventilation?				NA	0.48
Only in patients with refractory hypoxemia, who do not respond to other adjuvant therapies	83.8				
Depending on the national/institutional policy and judicious resource allocation decision	62.2				
Only in patients who have failed or have a contraindication to prone positioning	45.9				
Early in patients with C-ARF without a trial of prone positioning	2.7				
Cannot comment	0				
Never	0				
**Section-4: Infection control**					
1. The following are considered as aerosol-generating procedures (AGPs)?				NA	0.54
Tracheal intubation	100				
Tracheostomy	100				
Bronchoscopy	100				
Tracheal extubation	97.3				
Bag and mask ventilation	97.3				
Non-invasive ventilation	97.3				
Open suctioning (oral or tracheal)	97.3				
Nebulization	94.6				
High-flow nasal oxygen therapy	81.1				
Chest physiotherapy	64.9				
Invasive mechanical ventilation	10				
2. HFNO produces less aerosols as compared to NIV with face mask*	37.8	54.1	8.1	4 (1)	0.002
3. The following measures may be considered in the ICU to prevent cross-transmission of SARS-CoV-2?				NA	0.66
Closed suction system	100				
Airborne infection isolation room	89.2				
Video laryngoscopy over conventional laryngoscopy for intubation	86.5				
Heat and moisture exchange filters	62.2				
Ventilatory circuit modification for NIV /invasive mechanical ventilation	54.1				
Increasing outdoor air ventilation rates (opening windows of ICU)	51.4				
NIV with helmet	48.6				
Subglottic secretion drainage endotracheal tube	32.4				
Intubation boxes	35.1				
Delaying tracheal extubation up to ten days	2.7				
Which personal protective equipment is acceptable for use during an AGP in ICU?*				NA	0.08
Coverall, goggles or face shield, surgical gloves and N95 (FFP 2) mask	64.9				
Coverall, surgical gloves, N95 (FFP 2) mask, goggles and face shield	59.5				
Coverall, goggles or face shield, surgical gloves and FFP 3 mask	45.9				
Coverall, surgical gloves and powered air-purifying respirator (PAPR)	40.5				
PAPR and surgical gloves	8.1				
Coverall, goggles or face shield, surgical gloves and surgical mask	2.7				
N95 and surgical gloves	0				
**Section-5: Weaning and tracheostomy**					
1. Which weaning strategy would you prefer for liberation from invasive mechanical ventilation in patients with C-ARF?				NA	0.33
Pressure support ventilation trial for 30 min to 2 h	89.2				
Protocolized weaning	27				
T-piece trial for 30 min to 2 h	13.5				
Automated weaning protocol on mechanical ventilation	8.1				
2. Chest physiotherapy could be beneficial in patients with C-ARF*	62.2	32.4	5.4	5 (1)	0.20
3. Early mobilization of patients is beneficial in patients on respiratory support for C-ARF	94.6	5.4	0	5 (1)	0.16
4. Delay in liberation from invasive mechanical ventilation has lower risk of reintubation in patients with C-ARF	2.7	24.3	73	2 (2)	0.38
5. When should tracheostomy be considered to facilitate weaning from invasive mechanical ventilation?				NA	0.80
Same timing as in a non-COVID-19 patient	91.9				
Failed tracheal extubation	13.5				
Later than you would perform in a non-COVID-19 patient	10.8				
Earlier than you would perform in a non-COVID-19 patient	0				
Which of the following technique of performing tracheostomy is preferred in patients with C-ARF?				NA	0.42
Percutaneous tracheostomy with or without guidance (ultrasound or bronchoscopic)	89.2				
Surgical tracheostomy in the operation theatre	24.9				
Surgical tracheostomy at the bed side	16.2				
Other	2.7				

Likert scale responses are presented as a percentage of agreement, neutral and disagreement

Options of the multiple-choice type statements are presented in descending order of consensus

Consensus was achieved when there was > 70% agreement/disagreement for the Likert scale and > 80% for multiple-choice type statements

*Clinical statements that did not reach consensus

Median and interquartile range (IQR) were used to describe the central tendency of responses and dispersion along the central value

*p* value was calculated using *χ*^2^: Chi-square**.** *p* value was a measure of stability in responses between the two concluding rounds for each statement. *p* < 0·05 was considered as a significant variation or unstable

Critical COVID-19 is used for ARDS (as per the Berlin definition), sepsis and septic shock (WHO severity definition for COVID-19)^24^

Agree: agreement; Disagree: disagreement; IQR: interquartile range; C-ARF: COVID-19-related acute respiratory failure; ARDS: acute respiratory distress syndrome; HFNO: high-flow nasal oxygen; NIV: non-invasive ventilation; COVID-19: coronavirus disease 2019; PEEP: positive end expiratory pressure; CT: computed tomography; NMBA: neuromuscular blocking agent; APRV: airway pressure release ventilation; PRVC: pressure-regulated volume control; V–V ECMO: veno–venous extracorporeal membrane oxygenation; AGP: aerosol-generating procedure; ICU: intensive care unit; SARS-CoV-2: severe acute respiratory syndrome coronavirus 2; FFP: filtering face piece; PAPR: powered air-purifying respirator.

## Expert clinical practice statements

Our study rapidly conducted a survey of recognised international experts using the Delphi process, generating 27 statements with large agreement on the respiratory management of C-ARF. From these statements, 20 expert clinical practice statements were derived, addressing critical knowledge gaps in clinical management. The experts made a number of important and relevant recommendations specific to C-ARF covering invasive and non-invasive respiratory support, pharmacology, airway management, infrastructure and recovery.

The Delphi methodology is a well-recognized process to generate guidance based on consensus using collective intelligence [13, 14]. The expert clinical practice statements address important bedside decisions for patient management in areas where the current evidence is either absent or limited. These expert statements along with a discussion of the available literature are detailed below.

### Is COVID-19-related ARDS similar to other forms of ARDS?

#### Expert statement

COVID-19-related ARDS is clinically similar to other forms of ARDS.

#### Discussion

The pathophysiology of COVID-19 involves SARS-CoV-2 invasion of host cells using angiotensin converting enzyme 2 (ACE2) receptor present in lungs and other organs. The viral invasion is followed by replication in type II alveolar pneumocytes that induces a dysregulated host immune response which in turn causes alveolar damage and ARDS [15]. Lung autopsy studies in patients who died from COVID-19 demonstrate diffuse alveolar damage along with significant endotheliitis and microthrombi in the pulmonary microvasculature [16–18]. Diffuse alveolar damage and alveolar haemorrhage with capillary damage are also noted in patients with non-COVID-19-related ARDS [18, 19]. In a cohort of 31 patients with COVID-19, higher lung compliance and volumes were found compared to patients with non-COVID-19 ARDS for a given PaO2/FiO2 [6, 8, 20]. This created the controversy that the pathophysiology of COVID-19-related ARDS is different from conventional ARDS [6, 7, 9–11]. Though there may be some differences in the pathophysiology of COVID-19 and non-COVID-19 ARDS, the clinical presentation is similar [16–18]. The respiratory mechanics of ventilated patients with C-ARF were noted to be similar to classical ARDS in larger observational multicentre studies [21–23]. Further studies incorporating lung imaging and perfusion analysis will better address this important pathophysiological and clinical issue in future.

### Corticosteroids

#### Expert statement

Systemic corticosteroids should be used in patients with critical COVID-19.

Dexamethasone is the preferred choice of systemic corticosteroids in patients of C-ARF.

The daily dose of dexamethasone should be 6 mg.

The preferred duration of systemic corticosteroids is 5–10 days.

#### Discussion

There is a strong suggestion for the use of systemic corticosteroids in critical COVID-19 [World Health Organisation (WHO) definition for COVID-19-ARDS, sepsis and septic shock] to reduce the need for invasive mechanical ventilation [24]. Experts preferred the use of dexamethasone at a dose of 6 mg daily for a duration of 5–10 days, as used in the RECOVERY trial over other corticosteroids, higher dose and longer duration [25]. The RECOVERY trial and subsequent trials on corticosteroids in COVID-19 found a mortality benefit with its use [25–27]. However, some questions remain unanswered, such as the type, duration of corticosteroid therapy, timing of initiation and role of a higher dose [28]. The results of ongoing trials (ClinicalTrials.gov Identifier NCT04395105 and NCT04509973) will provide further information.

### Awake self-proning

#### Expert statement

Awake self-proning may improve oxygenation when used in patients with C-ARF requiring supplemental oxygen to maintain oxygen saturation (SpO_2_) > 90%.

#### Discussion

Use of early prone position in patients with severe ARDS on invasive mechanical ventilation has been shown to significantly reduce mortality [28]. Though small observational studies in non-COVID-19 [29] and COVID-19 [30, 31] patients have reported improvements in oxygenation with awake self-proning, its impact on reducing tracheal intubation or mortality is unknown. Studies have shown either conflicting results or are difficult to interpret, as awake self-proning was used in combination with other non-invasive respiratory support [29–31]. In addition, there is a concern about delaying intubation in patients in whom awake self-proning is used [32]. Ongoing RCTs on awake self-proning in C-ARF (ClinicalTrials.gov Identifier NCT04395144, NCT04347941, NCT04350723) may provide further guidance.

### High Flow Nasal Oxygen (HFNO)

#### Expert statement

HFNO therapy should be considered as an alternative strategy for oxygen support.

HFNO should be used in patients who are unable to maintain SpO2 > 90% using oxygen delivery through a venturi mask or may be used in patients with increasing oxygen requirement.

HFNO may avoid the need for tracheal intubation and invasive mechanical ventilation in patients with C-ARF.

#### Discussion

HFNO and non-invasive ventilation (NIV) [full-face mask or nasal mask delivering pressure support plus positive end-expiratory pressure (PEEP)] were initially avoided in patients with C-ARF due to the concern around infectious aerosol generation. However, limited availability of invasive ventilators and ICU beds, favourable experience in small studies and increasing availability of personal protective equipment (PPE) have led to increased use of non-invasive therapies [33–35]. Patients on non-invasive respiratory support need continuous monitoring to avoid any delays in tracheal intubation. A recent clinical practice guideline gave a strong recommendation for the use of HFNO over conventional oxygen therapy in patients with acute hypoxemic respiratory failure (AHRF) to prevent tracheal intubation [36]. Though there has been conflicting evidence regarding the use of HFNO to prevent invasive mechanical ventilation in C-ARF, experts recommended its use [37–39]. However, robust studies regarding the risk of aerosol dispersion, optimal settings, comparison with other non-invasive respiratory supports and outcomes are lacking in C-ARF patients.

### Non-invasive ventilation and continuous positive airway pressure (CPAP)

#### Expert statement

NIV should be considered in patients with mixed respiratory failure (hypercapnia and hypoxemia) and may be used in patients with increased work of breathing which is observed subjectively.

#### Discussion

NIV failure and higher ICU mortality were observed in patients with moderate-to-severe ARDS in a sub-analysis of the LUNG SAFE study including 2813 non-COVID patients receiving NIV [40]. There are inconclusive data regarding the role of NIV in reducing the need for invasive mechanical ventilation or mortality in C-ARF patients, from small retrospective studies [41, 42]. CPAP was used in small retrospective studies with some benefit in reducing tracheal intubation in mild-to-moderate COVID-19-related ARDS [43, 44]. Helmet CPAP is also used for management of C-ARF and recommended over HFNO to limit the exposure of healthcare workers (HCW) to aerosols [45]. However, the evidence on effectiveness of helmet CPAP in C-ARF in reducing the need of tracheal intubation is conflicting [46, 47]. In addition, the helmet interface may not be universally available. Future trials comparing HFNO with helmet CPAP may settle this debate. (ClinicalTrials.gov Identifier NCT04395807).

### Tracheal intubation

#### Expert statement

The appropriate triggers for tracheal intubation include altered mental status, hemodynamic instability and failure to maintain SpO2 > 90% with non-invasive respiratory interventions.

#### Discussion

The decision for tracheal intubation in patients receiving non-invasive respiratory support is challenging, requiring a fine balance between early intubation and risks of invasive mechanical ventilation versus the adverse effects of delaying intubation. The impact of early versus delayed tracheal intubation has not been compared in patients with C-ARF. The decision for tracheal intubation in COVID-19 patients may be best determined using a combination of factors that include clinical acumen, oxygen saturation, dyspnoea and respiratory rate [48, 49]. Experts recommended the use of clinical criteria to be preferred over the use of arterial blood gas or imaging findings to determine the need for tracheal intubation.

### Lung protective ventilation

#### Expert statement

Lung protective ventilation (LPV) should be used for patients with C-ARF on IMV.

The targets for LPV in C-ARF include tidal volume of 4–6 ml/kg of predicted body weight, plateau pressure ≤ 30 cm of H_2_O and driving pressure ≤ 15 cm of H2O.

#### Discussion

Experts agreed that the COVID-19-related ARDS is clinically similar to other forms of ARDS; hence, there was a full agreement for the use of lung protective ventilatory strategies (tidal volume 4–6 mL/kg of predicted body weight and plateau pressure ≤ 30 cm of H_2_O). Severe hypoxaemia with near normal respiratory system compliance, a combination rarely seen in ARDS, had been noted in small studies [6, 7]. However, in large observational multicentre studies, the respiratory mechanics of ventilated patients with COVID-19-related ARDS were noted to be similar to non-COVID-19 ARDS [20–22].

### Recruitment manoeuvres

#### Expert statement

Recruitment manoeuvres may be considered only in selected patients with C-ARF on invasive mechanical ventilation, in view of their potential deleterious effects.

#### Discussion

Diffuse alveolar damage, endotheliitis and microthrombi in pulmonary microvasculature have been reported in small autopsy studies of COVID-19 patients [16–18]. Microthrombi causing hypoxaemia will not respond to PEEP or a recruitment manoeuvre. The experts suggested that recruitment manoeuvres, if ever used should be individualised, in view of the potential harmful effects as seen in non-COVID-19-related ARDS [50, 51].

### Neuromuscular blocking agents (NMBA)

#### Expert statement

NMBA may be considered during the early phase of invasive mechanical ventilation in case of patient-ventilator dyssynchrony.

#### Discussion

Recent meta-analyses have not demonstrated unambiguous benefits on important patient outcomes with the use of NMBA in non-COVID ARDS [52, 53]. It is possible that the impact of NMBA infusion on mortality depends on the strategy used in the control arm. The strong suggestion in favour of the use of NMBA by our experts, in case of patient-ventilator dyssynchrony contrasts with this lack of certainty and may be supported by the relative safety demonstrated so far. Clinical experience from around the world over the last year has demonstrated that it can be difficult to ventilate these patients in the very acute phase without NMBA, thus the apparent discordance between the recommendation of the experts and the literature in non-COVID-19 patients. However, recent guidelines recommend the use of an NMBA infusion for 48 h in patients with refractory hypoxemia despite deep sedation to facilitate lung protective ventilation strategy or prone positioning and/or when there is high respiratory drive despite optimal sedation [54, 55]. There are no published trials evaluating the use of NMBA on outcomes of ventilated patients with C-ARF.

### Prone ventilation

#### Expert statement

Prone position in patients with C-ARF on invasive mechanical ventilation should be used for a duration of 16–24 h per session to improve oxygenation.

#### Discussion

Prone position for ventilated patients with C-ARF was strongly suggested by experts, for a duration of 16–24 h per session, similar to the indication in non-COVID-19-related ARDS [28, 53, 56].

### Veno-Venous extracorporeal membrane oxygenation (V-V ECMO)

#### Expert statement

V-V ECMO may be considered in patients with refractory hypoxemia on invasive mechanical ventilation, who do not respond to other adjuvant therapies.

#### Discussion

These recommendations are in agreement with the WHO and extracorporeal life support organisation (ELSO) guidelines for the management of COVID-19 [24, 57]. Though higher mortality was reported during initial days of the pandemic, there is increasing experience and evolving evidence showing favourable outcomes with ECMO in COVID-19 patients [58–60]. In a recent meta-analysis, the 90-day mortality was lower in non-COVID-19-related ARDS patients on ECMO as compared to conventional ventilation [61]. In the EOLIA trial, the greatest benefit of V-V ECMO was seen in patients with moderate-to-severe ARDS or severe respiratory acidosis after optimisation of ventilator settings [62]. Experts recommend V-V ECMO for patients with refractory hypoxemia when lung protective ventilation and prone ventilation have failed or the latter is contraindicated.

### Infection control

#### Expert statement

Bag mask ventilation, HFNO, NIV, tracheal intubation, open suctioning, bronchoscopy, tracheal extubation and tracheostomy may be considered as aerosol-generating procedures in and outside the ICU.

Airborne infection isolation rooms and video laryngoscopes may be considered during tracheal intubation; a closed suction system should be considered to reduce cross-transmission of SARS-CoV-2 in the ICU.

#### Discussion

There is limited evidence regarding aerosol-generating procedures, or the use of airborne infection isolation rooms, use of video laryngoscopes during tracheal intubation and closed suction systems to mitigate aerosol generation in COVID-19 patients [35, 63, 64]. Simulation studies on aerosol production during tracheal intubation and extubation have provided divergent results [65, 66]. There is conflicting evidence on aerosol generation with NIV or HFNO [35, 67, 68]. The experts have taken a conservative approach, labelling procedures as aerosol generating, until robust evidence is generated to the contrary.

### Weaning from invasive mechanical ventilation

#### Expert statement

Weaning should not be delayed, for the threat of the risk of reintubation.

A pressure support ventilation trial for 30 min to 2 h may be preferred over other weaning strategies.

#### Discussion

Weaning and extubation (in particular the strategy of delaying weaning) are very relevant to COVID-19 patients, due to concerns of the increased risk of aerosol exposure to the healthcare worker, if there is failure of tracheal extubation and need for a reintubation. In addition, there are concerns of aerosol generation with the use of the open T-piece as compared to pressure support ventilation. Nevertheless, the experts were strongly against delaying extubation in order to potentially reduce risks of later reintubation, suggesting the use of similar criteria as in non-COVID-19 patients [69]. The recommendation regarding the weaning strategy is consistent with recent evidence supporting pressure support ventilation for 30 min over T-piece for two hours, although this is not universally accepted [70, 71].

### Early mobilisation

#### Expert statement

Early mobilization may be beneficial in patients on respiratory support for C-ARF.

#### Discussion

Experts suggested that early mobilisation may be beneficial in patients with C-ARF receiving respiratory support; given the evidence that early mobilisation of ICU patients has significant benefits [72].

### Tracheostomy

#### Expert statement

The timing of tracheostomy to facilitate weaning from mechanical ventilation should be the same as in non-COVID-19 patients.

Percutaneous dilatational tracheostomy (PDT) with or without guidance (using ultrasound or bronchoscopic) may be preferred over other techniques.

#### Discussion

The timing and technique of tracheostomy, due to possible aerosol generation or dispersion, have generated intense debate among clinicians [73]. The safe period for performing a tracheostomy in COVID-19 patients is recommended to be 10–21 days after tracheal intubation to reduce infectious risk. [73]. However, no increased risk of infection to healthcare workers was observed, when clinical judgment-based instead of fixed timing tracheostomy was performed with appropriate PPE use [72]. Modifications to tracheostomy techniques are recommended in COVID-19 patients to reduce aerosolisation risk [75]. Although surgical tracheostomy was recommended over PDT, based on experiences from the SARS epidemic, the use of PDT in COVID-19 patients has not shown any increased risk to healthcare workers till date [74].

## Dissensus among the experts on the respiratory management of C-ARF

The following clinical statements did not achieve the desired agreement and stability despite several iterative Delphi rounds. This reflects existing areas of uncertainty.

There was no agreement among the experts that awake self-proning may prevent the need for invasive mechanical ventilation. Experts did not recommend the use of NIV in all patients with C-ARF as an alternative strategy for oxygen support or to avoid the need for invasive mechanical ventilation, unlike with HFNO. In addition, the experts did not agree that HFNO produces fewer aerosols as compared to NIV with face mask.

There was disagreement for the use of non-conventional modes of mechanical ventilation including airway pressure release ventilation and pressure-regulated volume control. There was no agreement for higher versus lower PEEP strategy, nor the method of PEEP selection in these patients. Lung hyperinflation has been reported in small case series of patients with C-ARF with the use of high PEEP [76, 77]. There was disagreement for the effectiveness of adjuvant therapies for refractory hypoxaemia (inhaled nitric oxide, nebulized prostacyclin, etc.). This likely reflects the lack of any demonstrable benefits with any of the salvage therapies, other than prone ventilation [56].

There was no agreement on any combination of PPE over the other. A Cochrane meta-analysis (24 studies with 2278 participants) on the role of PPE in preventing infections among healthcare workers concluded that there was no difference between various types of PPE [78]. There was no agreement on the beneficial effect of chest physiotherapy in patients with C-ARF. Questions related to specific chest physiotherapy interventions were not asked, which may be a limitation. A personalised approach may be required with some of these interventions, depending on the patient, phase of illness and the respiratory mechanics. The benefits of chest physiotherapy in ventilated patients with C-ARF are unclear, with limited evidence on the risks of aerosol dispersion of the virus with some of the therapeutic manoeuvres [79].

## Strengths and limitations

Our work has several strengths. Firstly, our panel included a large number of global experts in the field of respiratory failure, with experience in the management of C-ARF and with diverse geographical representation. Secondly, anonymity of experts and their individual responses were preserved until completion, to avoid inherent bias during the Delphi process due to dominance and group pressure. Thirdly, we were able to successfully complete the process over five survey rounds, maintaining a tight timeline (one month) despite experts being busy during a pandemic, which was essential considering the rapidly evolving evidence. Fourthly, we were able to achieve agreement in 73% of our clinical statements.

Our work has limitations. It is difficult to answer 'yes' or 'no' to some questions, as a personalised approach may be required for some clinical interventions. It is also possible that the responses from the experts could have been influenced by the way they interpreted the statements. The feedback from the experts (allowed in all the rounds) and the stability of the responses should have ensured fidelity of the responses and minimised the risk of responder bias. Secondly, factors such as non-availability of or inadequate experience with some treatment modalities and variation in regional guidelines may have influenced the opinions of experts and affected the generation of statements. Thirdly, some aspects of respiratory management, such as extubation to NIV or HFNO to prevent re-intubation, were not included.

These expert clinical practice statements will provide guidance to the clinician at the bedside. However, several questions regarding the respiratory management of C-ARF remain unanswered and new evidence is being generated at a rapid rate. We have summarized these as future research priorities in Table 2.

**Table 2:** Research priorities in COVID-19-related acute respiratory failure

Pathophysiology	Exploration of “personalised” respiratory interventions based on phenotypes (using clinical, physiological, biological or radiological criteria)
Awake self-proning	Optimal technique (such as complete prone or side to side), timing and duration Impact on escalation of respiratory support, tracheal intubation and mortality Effect of combination with HFNO or NIV on outcome measures
HFNO	Risk of aerosolisation, optimal setting, monitoring and prediction of failure Comparison with NIV/CPAP and weaning strategies Use in moderate-to-severe hypoxemia (PaO_2_/FiO_2_ less than 200 mm Hg) Impact on outcomes (ICU/hospital length of stay and mortality)
NIV (including CPAP)	Risk of aerosolisation, monitoring, helmet versus other interfaces Multimodal strategies with HFNO Impact on escalation of respiratory support, outcomes (ICU/hospital length of stay and mortality) Impact of NIV in subset of patients with mixed respiratory failure, cardiogenic pulmonary oedema and COVID-19-related ARDS
Corticosteroids	Effect of timing of initiation, different types, dose, optimal duration, tapering schedule Impact of laboratory biomarkers on timing, dose and duration of corticosteroid Interaction of corticosteroids with other COVID-19 therapeutics such as Remdesivir, Baricitinib, etc
Invasive mechanical ventilation	Initiation of invasive mechanical ventilation: Optimal timing, triggers and technique with respect to patient and HCW safety Impact of non-conventional ventilation strategies based on respiratory mechanics on outcomes (ICU/hospital length of stay and mortality) Sedation and NMBA: Optimal sedation strategy and monitoring techniques. Timing, duration, technique (continuous versus bolus) and monitoring of NMBA PEEP: Strategy for personalisation and method of selection Fluid management: Restrictive versus liberal. Impact on ARDS phenotypes Assessing fluid responsiveness Weaning and liberation: Optimal timing and strategy. Impact of HFNO or NIV post-extubation. Predictive measures for failure ECMO: Optimal timing and patient selection. Resource planning in the constraints of a pandemic
Tracheostomy	Optimal timing, strategy for HCWs safety and post-procedure care. Direct effect of SARS-CoV-2 virus on larynx and trachea
Infection control	Strategy for HCWs safety during aerosol generating procedures in resource limited settings Role of different types of PPE and strategies to optimize their use De-escalation of isolation precautions: time and/or testing based Impact of different interventional strategies on the reduction in aerosolisation Efficacy and safety of tele-ICU or remote monitoring to limit exposure

HFNO: high-flow nasal oxygen; NIV: non-invasive ventilation; CPAP: continuous positive airway pressure; ICU: intensive care unit; COVID-19: coronavirus disease 2019; ARDS: acute respiratory distress syndrome; HCW: healthcare worker; NMBA: neuromuscular blocking agent; PEEP: positive end-expiratory pressure; ECMO: extracorporeal membrane oxygenation; SARS-CoV-2: severe acute respiratory syndrome coronavirus 2; PPE: personal protective equipment

## Conclusions

Using a Delphi method, an agreement among experts was reached for 27 statements on the respiratory management of C-ARF, addressing important decisions for patient management in areas where evidence is either absent or limited. Strong evidence from high-quality clinical trials is needed to clarify the remaining uncertainties. While these expert clinical practice statements provide clinical direction with C-ARF, some of these general principles may help with the management of other viral pneumonias or future variants of the SARS-CoV-2 strain.

## Supplementary Information

**Additional file 1.** Search Strategy and Selection Criteria.

**Additional file 2.** Details and Results of the Delphi process.

**Additional file 3.** Survey Report 1.

**Additional file 4.** Survey Report 2.

**Additional file 5.** Survey Report 3.

**Additional file 6.** Survey Report 4.

## Data Availability

Supporting data are available with the corresponding author.
