# Supplementary material for: Expert consensus statements for the management of COVID-19-related acute respiratory failure using a Delphi method
Source: Crit Care. 2021 Mar 16;25:106. doi: 10.1186/s13054-021-03491-y (PMC7962430; doi:10.1186/s13054-021-03491-y)
Supplement: Supplementary file 1 — Additional file 1. Search Strategy and Selection Criteria. [file 13054_2021_3491_MOESM1_ESM.pdf]

SEARCH STRATEGY AND SELECTION CRITERIA

A search of literature (English language) was conducted from the database of PubMed, MEDLINE, Embase and internet (Google and Google Scholar) between January 1, and September 3, 2020. We used combination of keywords, “SARS-CoV-2”, “novel coronavirus”, “COVID-19”, “pathophysiology”, “acute respiratory failure”, “adult respiratory distress syndrome”, “non-invasive ventilation”, “high frequency nasal oxygen”, “tracheostomy”, “steroid”, “tracheal intubation” “tracheal extubation”, “personal protective equipment”, “aerosol generating procedures”, “infection control”, “weaning”, “prone ventilation”, “awake proning”, “recruitment manoeuvre”, “refractory hypoxaemia”, “positive end expiratory pressure, and “extra corporeal membrane oxygenation” for this search. We excluded search results which have non-human study subjects, non-English literature, paediatric population, and also publications in the form of, abstracts. Guidelines for management of COVID-19 published by WHO, European Centre for Disease Control and Prevention (CDC) US CDC, European Society of Intensive Care Medicine and Society of Critical Care Medicine were also reviewed. The final reference list was then reviewed by steering group based on the relevance to topics covered in this Health-care Development paper. The final pool of the literature (454 articles) was stored in “Google Drive” cloud for reference among steering committee members and experts.
