# Supplementary material for: Expert consensus statements for the management of COVID-19-related acute respiratory failure using a Delphi method
Source: Crit Care. 2021 Mar 16;25:106. doi: 10.1186/s13054-021-03491-y (PMC7962430; doi:10.1186/s13054-021-03491-y)
Supplement: Supplementary file 2 — Additional file 2. Details and Results of the Delphi process. [file 13054_2021_3491_MOESM2_ESM.pdf]

DETAILS AND RESULTS OF THE DELPHI PROCESS

Delphi process

A steering committee of 10 critical care physicians (PN, EA, SG, RJ, YJ, DJ, AKK, PR, KS, and SNM), actively involved in the management of patients with C-ARF from Australia, France, India, the United Arab Emirates (UAE) and the United States of America (USA) was formed in August 2020. The proceedings of the committee were conducted by a moderator (SNM) through e-mail and video conferences. A modified Delphi process[14, 15] was used to generate agreement on the respiratory management of C-ARF. The study was registered with Clinical trials.gov Identifier: NCT04534569.

The steering committee recruited and convened an international group of intensivists with expertise in the field of acute respiratory failure, based on the following predefined criteria as on 30th August 2020: 1. Publications in PubMed indexed journals in the field of acute respiratory failure; 2. H-index >10 sourced from Scopus® author search; and 3. Clinical experience in the management of patients with C-ARF. A concerted effort was made to ensure geographical, ethnic and gender diversity among the invited experts.

E-mail invitations were sent to 60 experts, to participate in the Delphi process. Upon acceptance, the experts were included in the Delphi process to generate agreement. Their identity was anonymised from other experts until completion, to avoid bias due to dominance or group pressure. Surveys disseminated to the experts were prepared using Google Forms.

The overall scope of the project was determined through a systematic search and review of available literature on C-ARF by the steering committee, published between January 1st and September 3rd 2020. Several video conferences of the steering committee were convened to prepare a list of interventions for the respiratory management of C-ARF, where the committee felt that clear evidence was lacking. The list was presented to experts in the form of a survey questionnaire (round one), which included five sections: non-invasive respiratory interventions; invasive mechanical ventilation; refractory hypoxaemia; infection control; weaning and tracheostomy. The experts subsequently responded to the questionnaire and provided text feedback using the comments section available for each question if required.

The steering committee held video conferences following each round, to discuss the results, plan the next survey questionnaire and amend any questions. A report of the results of each round was prepared and submitted to the experts with the subsequent round of the survey questionnaire. All rounds of these survey questionnaires were conducted using an iterative approach, adapted from the Delphi method, to prioritise topics for inclusion, and repeated until agreement and stability were achieved as described below.

Agreement and stability

For statements with responses on an ordinal 7–point Likert scale, ‘agreement’ was defined as a score of 5-7, ‘neutral’ by a score of 4 and ‘disagreement’ by a score of 1-3. Agreement was defined as achieved when >70% of the experts voted for a given option a Likert scale statement.[16,17] Median and interquartile range (IQR) were used to describe the central tendency and dispersion of responses. For multiple-choice questions (MCQs), agreement was defined as achieved if >80% of the experts voted for a particular option. Stability in the responses was assessed from round three onwards. Stability was assessed between the two concluding rounds for each statement, using the non-parametric chi square ($\chi 2$) test. $p < 0{\cdot}05$ was considered as a significant variation or unstable.

Statements which achieved both agreement and stability and those that were unlikely to get agreement on further rounds (scattered responses on the Likert scale or MCQ in the first three consecutive rounds) were removed from the subsequent rounds. Data from the last stable questionnaire round of Delphi process for each statement were included for preparing the final expert statements.

Clinical Practice Recommendations

Clinical practice recommendations were derived by the steering committee, from the statements that generated agreements through the Delphi process. The clinical practice recommendations, were considered to be “strong statements” when a median of ≥6 or ≤2 on the Likert scale or >90% votes for any MCQ option were achieved.[14] For the clinical practice recommendations, the term “should” was used for the strong statements and “may” was used for the other statements.

The final results of this survey and the clinical practice points were circulated among the experts. The manuscript was circulated among the experts for editing and approval before it was submitted for publication.

RESULTS

Panel of experts

Of the 60 experts invited, 39 (65%) from 20 different countries and six continents participated in the Delphi process. Thirty-seven (95%) completed all five rounds (Figure 1). The median age of the experts was 53 (13) years and 5 (13%) were female. Majority (92%) of the experts were affiliated with university hospitals; the median h-index was 33 (11-100).

Delphi process, expert statements and clinical practice points

Five survey questionnaire rounds were conducted between 4th September to 5th October 2020. The first round survey questionnaire was open ended. This generated a total of 529 comments which were used to draft round-two of the survey questionnaire, which included 37 statements. In round three, the same statements were repeated, ten of which were rephrased for clarity. In the round-four survey questionnaire, seventeen statements which already achieved agreement and stability along with nine statements which were unlikely to generate agreement were removed, leaving eleven statements for inclusion. Two of these statements remained unstable but achieved stability in round five (conducted by email, no survey link was sent). At the end of the Delphi process, 27 statements (73%) achieved agreement and stability (expert statements) from which 20 clinical practice points were prepared. Details of the Delphi rounds are provided in Figure 2. Detailed analysis of the final results of all 37 survey questionnaire statements used in the Delphi process are given in the Table 1. Reports of the first four survey rounds are provided in the appendix section (Appendix 1: Survey Report 1, Appendix 2: Survey Report 2 and Appendix 3: Survey Report 3 and Appendix 4: Survey Report 4). The 20 clinical practice points are given in Figure 3.
