# Supplementary material for: Expert consensus statements for the management of COVID-19-related acute respiratory failure using a Delphi method
Source: Crit Care. 2021 Mar 16;25:106. doi: 10.1186/s13054-021-03491-y (PMC7962430; doi:10.1186/s13054-021-03491-y)
Supplement: Supplementary file 3 — Additional file 3. Survey Report 1. [file 13054_2021_3491_MOESM3_ESM.pdf]

Report of Survey Questionnaire One

Section-1: Non-invasive Respiratory Interventions

1. The pathophysiology of COVID-19 related acute respiratory failure (C-ARF) is similar to that of acute respiratory distress syndrome (ARDS).

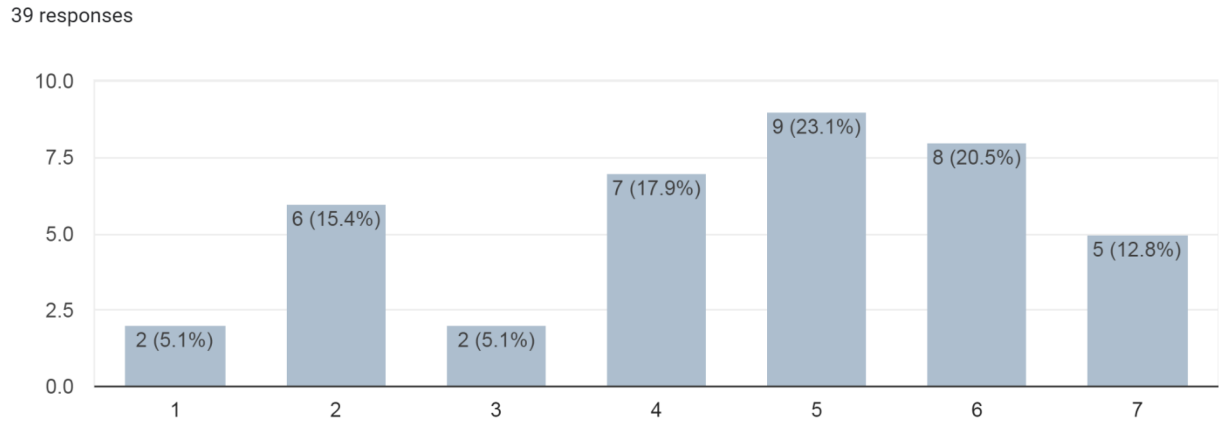

Comments:

1. Type H COVID-19 might be similar to typical ARDS. However, type L, which is characterized by GGO in chest CT, a normal respiratory tract compliance, very good response to recruitment and/or prone positioning and/or PEEP, might it really different from ARDS
2. Although there are lot of similarities there is some suggestion that some of the pathophysiological features are more pronounced in C-ARF
3. It depends. I believe there are different phenotypes. ARDS is a syndrome and not a disease as such there are many causes and comorbidities playing a role. As with ARDS we separate between pulmonary vs extrapulmonary.
4. This is like saying the pathophysiology of CAP ARF is similar to ARDS. I'm not sure this is correct nor important. I selected '4' because I don't really understand the statement
5. Timing and severity influence the diagnostic severity, but definitions of ARDS remain the same. More thrombosis in C-ARF than pre COVID
6. Different mechanisms though some common points
7. In the early phase of COVID19 it is different as it is more an endothelial/Capillary affecting disease than an alveolar disease. Lung Compliance is therefore normal in most patients. In the later stage of the disease due to hyperinflammation, secondary infections, or treatment complications this changes and can be closer to classical ARDS
8. ARDS is a syndromic condition. COVID-19 is a viral pneumonia with much vascular involvement
9. As our studies are showing and will show - once all papers published - ARDS is characterized by lesion of alveolar capillary barrier and oedema - with atelectasis - recruitable - and response in oxygenation following better aeration ; in COVID-19 mainly pneumolysis and vascular lysis - alveolar cellular infiltration or mucocinosis - thus no atelectasis - and poor recruitment -the excess tissue mass is around 800 g in COVID and similar to ARDS - but in COVID only 3 per cent of excess tissue mass is recruitable while 30 percent in traditional ARDS - in some way we can define COVID -19 as typical primary ARDS - as we defined in 1999 and 2003 ARDS no oedema.
10. There are some differences in phenotypes. In situ thrombosis is different.
11. It is similar when compared to ARDS resulting from for instance influenza pneumonia, but different from trauma, blood transfusion related ARDS

12. To be ARDS, there should be increased permeability of alveolar epithelial and capillary endothelial cells. Therefore, it should be discriminated into severe bilateral COVID-19 pneumonia with COVID-19 pneumonia/sepsis-related ARDS.
13. Shown in 2 publication on Critical Care (CC) and Lancet Respiratory medicine (LRM) from the Italian group
14. It is dangers for the patient, because can delay the intubation
15. Patients with Severe COVID-19 can progress to a syndrome identical to ARDS
16. Usually higher respiratory compliance, high dead space and major hypoxemia already early in the course of the disease

2. Patient self-inflicted lung injury (P-SILI) contributes to poor outcomes in C-ARF.

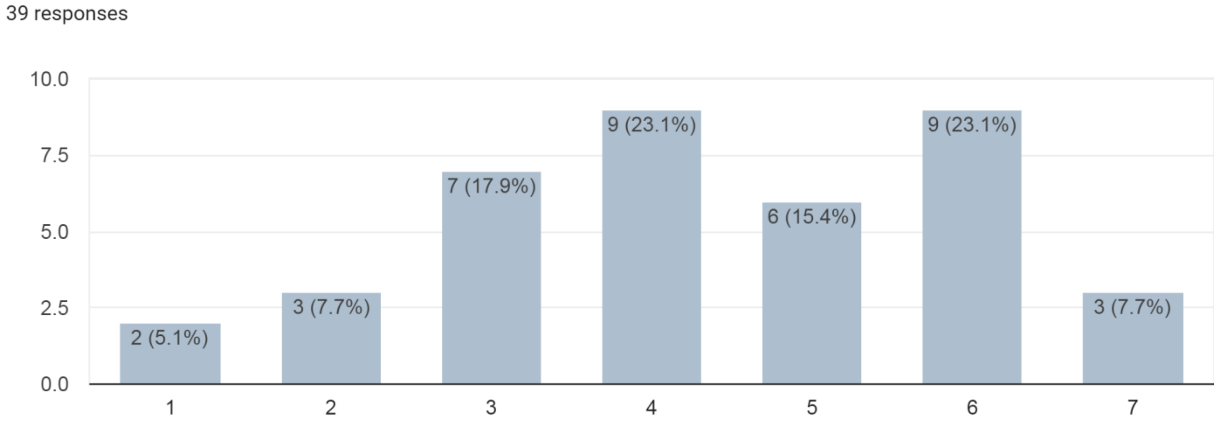

Comments:

1. Not very sure about this. Spontaneous breathing, often associated with significant inspiratory effort, large tidal volume and high driving pressure, may be related to lung injury (P-SILI). However, evidence supporting this hypothesis, especially in patients with L type COVID-19.
2. For some reason the respiratory drive is enormous leading to huge pressure swings.
3. Yes, I agree, but don't think this is unique to C-ARF. it applies to all CAP
4. We don't know as no trial could measure it effectively, so hypothetical
5. It makes sense in physiopathology perspective and also from my clinical observations
6. Too weak data, which need to be confirmed
7. Where is this data?
8. Not clear effects of P-SILI on pathophysiology of COVID - being different from traditional ARDS - for sure we must closely monitor patients undergoing assisted ventilation and characterized by increased effort - as for other forms of hypoxemic respiratory failure - by respiratory rate - saturation in oxygen trends - dyspnoea (happy hypoxia) - activation of accessory respiratory muscles - lung and diaphragm ultrasound - specific suggestions have been published also by our group - **(Text deleted to keep anonymity)**
9. Patient-ventilator dyssynchrony at any cause could be associated with P-SILI and poor outcome.
10. P-SILI is still a matter of controversy, without real solution
11. No convincing evidence to support this hypothesis
12. We don't know
13. While duration and depth of dead space abnormalities have been more severe, mortality has not been as high as initially reported
14. Very high respiratory drive is frequent but not constant in COVID-19 patients with acute hypoxemic respiratory failure

3. Based on your experience, awake self proning can avoid the need for INVASIVE MECHANICAL VENTILATION in patients with C-ARF.

39 responses

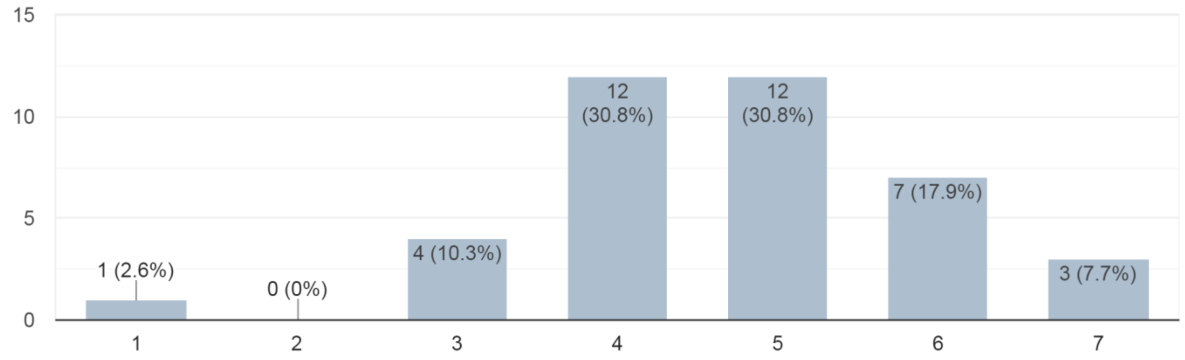

Comments:

1. Evidence here is not strong enough but may be true.
2. We tried this out in a medium care unit. Depends on case mix and DNR orders (step-up vs step-down)
3. I don't have enough experience with this to agree or disagree
4. Maybe in some cases. worth trying if tolerated. similar to JAMA and BMJ experience
5. Early treatment with less chance of P-SILI, this should be the two main benefits of early proning in SB
6. Together with oxygen supplementation
7. Very sceptical about this intervention, unless a well-designed and conducted trial shows benefit
8. Prone position in COVID have different effects as compared to traditional ARDS - on oxygenation - see my explanation above - responses in PEEP and prone (in SB or controlled mechanical ventilation) - in traditional ARDS many due to alveolar recruitment - in COVID - redistribution of perfusion - thus prone position might be considered as a rescue therapy - under careful monitoring and for a relatively short time of application - further it might be associated with delayed intubation - often lost the effects after repositioning supine be careful
9. Proning improves oxygenation. The effect on the need for intubation is uncertain.
10. Haven’t used it enough to have a clear opinion
11. Most patients could not tolerate the prone position under awakened.
12. It has been shown that maybe it doesn't change the mortality, but reduces the need of invasive ventilation
13. In some subsets of patients which we need to learn to identify better
14. We do not know, but experience was mixed
15. Your question needs a RCT
16. Not always tolerated but when tolerated very efficient in improving hypoxemia
17. Is useful but the patient needs to be able to tolerate it. Sitting the patient up and getting them to stand often extremely useful adjuncts. We evaluate clinical status on an ongoing basis to assess tolerance and work of breathing to avoid exhaustion

4. When should awake self proning be initiated in patients with C-ARF?

39 responses

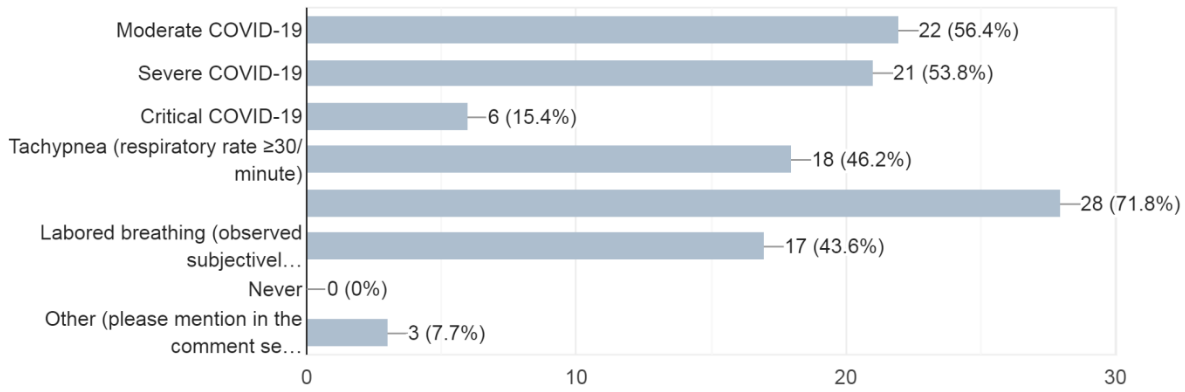

Comments:

1. Whenever patient is hypoxic; multiple options chosen since it is best to implement as soon as the patient is admitted irrespective of the severity unless it is mild...all other categories should be encouraged awake proning
2. Would suggest trying it in any C-ARF patient that is collaborative and able to understand what is asked for. Any patient with extra oxygen supplementation should try at least one documented (saturation followed) session of self-prone
3. Worth trying early, but at any stage, if tolerated
4. I marked both moderate and severe, but this is based on clinical judgement of a single case. some moderate may benefit, some severe (early) too
5. We simply do not know
6. Carefully - often lost effects after repositioning supine - see my comments above
7. Low cost, easily applicable. But not all patients accept it.
8. When a patient required high flow of oxygen by nasal prongs or mask and before even using HFNC or NIV
9. It can always be initiated as a trial unless there is a clear need for mechanical support
10. Depends on services available at the institution
11. Have seen some really positive responses and patients avoid the need for intubation
12. No comment
13. Patients with hypoxia and extensive lower lung zones involvement
14. If patient improves subjectively, would continue
15. We normally want a cooperative patient who can be monitored closely and evaluated on an individual patient basis. In many patients we get them to sit and even stand if possible, which we have found to be extremely useful

5. Based on your experience, lateral positioning (left or right lateral decubitus) is as beneficial in improving OXYGENATION as awake self proning.

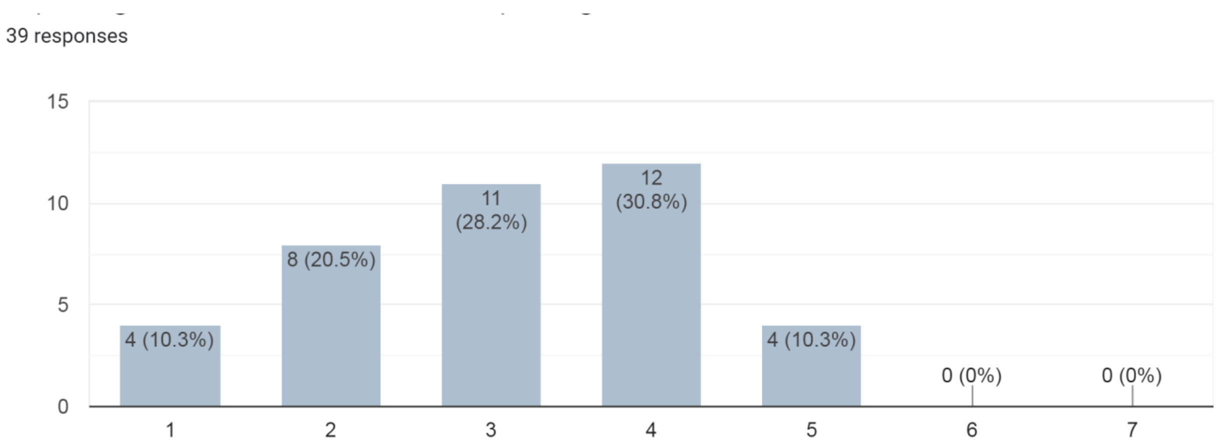

Comments:

1. It depends on the symmetry or asymmetry of lung lesions. If you have for instance the right lung that is most affected it helps to put patient on the "good" left lateral position to improve oxygenation/ventilation mismatch
2. Did not have any experience
3. I don't have experience with this
4. I don't know.
5. Few data. we used it with benefit (also for staff) given the lowered enthusiasm for pronation in later stage of pandemic
6. It works for a small amount, but much worse than prone positioning
7. No
8. Yes - to be considered according to phenotype **(Text deleted for anonymity)** - this might be also by lung US at bedside
9. In some patients lateral; positioning may make some improvement
10. Should be used only when patient is unable to sustain awake proning either because of abdominal condition or intolerance
11. Less effective but can improve oxygenation - especially obese
12. When dominantly unilateral lung severity is observed.
13. No clear and significant report
14. In my experience, this strategy does not work well except in very rare and specific cases.
15. Have found it to be useful in some patients

6. Based on your experience, awake self proning can improve SURVIVAL in patients with C-ARF.

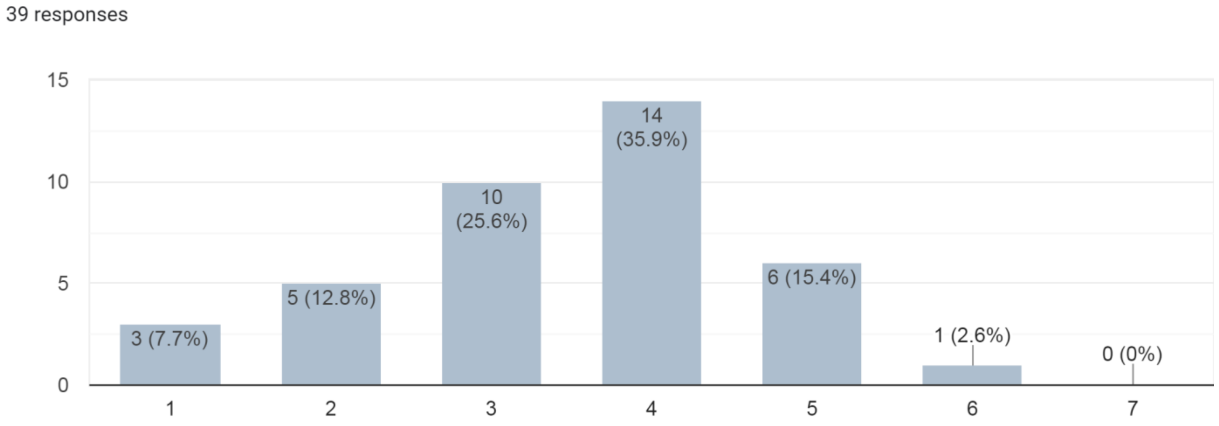

Comments:

1. Not enough data
2. I don't have experience with awake self proning
3. In those who can tolerate for long enough and are SpO2 responders, it may prevent Mech Vent, but unconvinced re survival effect.
4. We need data. clinically it seems of some benefit, but not in all patients
5. It may reduce the intubation rate. However, German data show no difference between intubated and ventilated patients and patients on non-invasive ventilation. Altogether, over the month since COVID19 appeared ventilation strategies had been adapted and the outcome is much better than before.
6. We have no data - more severe patients were awake prone - making difficult any evaluation
7. We do not know this
8. improvement in survival is not documented
9. Not enough experience
10. Depends on capacity available in that region/institution
11. Not seen any good data though
12. Few experiences, to try self proning
13. As written a benefit seems present in observational studies, but RCT are lacking
14. No data. I do not like to guess....
15. We don't know
16. Possibly but not proven in the absence of control group in our cohort
17. Have had patients with definite improvement and favourable outcomes

7. High flow nasal oxygen (HFNO) can be considered as an ALTERNATIVE STRATEGY for oxygen support before invasive mechanical ventilation.

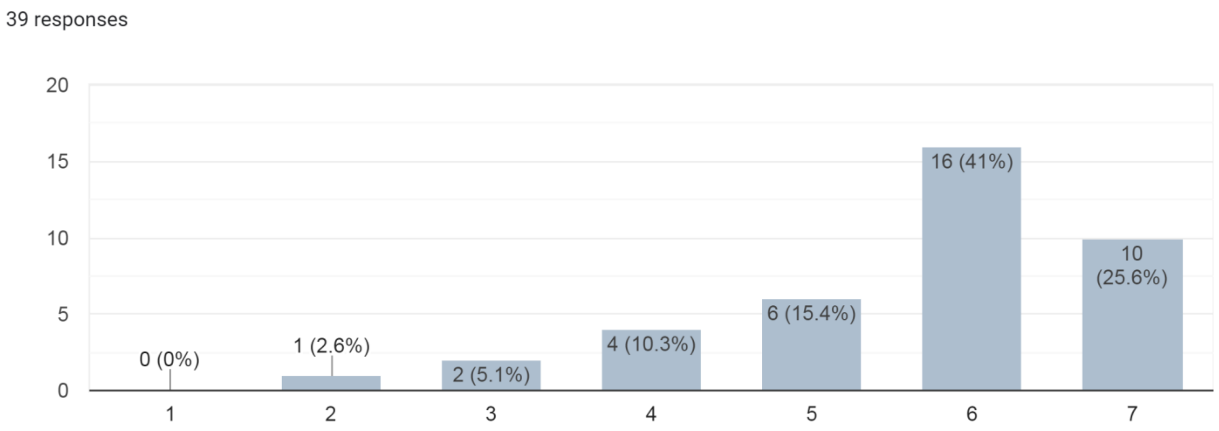

Comments:

1. We start with standard oxygen treatment with face mask and we have put all patients with increasing oxygen needs and C-ARF on HFNO with good result
2. In a neg pressure area or where full PPE is available to staff. Also need to consider the effect on other patients of AGP. However, I think that this was not used enough, early on in London/UK due to concerns for AGP (not unfounded) and also O2 supply issues.
3. Once again, few data. The C-ARF has different patterns, so "conventional" treatments may not be beneficial. perplexities in using HFNO, at least in the beginning of pandemic, increase confusion and lack of data.
4. There is no study to confirm this, but it is daily experience.
5. It may only delay intubation.
6. Of course, yes as non-invasive CPAP (by helmet or other interfaces) - always take into consideration the limitations expressed above.
7. YES. It should be tried in almost all patients.
8. Early and be careful about the aerosolization problem.
9. 100% of our patients are treated with HFNC if they worsened.
10. Maybe but with strict criteria to determine whether another more invasive option has to be initiated. In addition, strong concern about the risk of viral aerosolization and the risk of contamination in the healthcare workers.
11. We have found it to be useful.

8. When do you initiate HFNO in patients with C-ARF?

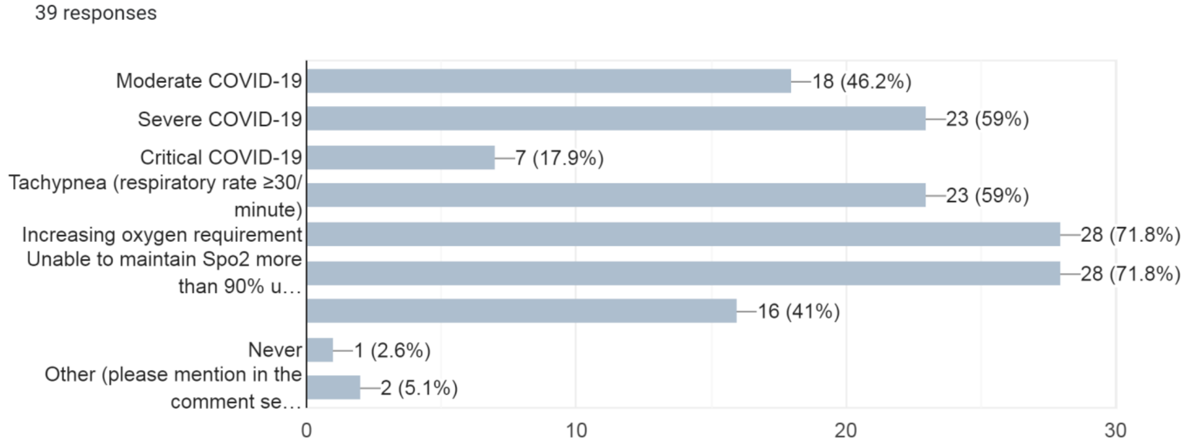

Comments:

1. I would start it based on clinical appearance and oxygenation.
2. When possible early as per FLORALI and if allowable and infection prevention safeguards present - otherwise concern.
3. In my institution use of HFNO was limited per protocol.
4. A mix of the above and depending on availability.
5. Consider my comments above.
6. I am a bit worried still about the risks to staff from aerosols
7. No comment
8. Could maybe be of interest in the non-severe cases but we are afraid of the risk for the care workers in our institution. Performed only if we have a room where a negative pressure environment is available. Overall seldom used in our hospital in COVID-19 patients.

9. Based on your experience, HFNO can avoid the need of INVASIVE MECHANICAL VENTILATION in patients with C-ARF.

39 responses

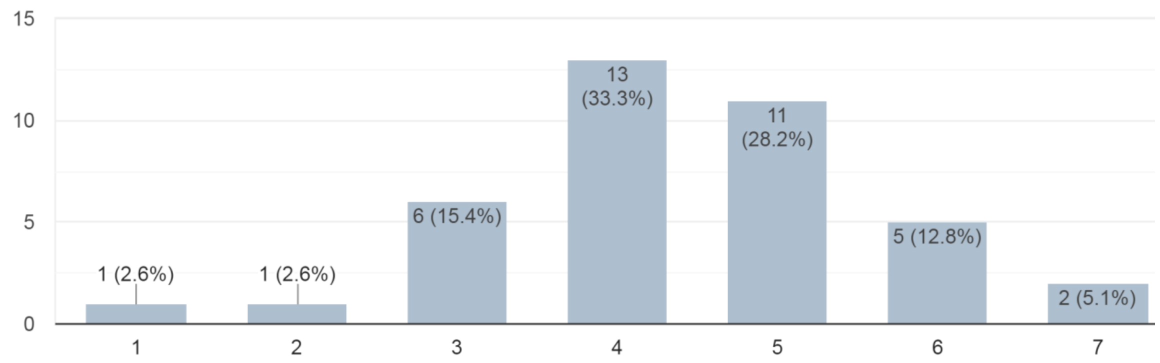

Comments:

1. The avoidance of mechanical ventilation is not dependent on just one factor but a package of interventions that probably result in a reduction in the need for invasive mechanical ventilation.
2. Probably, **(Text deleted for anonymity)** but wasn't able to use it.
3. No evidence, but I guess no. the different evolution is in individual response, how early we start treatment and care to avoid P-SILI.
4. I have no experience with this, however, increase in pulmonary pressure is a minor problem in COVID19.
5. No.
6. And also, CPAP in about 80 per cent of patients - if treated early.
7. In my experience, HFNO is associated with 25% intubation rate.
8. We haven't used HFNO so much.
9. The results are controversial, and some studies have some biases.
10. It would seem this way but there is no data because we have no control group that was hypoxemic/had increased work of breathing and did not receive this treatment.
11. We don't know - needs a RCT
12. 50:50 in our institution
13. Not enough experience with this technique in COVID 19 patients to answer.
14. In some patients - We monitor and evaluate carefully to avoid patients becoming exhausted and intervene earlier rather than later if mechanical ventilation is felt necessary and the patient not settling or deteriorating.

10. Based on your experience, HFNO can improve SURVIVAL in patients with C-ARF.

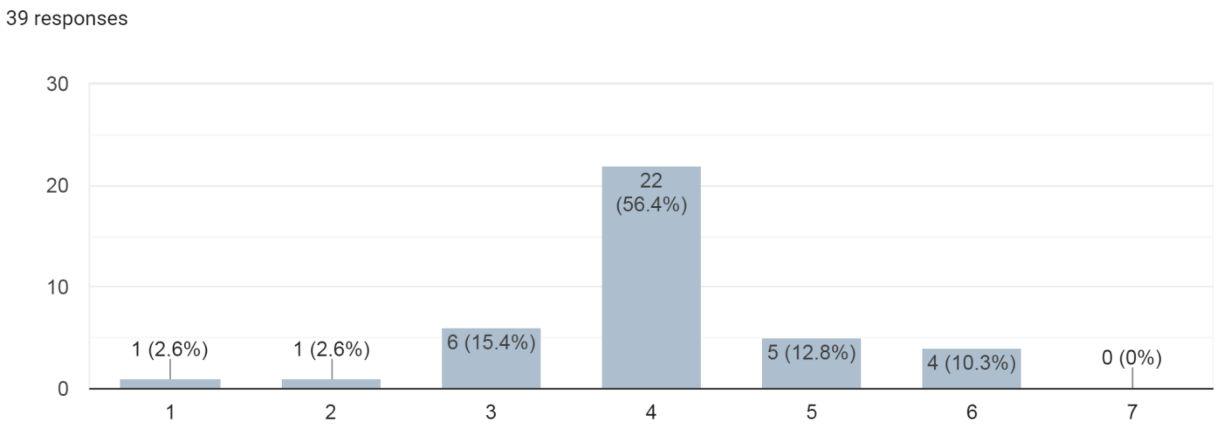

Comments:

1. It is a package of interventions rather than HFNO alone since this only reverses hypoxia and its consequences rather than a direct effect on the pathophysiology of C-ARF
2. Unknown, but possibly as with FLORALI data
3. See above
4. See the question before
5. No
6. I think yes - but not clear data available
7. Probably yes, but data are needed
8. Even about that the results are controversial both on COVID and general population
9. No idea
10. We don't know - needs a RCT
11. Not enough experience with this technique in COVID-19 patients to answer
12. We have had several favourable outcomes

11. Non-invasive ventilation (NIV) can be considered as an ALTERNATIVE STRATEGY for oxygen support before invasive mechanical ventilation.

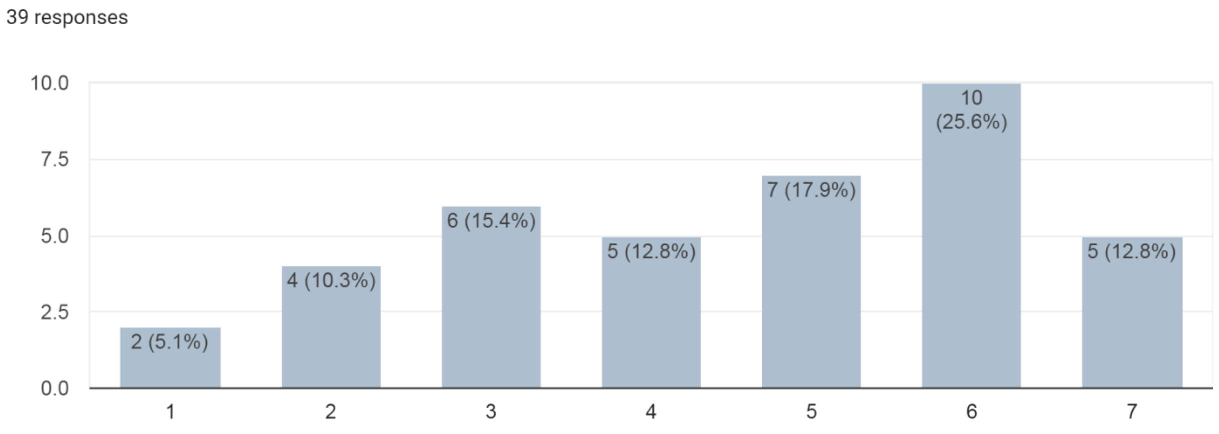

Comments:

1. NIV if used appropriately can alter the course in COVID ARDS.

2. We used it in patients with high respiratory rate and increasing pCO2, so an underlying combination with COPD or cardiac decompensation. We used a helmet or full-face mask to avoid aerosol production.
3. For a short period, and maintaining lung protective ventilation.
4. As a trial for a few hours (2-4), but an escalation plan.
5. Providing adequate timing and respiratory monitoring.
6. In terms of hypercapnia, yes. In terms of hypoxemia without hypercapnia I prefer HFOT.
7. No.
8. If we consider CPAP yes - I do not think as NPPV.
9. Yes, especially if given through helmet (CPAP or NIV)
10. More experience with this seems less aerosol generating. However, still tend to avoid
11. hypoxemic patients could not tolerate NPPV.
12. Short trials of HFNO and NIV before IMV could be attempted
13. Some patients respond well to CPAP.
14. We don't know - needs a RCT
15. Short term
16. I am afraid of the patient self-inflicted lung injury in this situation as the COVID 19 patients usually have intense respiratory drive. I am also afraid of the risk of viral dissemination in the environment because of the leaks
17. Well CPAP is cheap and easy to use.

12. NIV can be considered in the event of HFNO therapy FAILURE for oxygen support in C-ARF.

39 responses

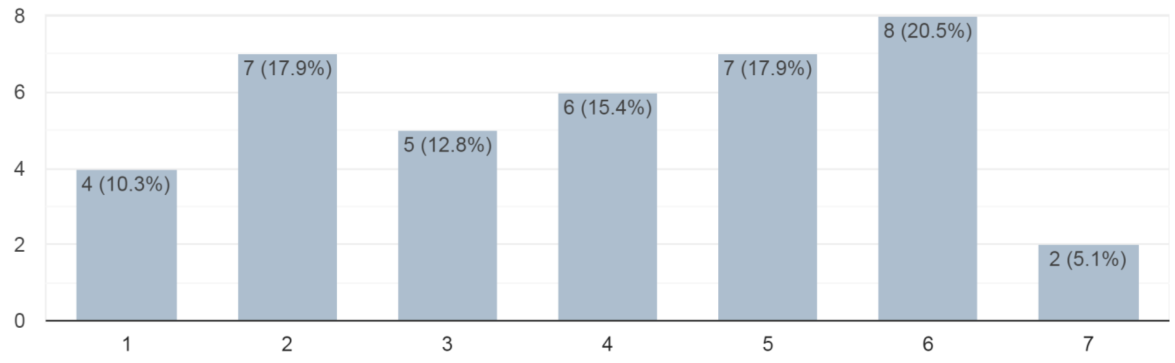

Comments:

1. Yes, but also before HFNO if RR is high or pCO2 increases.
2. Only in those with known COPD as well or hypoventilation, or obese.
3. Yes, given low use/potential and lack of evidence for HFNO.
4. If a patient fails HFNO, she/he likely warrants intubation.
5. No.
6. If HFNO fails usually also CPAP fails - in general the importance is to use non-invasive respiratory support - according to local availability and organization.
7. NIV should also be considered when there are clinical signs of fatigue or ABG evidence of CO2 retention.
8. It could delay switching invasive MV support, leading to poor outcome.
9. See previous comment.
10. We usually alternate between the two if the patient can tolerate it and HFNC alone is not enough.
11. We don't know - needs a RCT.
12. Would proceed to intubation.
13. High risk of P-SILI with few arguments for using this technique in case of severe hypoxemic acute respiratory failure.

14. We may offer a brief trial but have a low threshold for invasive mechanical ventilation if the patient does not appear to be settling or improving.

13. When do you initiate NIV in patients with C-ARF?

39 responses

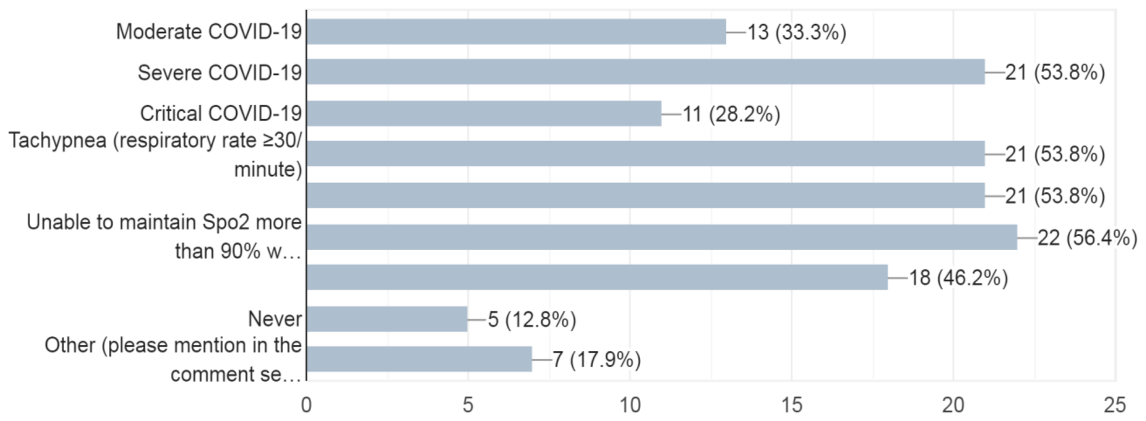

Comments:

1. In some of these situations (e.g. laboured breathing, tachypnoea), NIV can offload the respiratory muscles and delay respiratory fatigue. In the other situations of hypoxia (Moderate, Severe or Critical), in addition, it improves oxygenation
2. Increasing pCO2 - comorbidity: COPD or cardiac failure (HFrEF with pulmonary oedema)
3. See my comments above
4. We triaged use of NIV on P/F ratio and lung ultrasound
5. COPD/obesity hypoventilation
6. We seldom use NIV in this scenario
7. See my comments above
8. Only associated with hypercapnia at moderate severity
9. No comment
10. Continued deterioration despite self proning and HFNC
11. As part of a clinical trial, in selected patients meeting eligibility
12. Rarely
13. Patients are individualized and also depend on resources, availability of HFNO. Have found it useful but as with HFO do not persist if patient does not improve or stabilize

14. Based on your experience, NIV can avoid the need of INVASIVE MECHANICAL VENTILATION in patients with C-ARF.

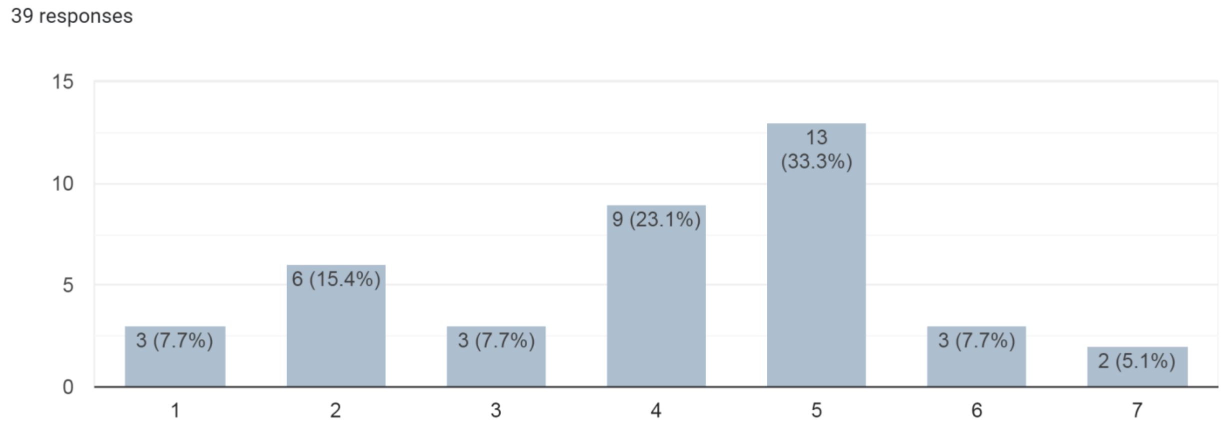

Comments:

1. I think yes - with limitations expressed above
2. Again, no data. But my feeling is that most patients that require this much support will ultimately require intubation
3. No
4. This includes providing time for end of life discussions with patients in whom invasive mechanical ventilation is unlikely to prolong quality of life
5. No personal experience as we did not use NIV in our patient cohort
6. In selected patients, though not all criteria to address these patients may still be clear
7. We dont know - needs a RCT
8. In a number of cases - yes
9. In some instances
10. The problem is the lack of structured RCT

15. Based on your experience, NIV can improve SURVIVAL in patients with C-ARF.

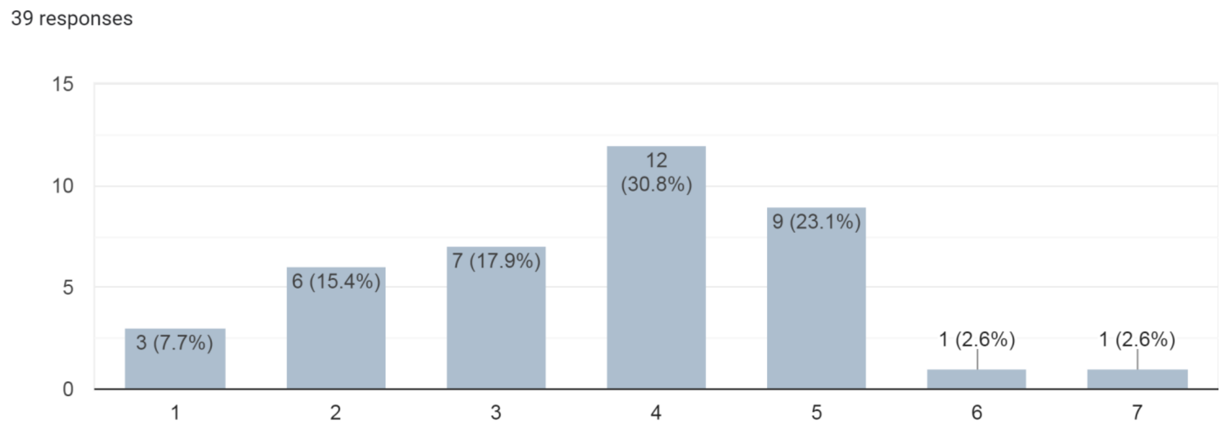

Comments:

1. I think yes - with limitations expressed above

2. I suspect that the lung damage incurred by high CPAP pressures is similar or almost similar to that incurred by positive pressure ventilation with an ET tube
3. No
4. No personal experience as we did not use NIV in our patient cohort
5. Timing, setting and monitoring make the difference
6. We don't know - needs a RCT
7. In those with a favourable initial response
8. Have had some favourable results
9. Data are needed
10. My experience says yes, but studies are needed

16. The use of systemic steroids can avoid the need of INVASIVE MECHANICAL VENTILATION in C-ARF.

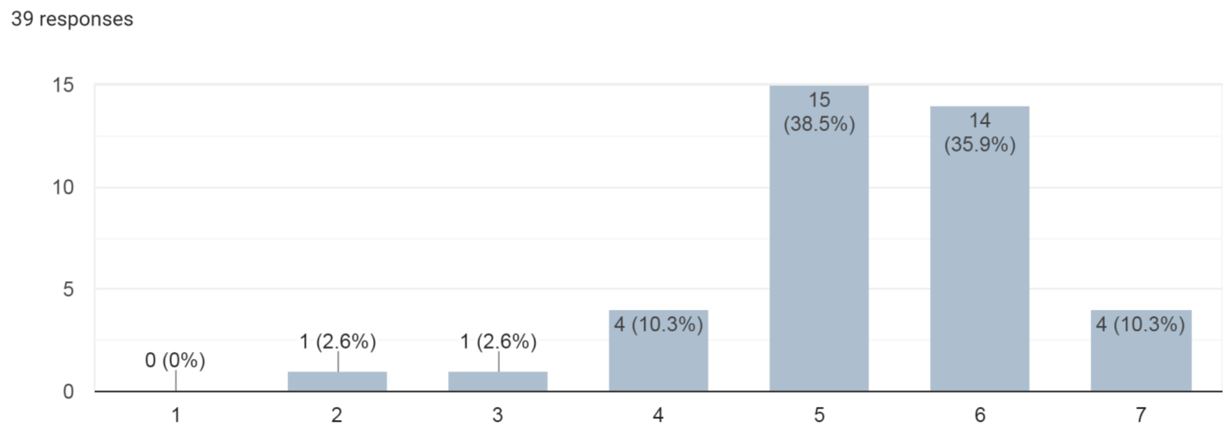

Comments:

1. Absolutely - we started since the beginning - when we worked without any knowledge about COVID - and we were successful - The triad: 1) corticosteroids; 2) coagulation protocols (from prophylactic to therapeutic) 3) protective respiratory support management (from non-invasive to invasive)
2. In some patients. And high dose Meduri protocol. not Oxford protocol
3. Only if the patient is oxygen dependent, most effective in the critically ill ones
4. My response is based on recent publications
5. No
6. This is possible but I don't have enough experience with the treatment to answer, in addition the published data are still controversial. Nevertheless, we now administer steroids, which was not the case at the beginning of the pandemia.
7. We noticed good responses (also measured as cytokines) especially in more severe patients
8. Potentially
9. Yes RECOVERY TRIAL and our experience
10. We have found steroids to be useful
11. Data from RECOVERY and other trials
12. Limited data so far but only in selected patients
13. The timing of initiation of steroids is probably crucial (see next section)
14. Please see the 3 recently published trials on JAMA and The Who meta-analysis /recos

17. When would you choose to initiate steroids in C-ARF?

39 responses

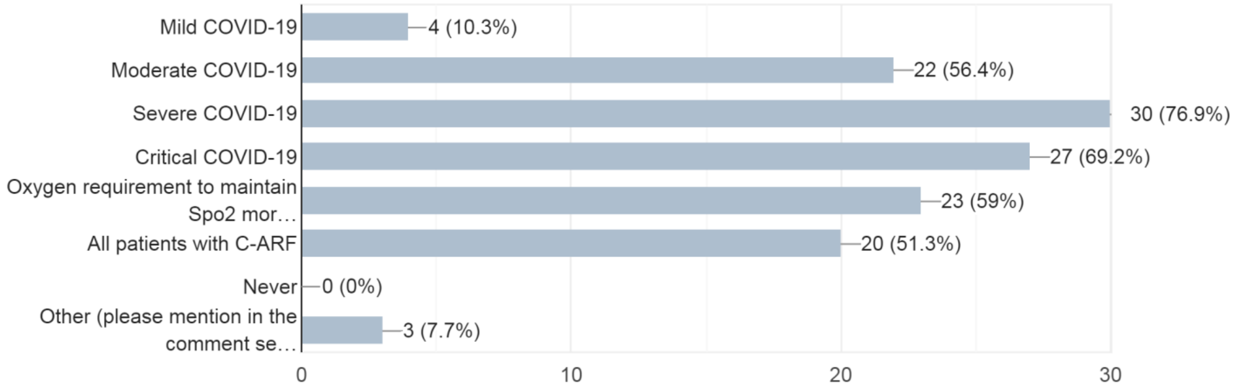

Comments:

1. According to actual recommendations
2. Patients with pneumonia with CRP .20
3. With ongoing active disease >5 days
4. We seldom use these drugs
5. Taking into consideration inflammatory markers (CRP etc).
6. I marked also C-ARF given these patients are in mechanical ventilation
7. Nearly all our ICU patients and all on VV ECMO
8. Early use favoured
9. In the setting of cytokine storm
10. The key is to not use this indiscriminately and to use it at the appropriate setting. It is best to start if there is any evidence of resting hypoxia or exercise induced hypoxia. In the latter setting, initiation of steroid therapy can be delayed if the drop in oxygen saturation is mild (1-2 mm) and there is no radiological evidence of pneumonia
11. None

18. Which steroid do you prefer to use in C-ARF?

39 responses

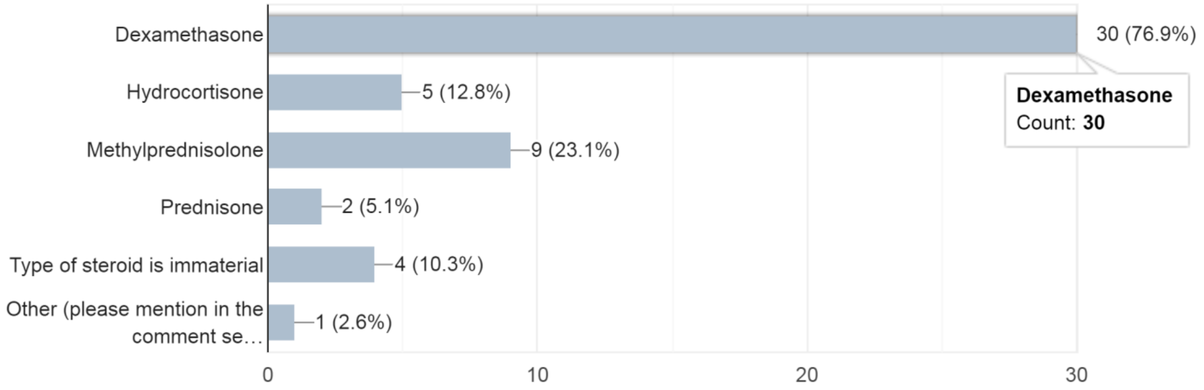

Comments:

1. We used Methylprednisolone - at 1 mg/Kg and than tapered after one week and one week again for a total of three weeks - however we recognize the results from Recovery Trial on Dex - we had very good outcomes - in very severe COVID intubated patients with mortality at 28 days - 28 percent survival and 38 percent in hospital
2. According to previous report
3. See above
4. Based on the published results
5. Our own Institute use is Methyl Prednisolone have used dexamethasone since recovery **(comment modified for anonymity)**
6. Is likely to be a class effect. We give dexamethasone when available and based on the evidence, but have been using hydrocortisone and prednisone as well in equivalent dosages.
7. these are the 2 steroids that showed benefits

19. What daily dose of corticosteroid (equivalent dose of dexamethasone) you prescribe for C-ARF?

39 responses

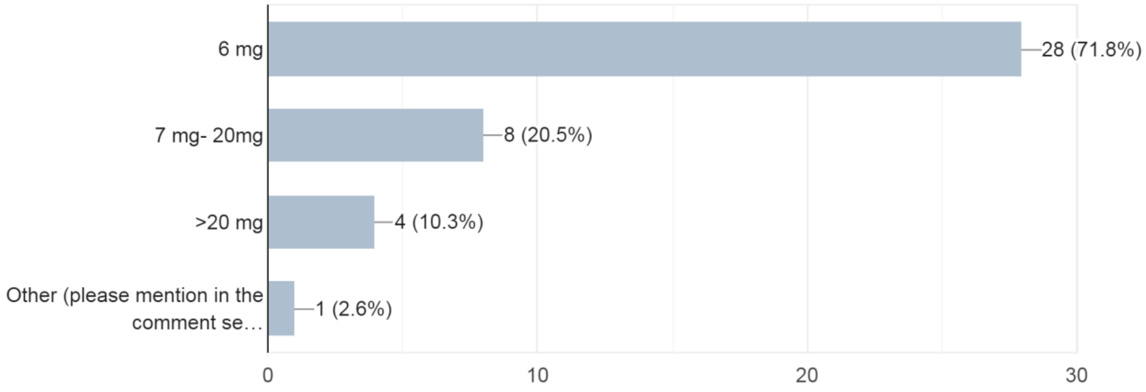

Comments:

1. We used 1 mg/kg of methylprednisolone
2. Methylprednisolone 1-2 mg/Kg/day
3. For ICU patients. Ward (moderate) patients receive 6
4. See above
5. Depend on condition, inflammatory markers, response to therapy
6. Depends on cases, we use higher dose and then de-escalation
7. Sometimes pulsed patients on ITU with Methyl Prednisolone 1g for 3 days then maintenance
8. Ampoules 8mg and so we generally give that dose. Some patients receive the evidence-based 6 mg dose.
9. According to the recent trials

20. What duration of corticosteroid use would you prefer for patients with C-ARF?

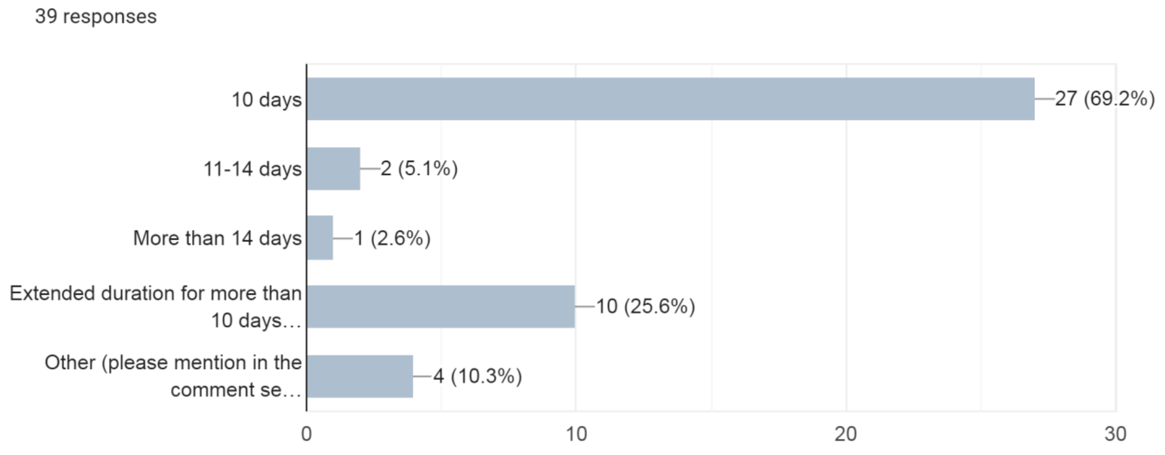

Comments:

1. But progressively reducing the dosage as mentioned above
2. Duration largely depends on clinical response, but the goal is to stop steroids at day 10
3. 6 days
4. See above
5. 5 to 7 days
6. In some patients we used less, in some more. we followed cytokine and clinical response
7. Dep on clinical radiological response
8. 7 days
9. Prolonged treatment increases the risk of secondary infections so caution; however if there is clinical or radiological evidence of ongoing "inflammation" the duration can be extended to 2 weeks
10. Suggestions obtained from the last trials

Section-2: Invasive Mechanical Ventilation

1. The following options may be considered as an appropriate trigger for tracheal intubation in C-ARF?

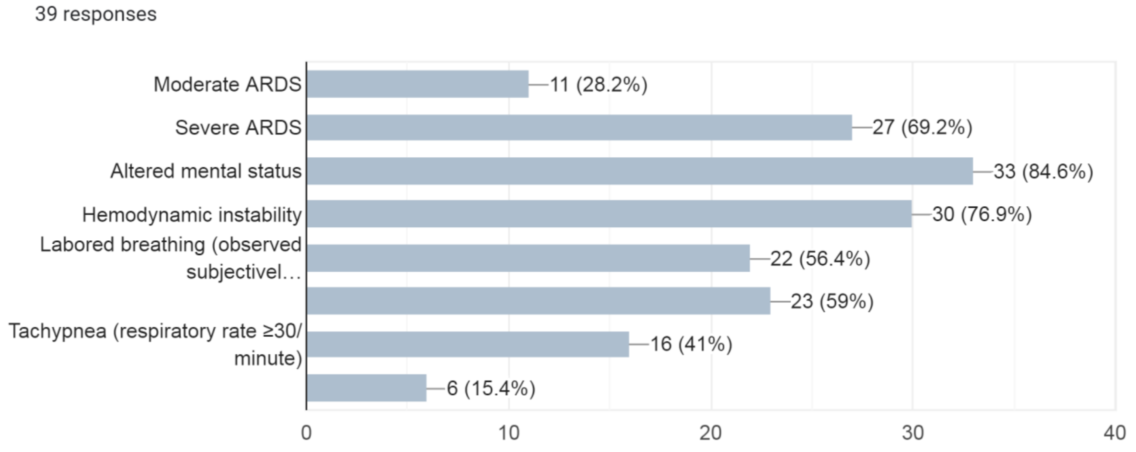

Comments:

1. Activation of accessory respiratory muscles - in case use of LUS for evaluation further at bedside - see also recent reports
2. Non cooperative patient, increasing CO2, increasingly frequent severe hypoxemic episodes (periodic saturation drops)
3. Moderate should be de-checked but cannot technically (bug in the survey?)
4. Dysponea increasing and a mixed the above
5. Trajectory matters (worsening/improving/not improving)
6. Severe hypoxemia with the use of high FIO2 SpO2 < 90% and/or persistent respiratory distress with subjective signs of increased work of breathing
7. We triggered on P/F and NIV-trial failure
8. Standard criteria - emergent, non-response to hfno, niv
9. Individualized according to each patient
10. Worsening chest X-ray or CT, clinical exhaustion, P-SILI, cardiac failure (right heart), fluid overload and capillary leak, high abdominal pressure
11. Prefer to delay intubation unless there is worsening of oxygenation on NIV (>70% FiO2 requirements) with dropping P/F ratios; delayed intubation can also worsen outcome (>90% FiO2 requirements on NIV persistently)
12. No comment
13. ARDS is usually diagnosed after intubation, The same indications for non-COVID patients

2. Early intubation is preferred in patients with C-ARF.

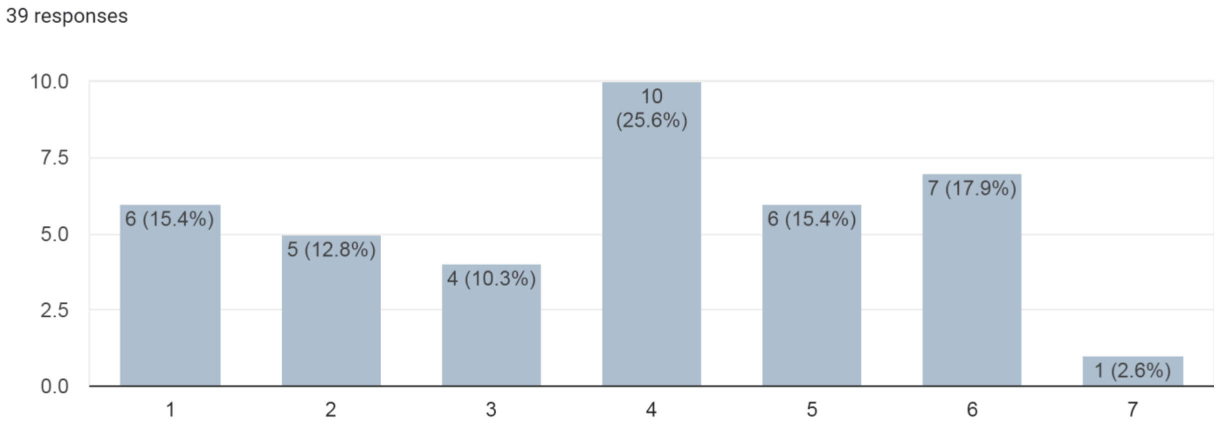

Comments:

1. When needed - see discussion about P-SILI and physiologic parameters above
2. I have no strong "gut feeling" about this yet. But I tend to postpone because intubation is prolonged anyway which increases complications. So the way I see it, less time with an ET tube is better.
3. No other rules than for other acute respiratory failure entities
4. We do not know
5. Not early intubation but also not very late. Same criteria as usually in ARDS patients
6. With. early I mean also "scheduled" rather than emergent (with deteriorating patients). we worked on some modified NEWS to identify at risk patients to "anticipate" intubation
7. We don't know - needs a RCT
8. Early this was the view, but that was due to inadequate non-invasive strategies being allowed
9. Do not allow the patient to become exhausted. Intubate prior to this if not settling or deteriorating despite appropriate interventions including HFO and NIV
10. It depends, in some patients with clear indication of course early intubation is better, but it cannot be generalized
11. Early intubation is to be discouraged since the outcomes of invasively ventilated patients is poor (>50% mortality) and those managed on NIV (even going on to invasive ventilation) tend to fare much better

12. Even though the data available do not show an increased mortality in patients receiving NIV and postponing the IM

3. The lung protective ventilation (LPV) strategy can be modified based on the lung compliance for C-ARF.

39 responses

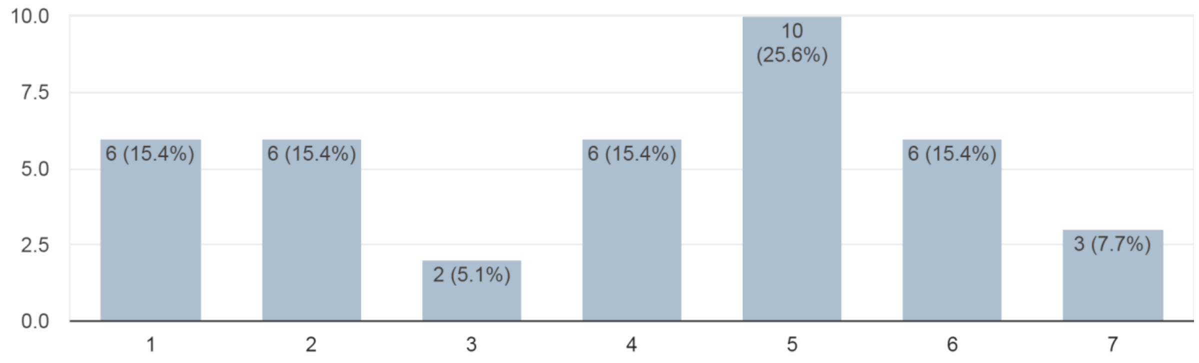

Comments:

1. NO - we must always use protective ventilation - in COVID since the beginning
2. In principle yes, however, I do not see a disadvantage of lung protective ventilation in COVID19 so we stay with this.
3. More compliant lungs can receive a bit more of tidal volume and less compliant lungs receive a bit less tidal volume than usual
4. Lung protective ventilation is applied by default independently from the compliance value
5. We always tried to keep, we were very conservative and protective with ventilation
6. We don't know - needs a RCT
7. Yes based on a n individual case review
8. Some patients are overdistended easily, so need to be careful
9. Instead of Gattinoni et al. suggesting 2 phenotypes based on LUNG characteristics (elastance, L-to-R shunt, weight, recruitability), I suggest to add other comorbidities (IAP, BMI, Fluid balance, BIA (VE), EVLWI, PVPI) - then you can combine both so there are 4 phenotypes LL-LH-HL-HH
10. It is important taking in consideration Driving Pressure, Pplat, PEEP and TV

4. A low PEEP strategy should be used during invasive mechanical ventilation of C-ARF

39 responses

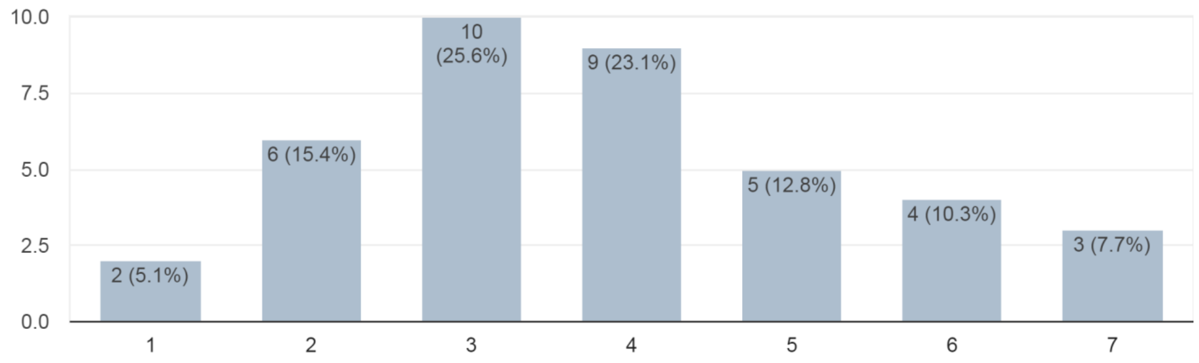

Comments:

1. Yes PEEP 8-12 cmH2O at max - in the majority of patients - of course a variability might exists - even if we found these patients very similar each other - and recruitability differences minor among patients - another factor to be considered is the time from symptoms - hospitalization and intubation - so time of the disease might affect pathophysiology (recruitability or not) as well as duration of MV - with possible site of infections (primary to the lung) or systemic - after 14 days of mechanical ventilation if not improving
2. Initially low PEEP but later should be titrated according to P/F ratio based on
3. This is not a yes/no question. Some patients have surprisingly good compliance and do not need high PEEP. I suspect these have a greater lung perfusion issue than a ventilation issue. Others have a greater component of recruitable lung and benefit from high PEEP (usually obese patients). The third category has poor compliance with and without PEEP and they will remain hypoxemic regardless. These probably have severe ventilation and perfusion issues. PEEP does nothing for them.
4. Low to moderate PEEP (5 to 8) as long as the lung compliance is not decreased
5. Depends on phenotype
6. Depends on compliance and recruitability
7. Depends on the severity
8. No
9. Depends on bedside trial/PEEP responsiveness taking into consideration respiratory and cardiovascular effects of PEEP
10. AT least a high PEEP strategy should be avoided as we have observed a high risk of creating overdistension. We do not use a low PEEP strategy but a "moderate PEEP strategy" with PEEP between 8 and 12 cmH2o titrated to have the best possible compliance
11. We based our PEEP titration on imaging, CT scan and mostly lung ultrasound
12. No - current state of Evidence is LPV as per ARDSNET Trials
13. In the non-dependent type - we had CT data on all of ours
14. Based on compliance and severity of hypoxia
15. We do not use very high levels of PEEP; assess each patient and determine what is suitable based on appropriate variables but have not used high PEEP
16. It depends on the time-course and phenotype and other comorbidities and/or complications as discussed above: (IAP, BMI, Fluid balance, BIA (VE), EVLWI, PVPI)
17. The PEEP mostly used has been close to 12

5. How would you select PEEP during invasive mechanical ventilation of C-ARF?

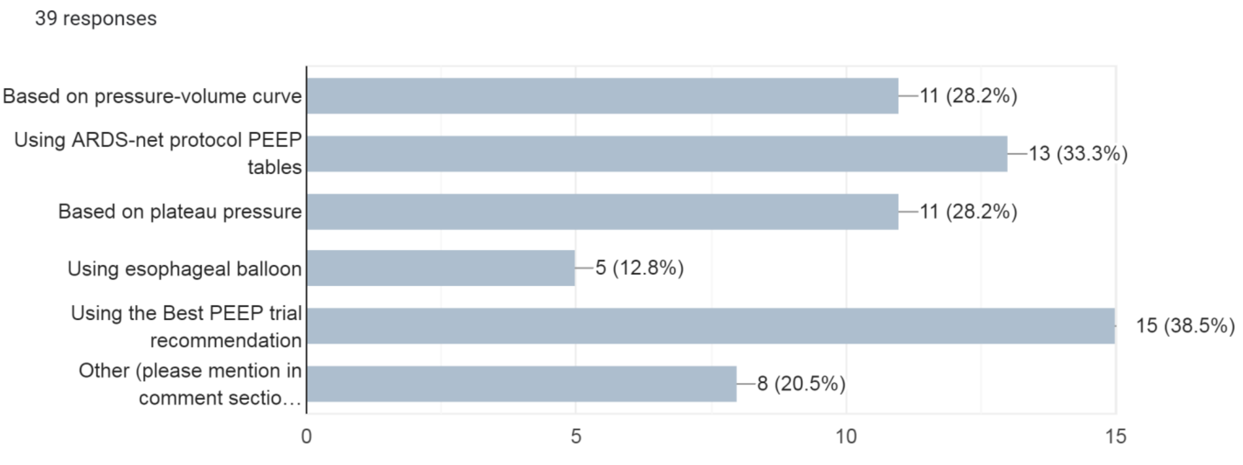

Comments:

1. By using low PEEP/FiO2 Table (in general PEEP between 8 to 12 cmH2O) - our studies showed that increasing PEEP at 16 cmH2O increased driving pressure in all of them ! if you use driving pressure - the risk is to set too high PEEP

2. I seek the point where Saturation is reasonable (>86%-88%) and the tidal volume is the highest with the lowest mean. It is never PEEP alone. It is always a balance between the driving pressure, the I:E ratio, the RR and the PEEP.
3. I titrate to driving pressure / dynamic compliance
4. Based on imaging, and balancing oxygenation and hemodynamics
5. Oesophageal balloon in morbidly obese, whom we see a lot in CV19
6. Assessing recruitment at the bedside together with gas exchange and hemodynamic response
7. Depends on bedside trial/PEEP responsiveness taking into consideration respiratory and cardiovascular effects of PEEP
8. Using P/F ratio and pressures
9. In order to have the best possible compliance
10. Ultrasound, BGA response, CT scan. if possible
11. Lowest PEEP at which oxygenation is acceptable (SpO2 >88% or paO2 > 8 kPa) with FiO2 up to 60%
12. Recruitment to inflation ratio or EIT and clinical tolerance
13. We target driving pressure = plateau minus PEEP < 14. again ARDS is a syndrome and NOT a disease, also it has a dynamic time course. We look at all parameters, lung characteristics PLUS other factors (IAP, BMI, Fluid balance, BIA (VE), EVLWI, PVPI) - also important is whether or not there is superinfection or fluid overload. We set ventilation and PEEP according to abdominal pressure. See https://pubmed.ncbi.nlm.nih.gov/31025221/ also in case of late C-ARF and potential for fibrosis (as you see destruction on CT) then we go for "close the lung and keep the lung closes" cf the squishy ball hypothesis (see https://pubmed.ncbi.nlm.nih.gov/32020548/)
14. The combination of the various techniques helps to individualize

6. Neuromuscular blockade is required during the early phase of invasive mechanical ventilation in C-ARF.

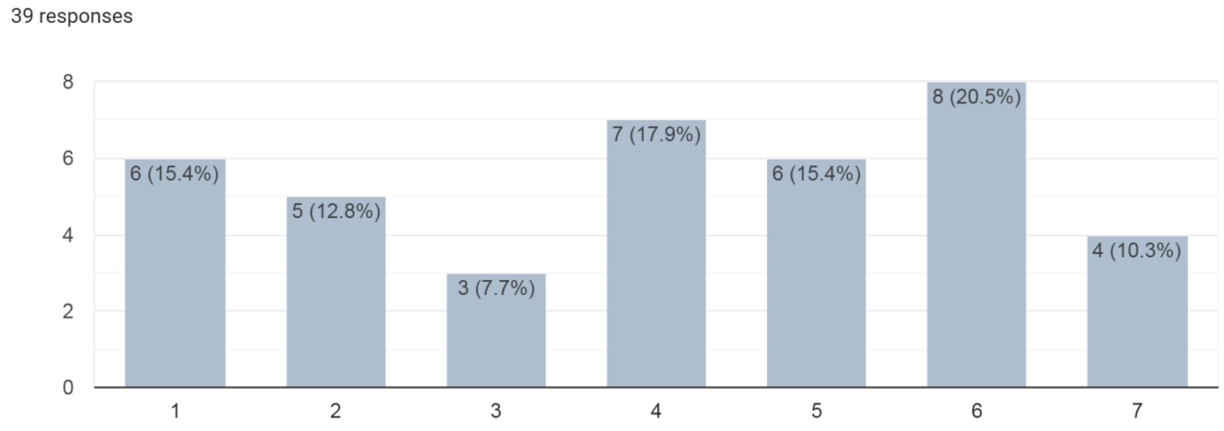

Comments:

1. In most of patients is required - but for a limited number of days - as less as possible - we started weaning as soon as possible according to the response of patients - limiting NMBA - due to the concomitant use of corticosteroids or in case other anti-inflammatory agents
2. The patients have a very powerful respiratory drive. It is needed to overcome it. Too much sedation causes a blood pressure drop which lowers the pulmonary perfusion pressures and can worsen hypoxemia
3. NMB initiation depends largely on severity of C-ARF
4. See above. It is a different pathophysiology of respiratory failure, spontaneous breathing normally works
5. Ventilator-patient synchrony is a key at the early stage of ARDS support
6. Depends on severity, will consider in severe ARDS
7. Maybe intermittent rather than continuous
8. Not always required. May be considered if clinically indicated (i.e persistent hypoxemia or inability to ventilate)

9. Usually yes in order to deliver strict protective ventilation
10. Shortly as you can, but needed
11. Usually to control the very high drive to breath and also high VR ratio to prevent P SILI
12. Selectively, in patients who cannot be managed with heavy sedation
13. If there are large respiratory swings in driving pressure then yes, NMB are indicated to avoid P-SILI
14. The use of neuromuscular blockade is dependent on the severity of hypoxia as well the synchrony between the patient and the ventilator
15. No more than 48h
16. It depends if mode-severe ARDS is present or not

7. It is harmful to allow spontaneous breathing during the first 72 hours of invasive mechanical ventilation.

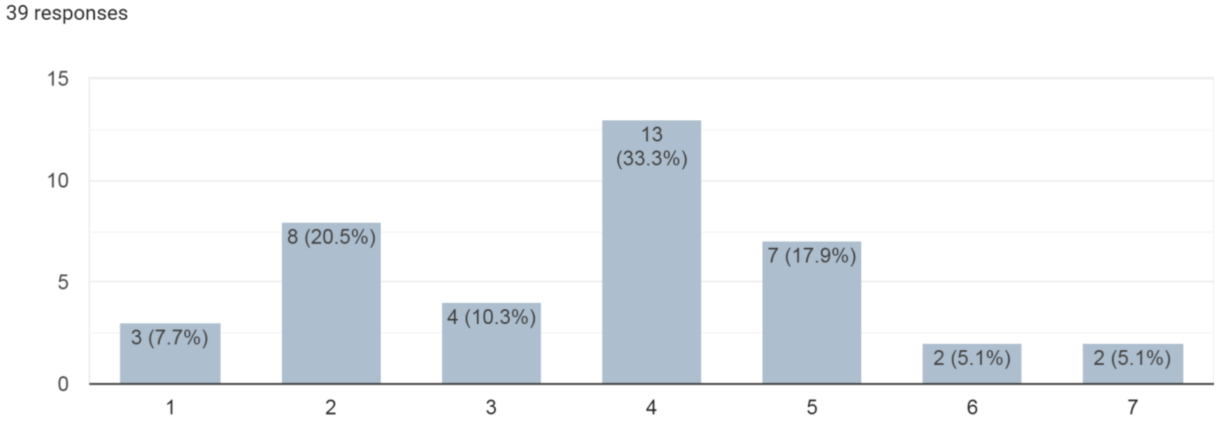

Comments:

1. I don't know if harmful or not - but since patients can be severely hypoxic - we started weaning after 2 - 3 days ... but certainly no after one week
2. No data but there seems to be a lot of breath stacking without
3. depending on severity of hypoxemia
4. It is harmful in some settings e.g. large VT or large transpulmonary pressure
5. Depends on severity of ARDS, will consider to avoid in severe ARDS avoid in
6. Depends on effort
7. Probably yes at least using pressure support ventilation as respiratory drive is usually very high and could be associated with very increased transpulmonary pressure.
8. Given the respiratory mechanics pattern in COVD-19 I might say yes (P-SILI)
9. Needs monitoring
10. We don't know - needs a RCT
11. In the more severe less compliant cases, but trial- if paradox breathing then stop
12. Rather yes because of P-SILI
13. It was never demonstrated to opposite

8. The invasive mechanical ventilation strategy in C-ARF should be targeted to the following?

39 responses

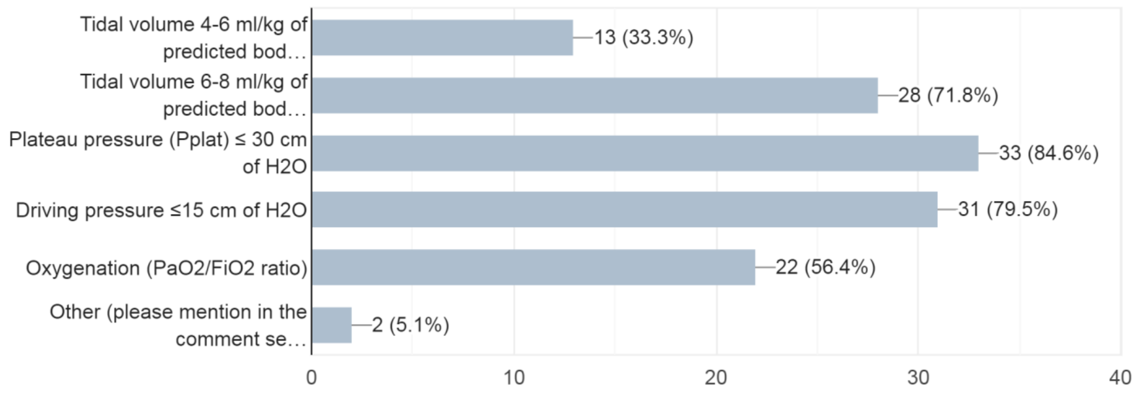

Comments:

1. Minimal peep for minimal oxygenation sat O2 88-91 - PaO2 55 - 85 - pH higher than 7.2 - 7.25 (which was almost never a problem in the majority of patients)
2. pH >7.25 if possible
3. Tidal volume selection largely depends on lung compliance and patient's severity of illness
4. Moderate PEEP and early prone position
5. A mix of the above
6. We learnt to "maintain" rather than chasing the best P/F, not to pay the due of aggressive ventilation. Just to buy time..
7. 6 ml/kg PBW
8. Look At VR trend too (Sinha, Singh, Fauvel, Soni BJA 2009)
9. We allow permissive hypoxia and permissive hypercapnia
10. As always it should be, a combination of those is better for individualizing

Section-3: Refractory Hypoxemia

1. Recruitment manoeuvres are efficacious in managing refractory hypoxemia with C-ARF.

39 responses

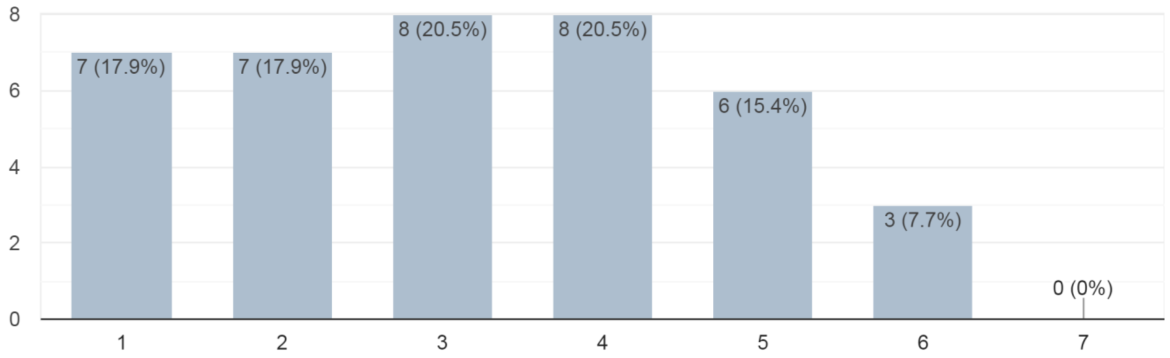

Comments:

1. Please use RM as less as possible in ARDS and specifically in COVID -19 patients - only as a rescue
2. Only in obese patients and perhaps several more that have atelectasis.

3. Increase PaO2 only by decrease CO (and increased risk of cardiac arrest, JAMA Brasilian Trial)
4. In my experience, it is very difficult to recruit these patients. I prefer putting them in a prone position.
5. Depends on lung damage pattern. we learnt to use ultrasound to distinguish recruiters and non-recruiters
6. We use them occasionally
7. Again yes and no - depends on case mix - check abdominal pressure and check comorbidities - perform cardiac ultrasound
8. Be careful for the potential hemodynamic influence

2. Prone position during invasive mechanical ventilation of C-ARF IMPROVES OXYGENATION.

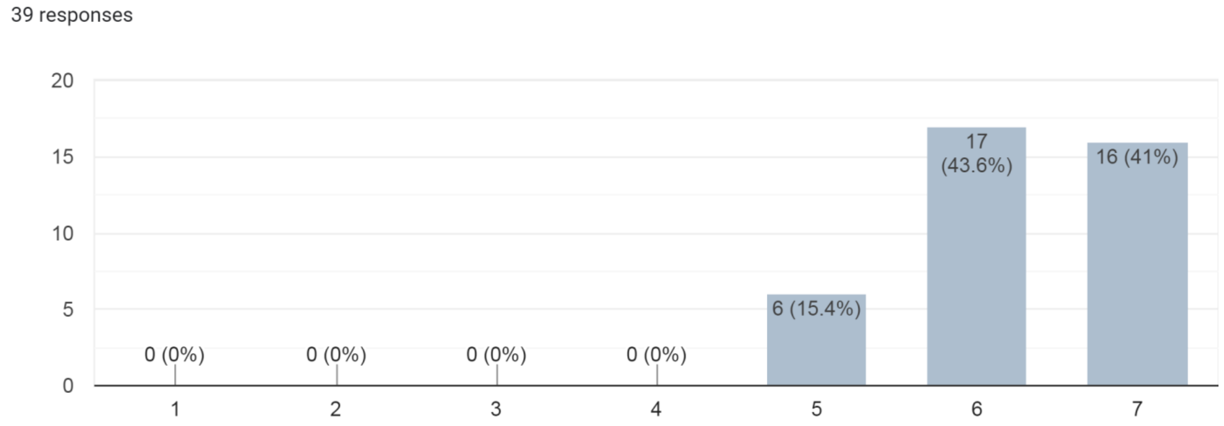

Comments:

1. We used in 30 per cent of patients - to be used cautiously - for reasons discussed above - response in oxygenation is mainly due to redistribution of perfusion and often associated with increased PaCO2 and reduced pH (with increase in driving pressures) - this might promote Bohr effect - thus for lower oxygenation low saturation than expected - thus if we decide to use minimal oxygenation with minimal PEEP (due to poor recruitability) we should avoid decreases in Ph - to avoid Bohr effects on saturation - further remember that prone - especially if prolonged in time - is associated with collateral effects - and sometimes contraindications - further difficult to manage many proned patients with not adequately trained health care professionals - which may occur during emergencies - finally the risk of adverse effects during proning
2. Early disease/intubation yes. Late disease/intubation less.
3. Very efficient strategy in my experience
4. Yes, but may be just cosmetic. We noticed that responders to RM may have better responses to pronation. our proning rate diminished in late phase of pandemic
5. Supported by RCTs
6. We almost saw improvement in early stages
7. There are however non responders

3. Prone position during invasive mechanical ventilation IMPROVES SURVIVAL in C-ARF.

39 responses

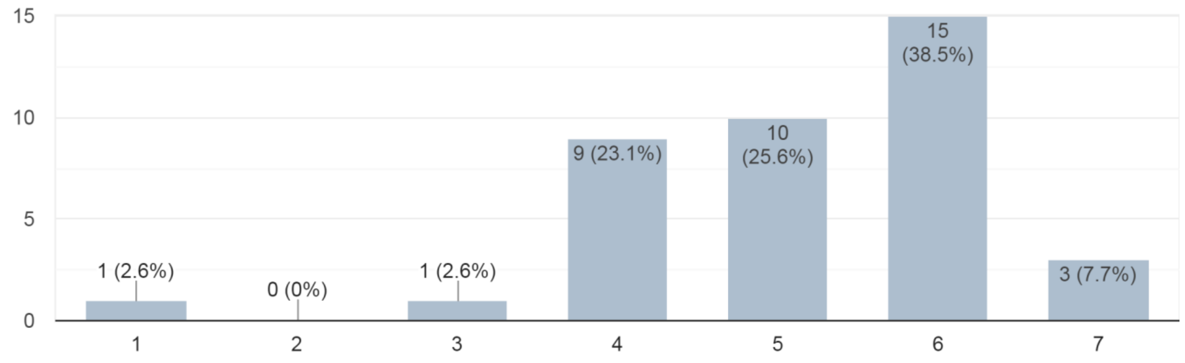

Comments:

1. See also my comments above on organization problems of proning as well as contraindications and potential collateral effects - We know is effective in traditional ARDS - we don't have clear data in COVID
2. Not sure
3. No randomized trial, but it is likely
4. Depending on the severity of ARDS
5. It improves survival in ARDS but data regarding survival benefit in C-ARF are lacking
6. Don’t know any data but suppose translation of non-CV19 data in moderate to severe ARDS
7. Where is the data?
8. Probably yes as it is the case in other ARDS. However, in the absence of prospective randomized data, it is difficult to be affirmative.
9. We need data
10. We don’t know - needs a RCT
11. Unclear
12. Not sure too little data
13. No comment

4. Prone position during invasive mechanical ventilation of C-ARF is helpful when done for (duration per session)?

39 responses

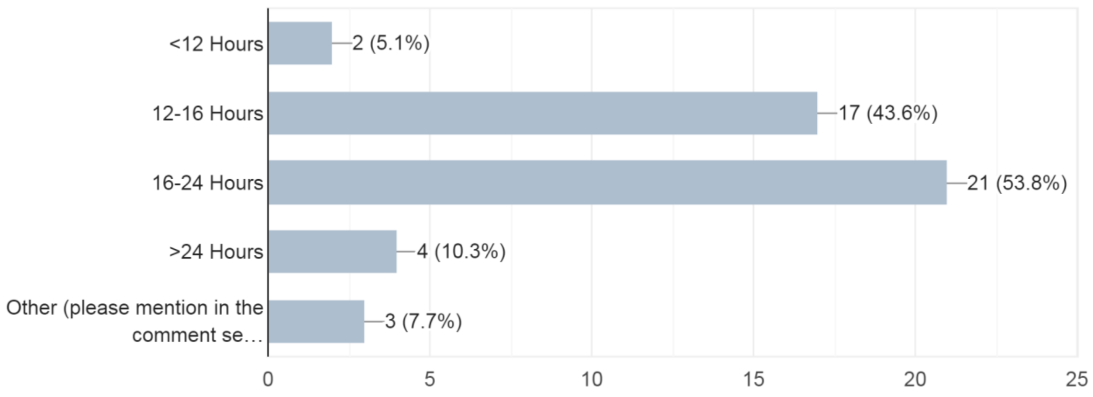

Comments:

1. This is a good compromise - we have no data if longer period of time are feasible and helpful - anyway please consider my comments above about pathophysiology of proning in COVID
2. I don't know as there is no data guiding C-ARF proning duration. I would generally use a duration <12 hours
3. Possibly as early as possible, but where is the data?
4. We use 16 to 18 hours sessions and sometimes longer sessions in the most hypoxemic patients.
5. We dont know - needs a RCT
6. PROSEVA
7. Unknown. tend to use 16h
8. We have left patient for longer duration in prone with good result and minimal drawbacks (facial decubitus)
9. The available evidence suggests >16 H

5. Advanced mechanical ventilation modes may be BENEFICIAL in refractory hypoxemia with C-ARF.

39 responses

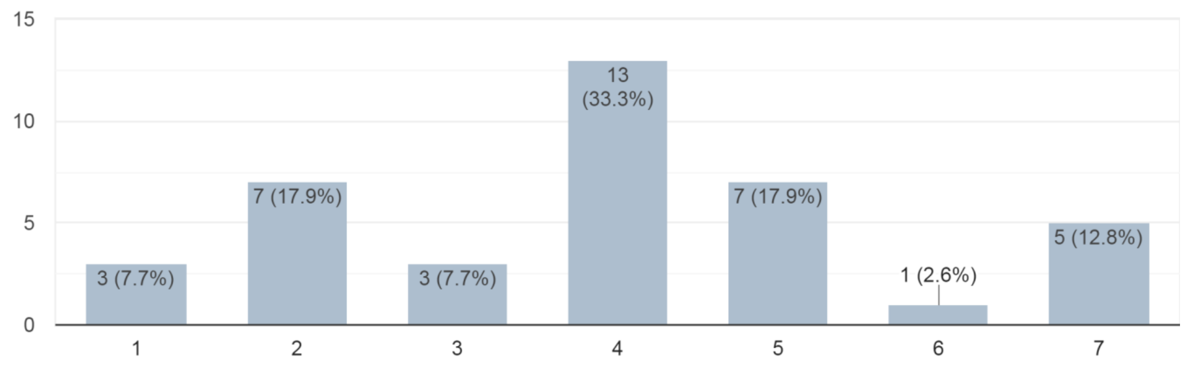

Comments:

1. No - to be as easy and simple as possible - considering also organiative local situations in emergency - remember the organizational aspects in emergency - and need for training of health care professionals
2. Only pressure control ventilation or APRV
3. I don't know what 'advanced mechanical ventilation modes' refers to
4. What does this mean?
5. APRV maybe but without strong scientific background (French BIRDS trial not yet published)
6. Where is the data?
7. Difficult to manage with so many patients to treat. However, in my experience BIPAP APRV with conventional I:E ratio could be of some interest in specific patients.
8. The more protective and tailored
9. I don't know what this means (Advanced mechanical ventilation modes)
10. We don't know - needs a RCT
11. We have used quite a lot of APRV and found it to be useful
12. We saw good effect of APRV
13. It depends on patients

6. The following ADJUVANT therapies are helpful in refractory hypoxemia with C-ARF?

39 responses

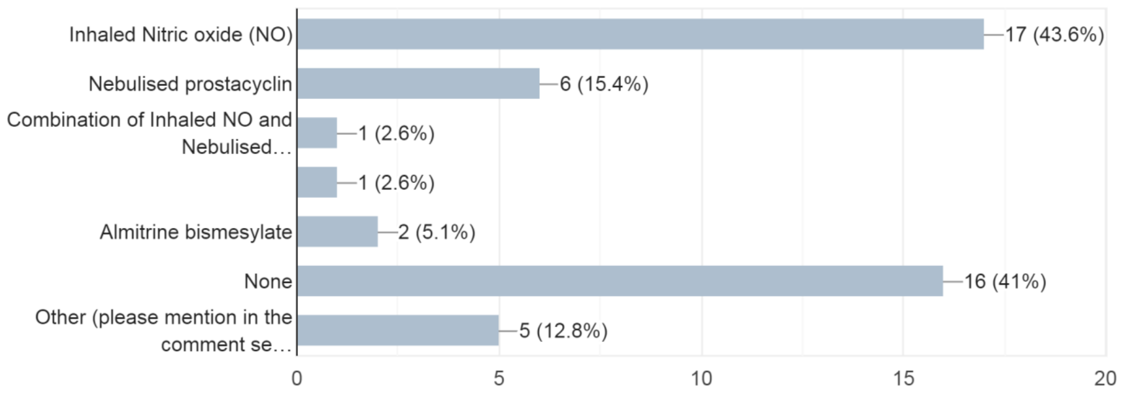

Comments:

1. We used NO with promising results - the limitation was the number of machine available - we are matching CT scan with NO response - mainly again on regional perfusion effects - I believe NO might be effective to buy time is particular hypoxic conditions
2. I largely stopped routinely using inhaled NO due to cost and lack of clear clinical benefits and not just improved oxygenation
3. Only for severe hypoxemic period, not controlled with usual ventilatory support
4. Unless RV failure where inhaled vasodilators can help
5. No comment due to no personal experience
6. We noticed some changes after systematic use of (titrated) anticoagulation
7. Sildenafil also in severe cases with RV dysfunction
8. Prone positioning, NMB, PC-IRV
9. We have not routinely used the options above
10. We also used IV milrinone in case of right heart failure as well as judicious use of IV fluids and de-resuscitation (see https://pubmed.ncbi.nlm.nih.gov/32449147/, https://pubmed.ncbi.nlm.nih.gov/29789983/ , https://pubmed.ncbi.nlm.nih.gov/32337020/ , https://pubmed.ncbi.nlm.nih.gov/31399779/ , https://pubmed.ncbi.nlm.nih.gov/25432556/)
11. Not sure but adjuvants can be tried (e.g. inhaled NO), particularly if no deleterious effects noted
12. The use of these adjuvant therapies is extremely rare
13. It depends what we mean by helpful, improved oxygenation vs improved outcomes
14. If evidence of pulmonary hypertension

7. Veno-Venous Extracorporeal membrane oxygenation (V-V ECMO) may be considered in C-ARF patients on invasive mechanical ventilation?

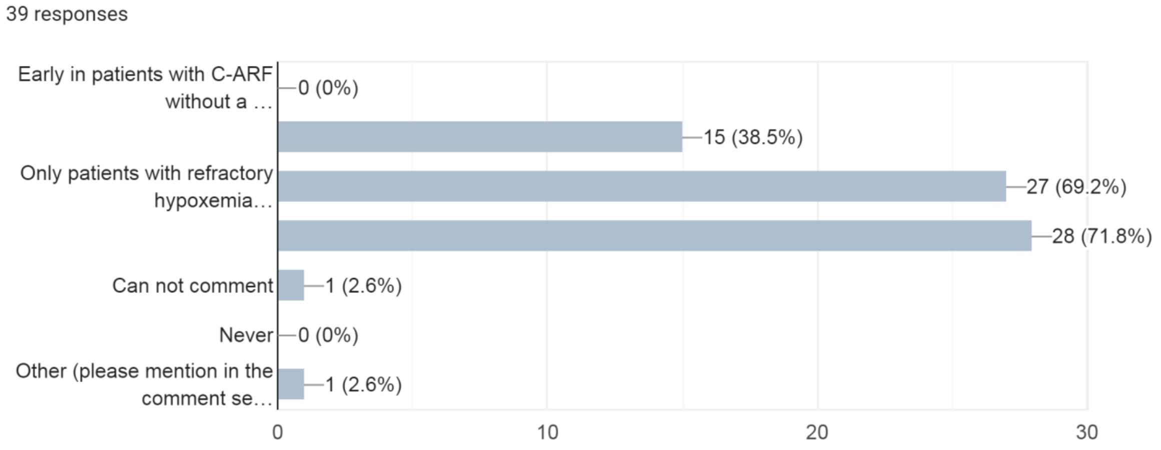

Comments:

1. We had not good experience with ECMO ... in late stage no effect - just prolonging agony of patients - difficult to decide when disconnect - very long time of applications - if early difficult to organize in emergency situations with many patients admitted to ICU - not clear clinical effects - be very cautious - I am not very much in favour of ECMO in COVID
2. ECMO experience is a key condition
3. Refractory Hypoxemia despite Proning
4. depends on resources, contraindications
5. Sometimes used in our unit in refractory hypoxemia with no response to prone positioning in the younger patients with good results.
6. We used it very rarely.
7. We had national criteria based on modified Respiratory score
8. Would try to prone first if possible, but sometimes reasons why can't
9. When we did not yet reach the top of the first wave curve we did not put patients on VV ECMO where we normally would do. As our knowledge progresses I believe that now early VV ECMO in some awake patients could be useful
10. The use of VV ECMO is dedicated to specific cases of COVID 19

Section-4: Infection Control

1. The following are considered as aerosol-generating procedures (AGPs)?

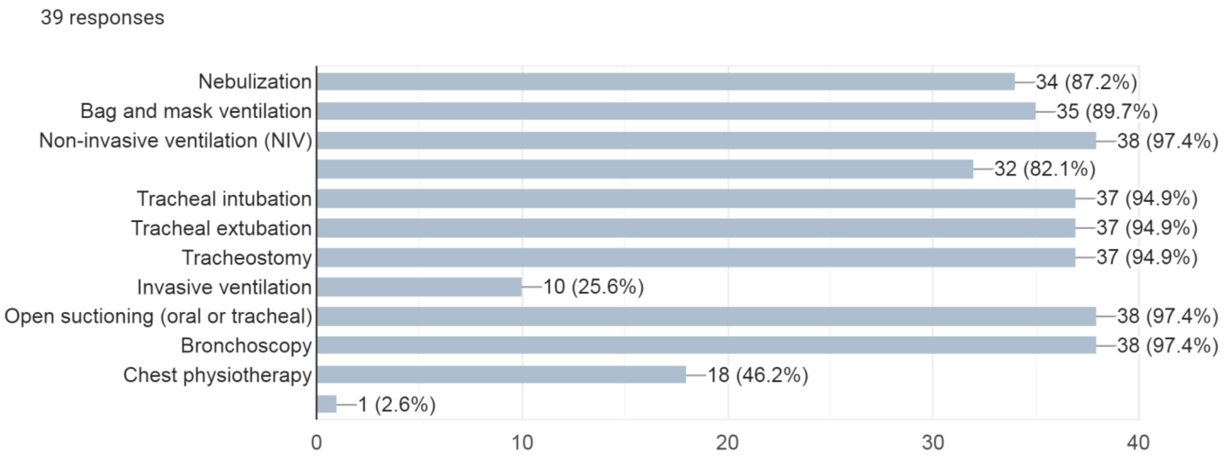

Comments:

1. We tried to have during specific maneuvers patient in specific room fully aerated - but the most important to have adequate dressing and protection for health care professional team - we had no contamination among us - in three months hard work on COVID in ICU and ward
2. Invasive ventilation and physiotherapy do not require opening the tubing
3. If the preventive measures (FFP2 masking, etc.) have been followed the risk for staff infection is low
4. Physiotherapy under invasive ventilation ?
5. Invasive ventilation: if uncuffed ETT or disconnection. we also consider FONA, use of SADs, awake intubation
6. I developed a safe protocol for bronchoscopy
7. Institutional protocol is for N95/PAPR, negative pressure rooms for COVID (+) patients
8. Invasive ventilation should have the same precautions because of disconnection risk.
9. These were our official high risk procedures but I believe all the ones above can be considered as such • Intubation (best with glidescope), endotracheal suction • Administration aerosol • Bronchoscopy • Physiotherapy • Bedding • Proning or change of position • Gavage insertion and other actions that may induce coughing or sneezing • HFNO - high flow nasal oxygen (Optiflow) and NIV with a mouth and nose mask
10. All those may produce aerosols in some circumstances

2. High flow nasal oxygen (HFNO) is considered less aerosol generating compared to non-invasive ventilation (NIV).

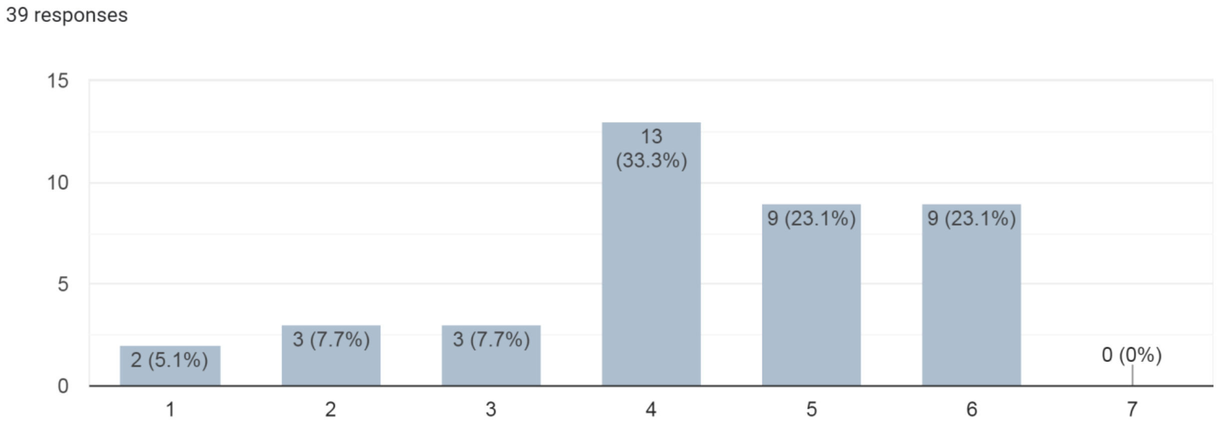

Comments:

1. Difficult answer - some data suggest that - but we must be careful in any case – protection, protection and protection
2. There are three comparative studies. All in models. Basically not much difference.
3. It depends on how circuit is settle
4. Probably yes but not definitely proven
5. we need data for viral particles, and it also depends on patient's viral load
6. My reading of evidence suggests so
7. We place a surgical mask over the interface with HFNO and ensure most optimal seal with NIV masks to minimize potential aerosolization
8. It depends. Using surgical masks reduces aerosol generation with HFNO. The use of NIV through a single-limb machine is associated with high aerosol generation in contrast to double limb machines.
9. It is always considered as such but personally I am not convinced. HFNO runs at a flow of 50L and is an open system... vs NIV with special facemask and filter is a semi-closed system
10. If the NIV is delivered through an Helmet with HEPA filters the risk is certainly lower

3. The following measures may be considered in the ICU for infection control?

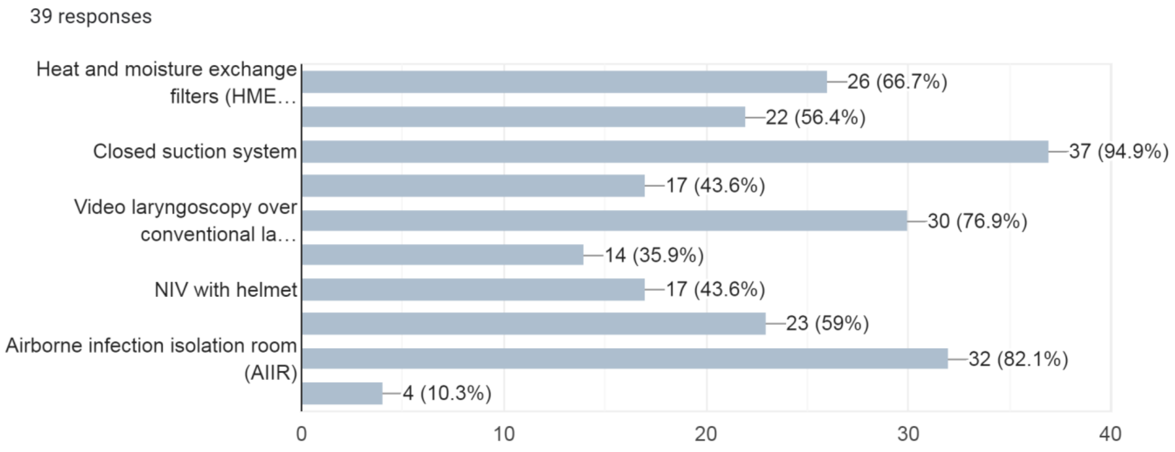

Comments:

1. All these checked might be helpful and used according the local availabilities and organization
2. Our COVID patients are not in the hospital structure. We constructed outdoor tents with negative pressure to protect the environment and are wearing top to toe PPE.
3. HMEFs May be effective by itself but require +/- daily changes, i.e. opening of the circuit with respective decontamination of the surroundings.
4. Only study dedicated to intubation did not find association between VL use and lower risk of COVID19 (Anaesthesia)
5. Not sure you refer to infections in general or sars-cov2 transmission specifically
6. We published a review discouraging aerosol boxes **(Text deleted for anonymity)**
7. SDD (selective digestive decontamination)
8. Bronchoscopy protocol - with inspiratory pause prior to circuit disconnect
9. We use HEPA filter in isolation rooms
10. Ideally the AIIR are very useful but scarce

4. Which personal protective equipment (PPE) is acceptable for use during an AGP in ICU?

39 responses

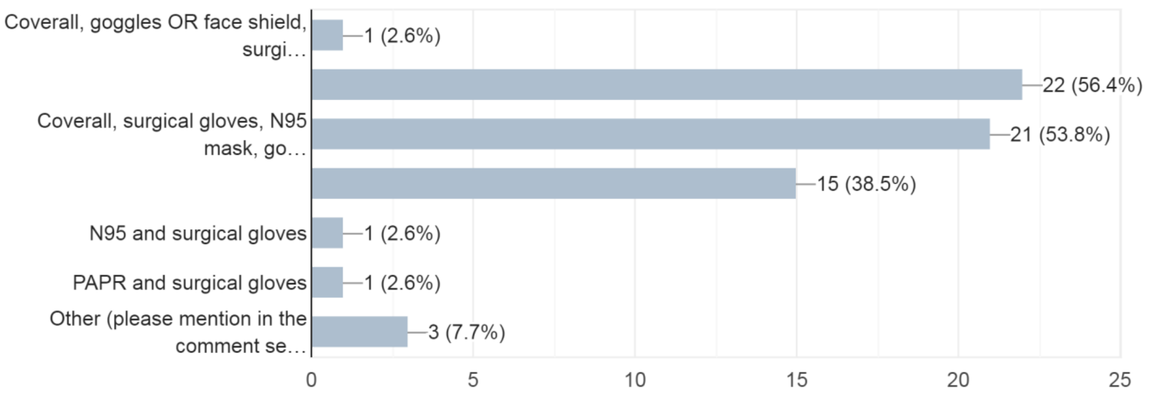

Comments:

1. We used gowns - coverall - N96 plus surgical mask above - gloves - decontamination - other technique we tried too complicate in clinical practice and difficult to have in adequate number if many patients to treat in the hospital and ICU
2. We use double gloving, a hair cap underneath the coverall hood and shoe covers too
3. Coverall, N95 with bonnet, without gloves (only alcohol hand hygiene)
4. hazmat suit, PAPR or FFP3, goggles, face shield beneficial, double gloves, oveshoes
5. Question would have been easier to answer as a multi select for each type of PPE
6. As some patients were in the same zone (no isolation box) we used cluster nursing care. Therefore whenever there was a AGP the nurse or doctor put an extra set of coverall and gloves and after patient contact all the gloves were removed for handwashing to avoid cross-infection
7. It depends on the resources availability

Section-5: Weaning and Tracheostomy

1. Which weaning strategy is best suitable for C-ARF patients on invasive mechanical ventilation?

39 responses

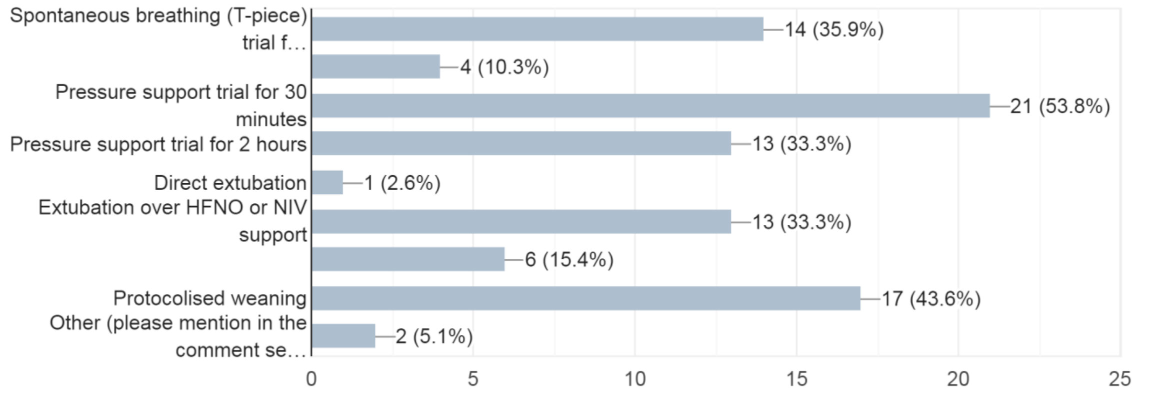

Comments:

1. Paper on Physiotherapy and weaning on respir physio neurobiol 2020 - **(Text deleted for anonymity)**
2. In patients ventilated longer than 7 days we use tracheostomy due to the act that extubation failure is not seldom and acute reintubation increases risks for the patient and the staff

3. The question is regarding 'weaning' but the responses relate to SBT and extubation. therefore, i'm not sure how to answer this question now.
4. Trajectory matters; has the patient improved from the time of intubation
5. Same strategy as usual
6. We wean according to patient tolerance and objective variables and will assess the readiness for extubation on a number of parameters
7. In case of presence of tracheostomy T-piece trials were prolonged
8. These are procedures most commonly used at my institution adapting to COVID 19 the protocoled weaning

2. Chest physiotherapy is beneficial in patients on respiratory support for C-ARF.

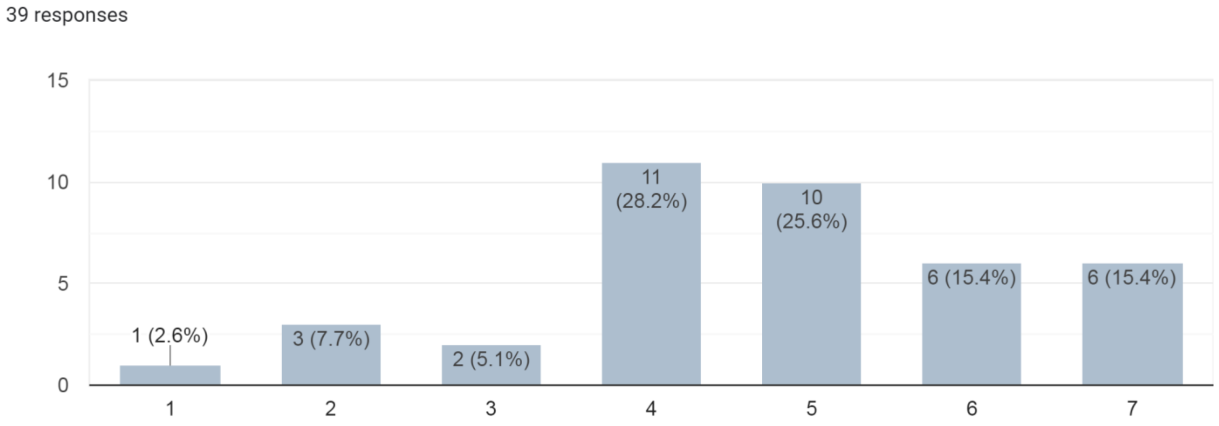

Comments:

1. Absolutely mandatory - we had a great effort in physio in COVID - during passive ventilation but more important during weaning and extubation phase - as well as early after extubation - we also organize d a special sub intensive care - next to larger ICU - where we admitted all patients extubated from our three ICUS - and keep the patients there for 3 to 10 days before discharge to the infectious disease ward - we did not observed any death - in hospital after ICU discharge - only one patient due to reason cardiothoracic not related to COVID
2. Only during weaning.
3. Mobilization maybe if you consider that a form of chest physiotherapy
4. In our experience this patients do not have important secretions
5. Physiotherapist need to be well trained and protected
6. I believe it is of paramount importance however our physiotherapists were reluctant/afraid because of AGP
7. Reasonable, but without evidence

3. Early mobilization of patients is BENEFICIAL in patients on respiratory support for C-ARF.

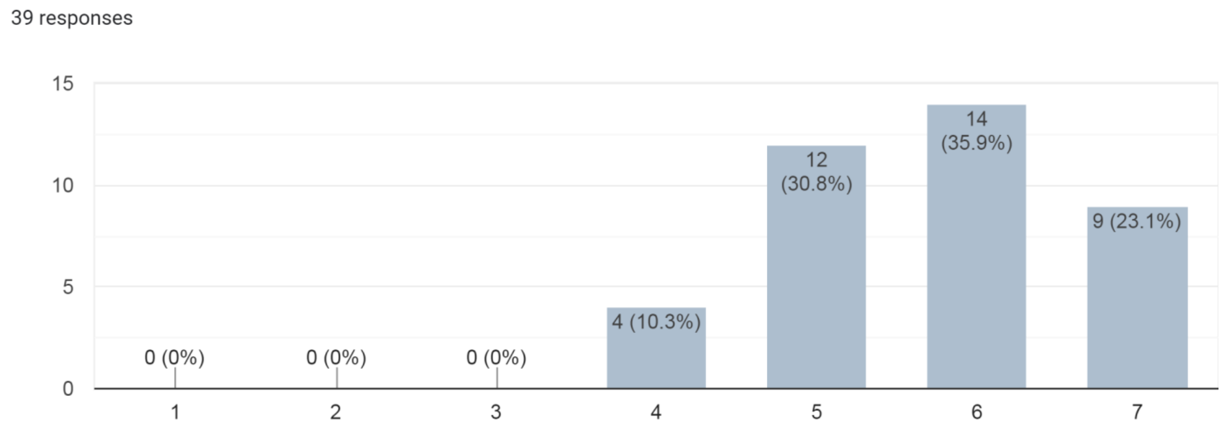

Comments:

1. Absolutely yes - physio is essential in covid patients and must be carefully organized **(Text deleted for anonymity)**
2. This is not specific to C-ARF, but for all pts
3. As tolerated
4. While it may be beneficial, it may be logistically difficult to institute.
5. No data available. Performed as in other RADS patients in our unit.
6. We tend to mobilize early
7. No comment

4. Delayed weaning (≥ 7 days) from invasive mechanical ventilation has HIGHER SUCCESS in patients with C-ARF.

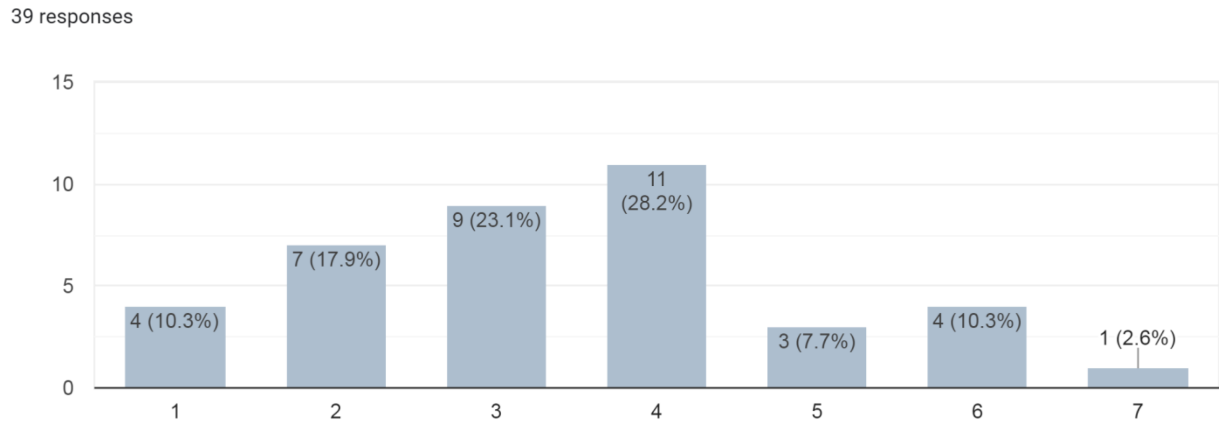

Comments:

1. It depends what do we mean as weaning - if weaning is assisted breathing , NO - so we must start assisted breathing and no NMBA as soon as the patient allow that - if we intend weaning process for extubation - yes - usually these patients are extubated one week later than normal ARDS patients - see time schedule on our paper on LRM
2. They do so poorly initially and take so much time to improve that early weaning is usually out of the question

3. We don't know; the course is generally longer but we don't have evidence that delaying weaning is beneficial compared to others
4. Depends on each patient evolution.
5. I may say yes, but once again for data we have, we still go for a tailored approach
6. We don't know - needs a RCT
7. Unclear what question is asking
8. Sounds counterintuitive but you need to wait for the cytokine storm to rest
9. COVID 19 patients can be at times weaned after 2 weeks of IMV

5. Based on your experience, do you believe that additional steroids are BENEFICIAL for tracheal extubation of a patient with C-ARF.

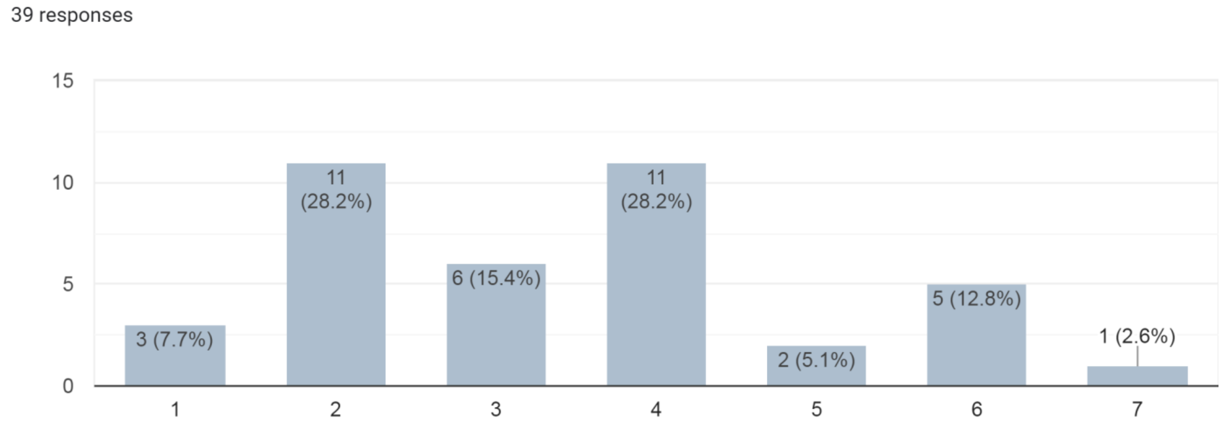

Comments:

1. They might be helpful and we used according to clinical status and leak test - see paper on respir physiol neurobiol 2020 **(Text deleted for anonymity)**
2. Nope
3. To a large extent, depends on how long the patient has been intubated
4. Any evidence for postextubation laryngeal oedema and CTC for COVID19 patients
5. Only for concern for upper airway edema (absent cuff leak etc)
6. Not useful in my experience but we have have very few extubation failure
7. Maybe once we identify some phenotypes addressing this choice
8. Don't know
9. If edema or cuff leak present
10. Only in rare cases
11. Maybe but this specific issue has not been sufficiently investigated
12. Seen quite a high rate of laryngeal oedema which caused airway issues/re-intubation/tracheostomy. Be suspicious, do leak test or look with FNE

6. When should tracheostomy be considered to facilitate weaning from invasive mechanical ventilation?

39 responses

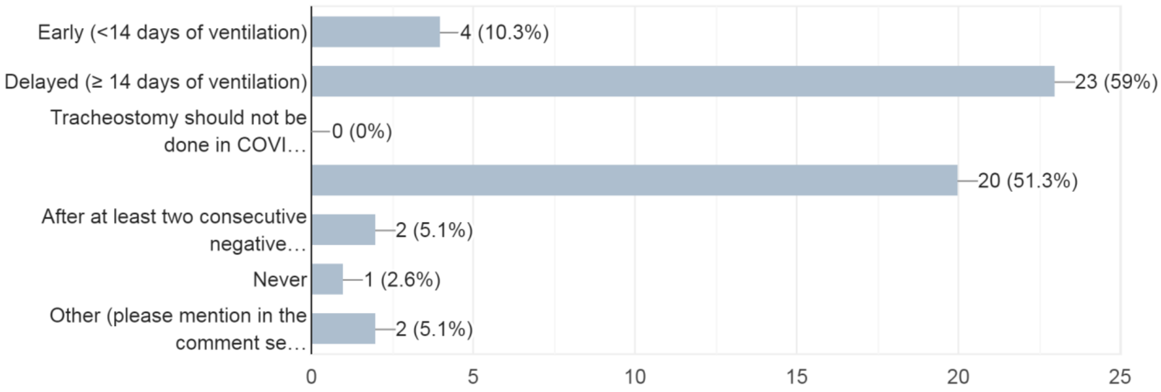

Comments:

1. Delayed - after 15 days - to understand if the patient can be extubated or not earlier - remember that in COVID - duration of MV is usually one week more than in traditional ARDS - thus since we consider an average time for Trach between 12 to 16 day in traditional ARDS - in COVID I would consider 19- 24 days - that is what we did and recommended/ discussed in our paper **(Text deleted for anonymity)**
2. Total clinical profile including radiology should be considered for decision for tracheostomy, If intensivist decides, based on clinic radiological data that extubation is unlikely in next 3-7 days, tracheostomy should be done
3. See above
4. Apart from intensivist view this will depend on ENT service opinion, in case this is the only service which performs TT (vs percutaneous)
5. In lower resources settings (particularly with regards to nurses and respiratory therapists)
6. Major concern about the risk of virus aerosolization. For this reason we use a delayed strategy. Overall, few patients have requires a tracheostomy
7. I don't see a benefit in tracheostomy for weaning, if not conventional indication. i am for postponing either for AGP-risks and also to avoid a tracheostomized patients overload, which would be difficult to address after ICU discharge
8. Based on patient requirements and progress. We have performed earlier tracheostomies where airway compromise may be a problem - e.g. in very obese patients and found this to be very helpful
9. The problem was that no-one wanted a weaned COVID patient with tracheostomy on the ward afterwards. At the end we declared every-one COVID negative after 4 weeks of ICU stay. Some were tested again and most were negative however some remained positive on PCR even after 4 weeks but were no longer considered to be infectious. It is a wicked disease
10. see previous comment

7. Which of the following technique of performing a tracheostomy is recommended for COVID-19 patients?

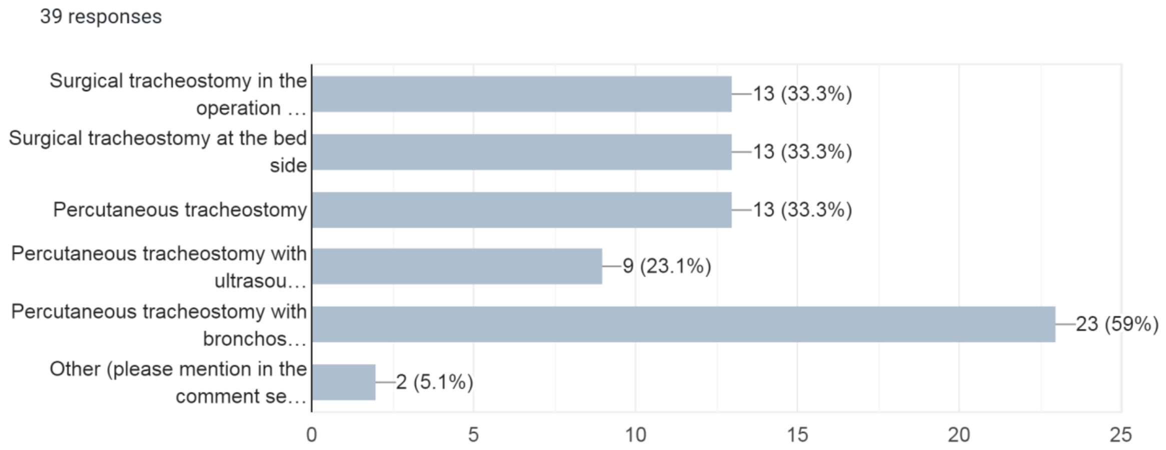

Comments:

1. We performed most percutaneous - of course under protection when possible in aerated room - and with ultrasound guidance as well as fibre optic guidance - **(Text deleted for anonymity)**
2. It also depends on local expertise
3. I do not know
4. Depends on availability
5. This is our strategy depending on the patients but I have no data to clearly support this.
6. We use surgical in (highly) selected cases, given the expertise with PT
7. the one making the team comfortable
8. As per local policy and experience
9. We use apneic periods with discontinuation of ventilation to minimise AGP
10. Various technique can be adopted minimizing the aerosolization moments
