# Supplementary material for: Expert consensus statements for the management of COVID-19-related acute respiratory failure using a Delphi method
Source: Crit Care. 2021 Mar 16;25:106. doi: 10.1186/s13054-021-03491-y (PMC7962430; doi:10.1186/s13054-021-03491-y)

Report of Survey Questionnaire Two

Section-1: Non-invasive Respiratory Interventions

1. The pathophysiology of COVID-19 related acute respiratory failure (C-ARF) is similar to that of acute respiratory distress syndrome (ARDS).

39 responses

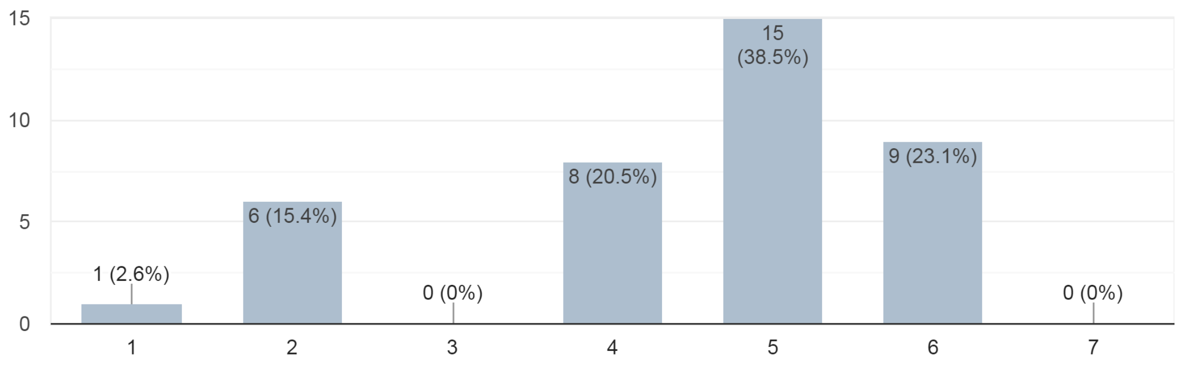

2. Based on your experience, awake self proning MAY IMPROVE OXYGENATION in patients with C-ARF.

39 responses

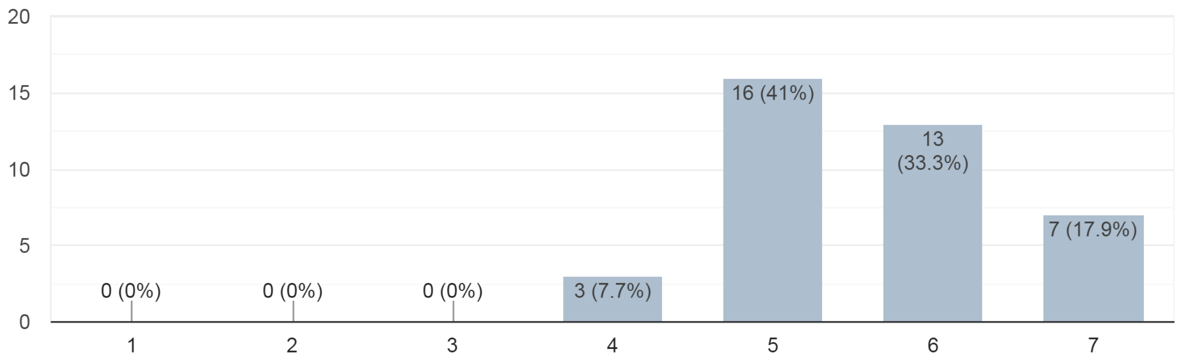

3. Based on your experience, awake self proning MAY prevent the need for INVASIVE MECHANICAL VENTILATION in patients with C-ARF.

39 responses

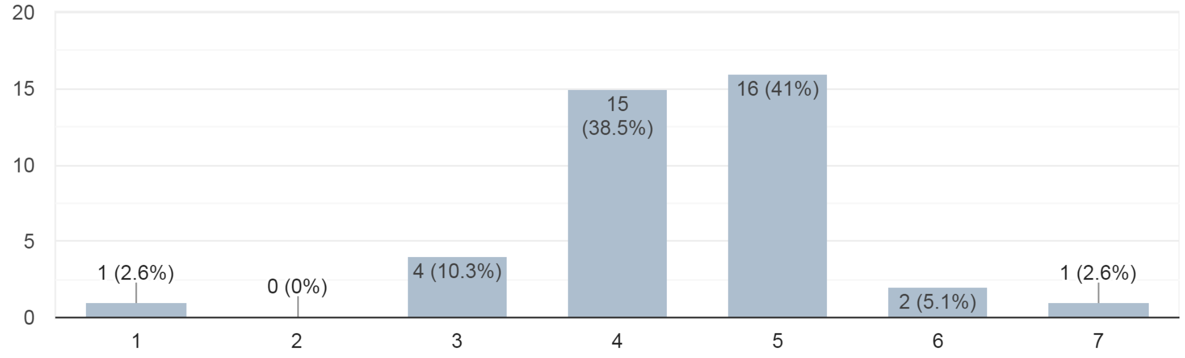

4. In which of the following clinical scenarios should awake self proning be initiated in patients with C-ARF?

39 responses

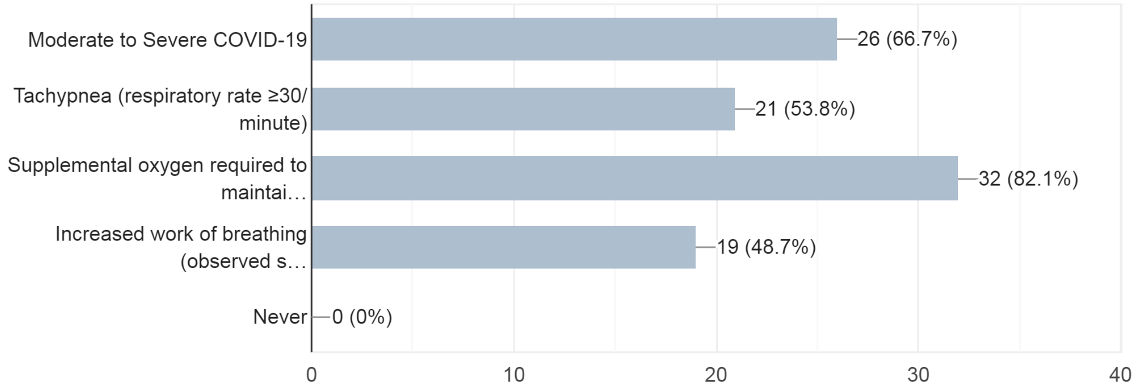

5. High flow nasal oxygen (HFNO) can be considered as an ALTERNATIVE STRATEGY for oxygen support before invasive mechanical ventilation.

39 responses

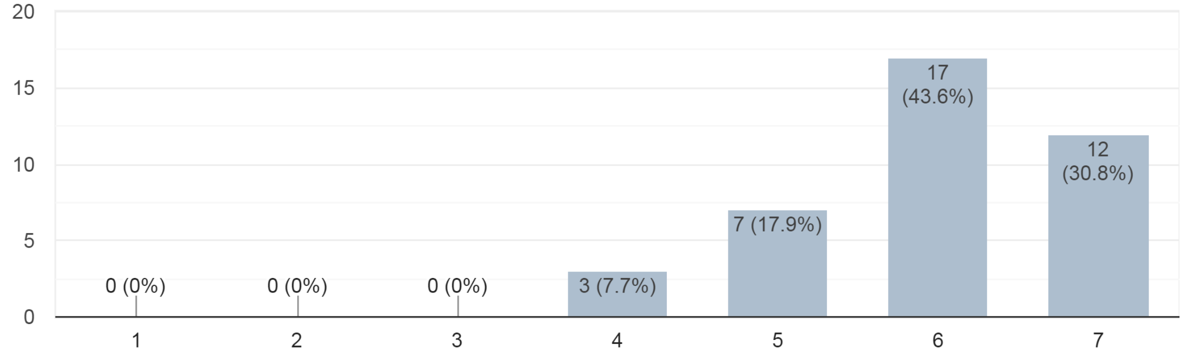

6. When do you initiate HFNO in patients with C-ARF?

39 responses

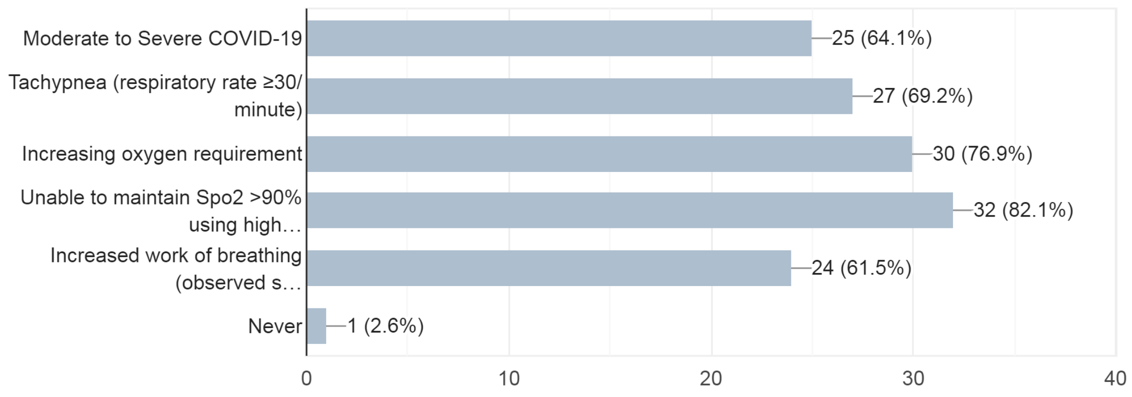

7. Based on your experience, HFNO may avoid the need for tracheal intubation and INVASIVE MECHANICAL VENTILATION in patients with C-ARF.

39 responses

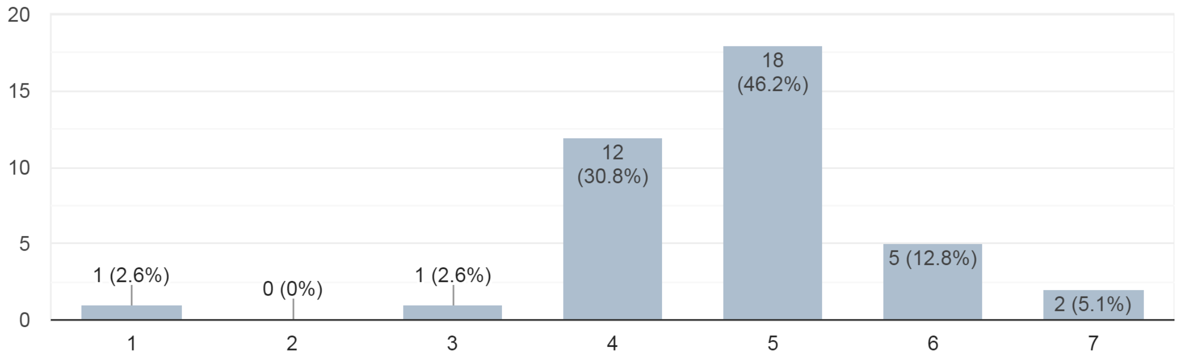

8. Non-invasive ventilation (NIV) can be considered as an ALTERNATIVE STRATEGY for oxygen support before invasive mechanical ventilation.

39 responses

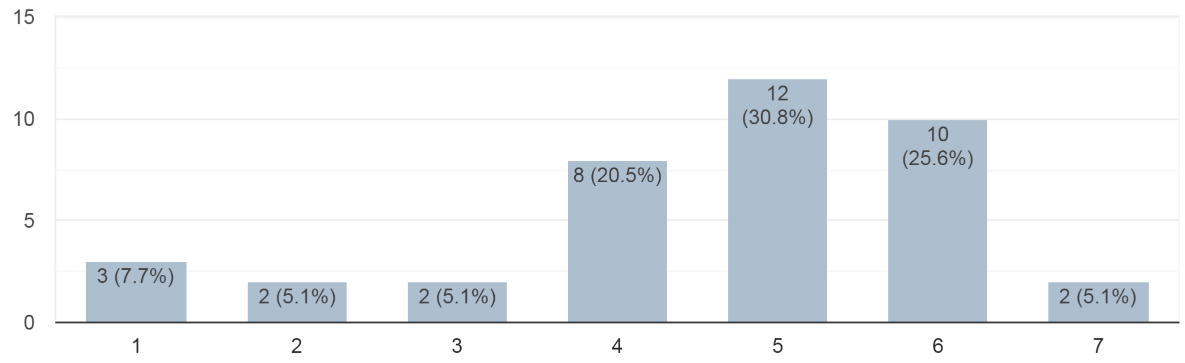

9. In which of the following clinical scenario should NIV be initiated in patients with C-ARF?

39 responses

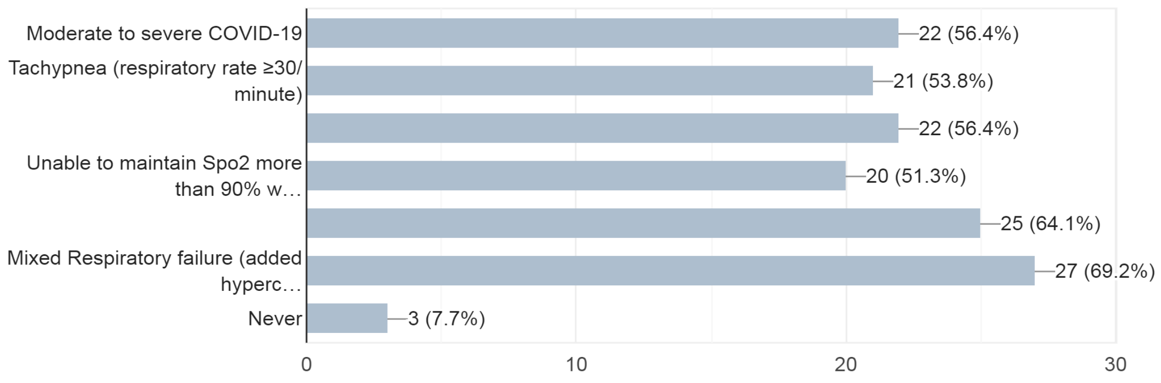

10. Based on your experience, NIV may avoid the need for tracheal intubation and INVASIVE MECHANICAL VENTILATION in patients with C-ARF.

39 responses

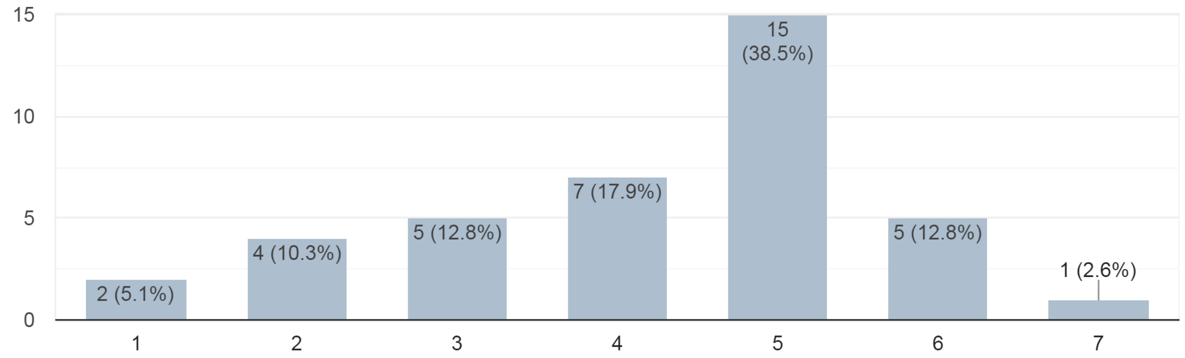

11. The use of systemic steroids could potentially avoid the need for tracheal intubation and INVASIVE MECHANICAL VENTILATION in C-ARF.

39 responses

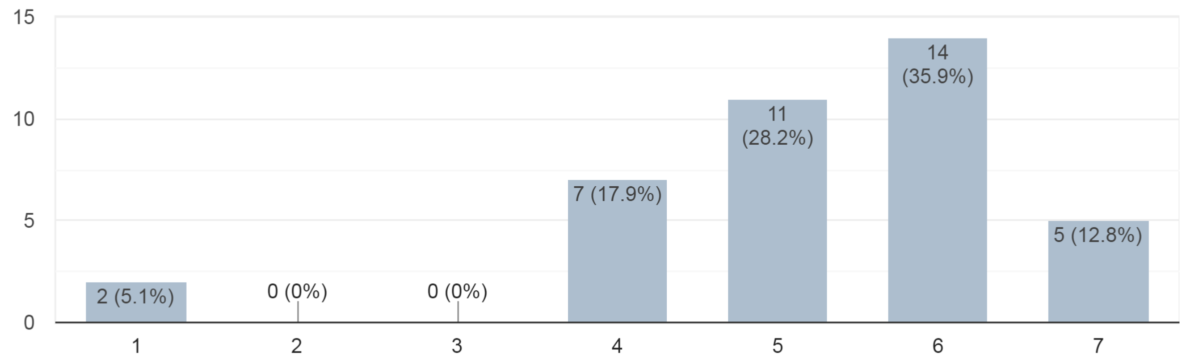

12. In which clinical context would you choose to initiate corticosteroids in C-ARF?

39 responses

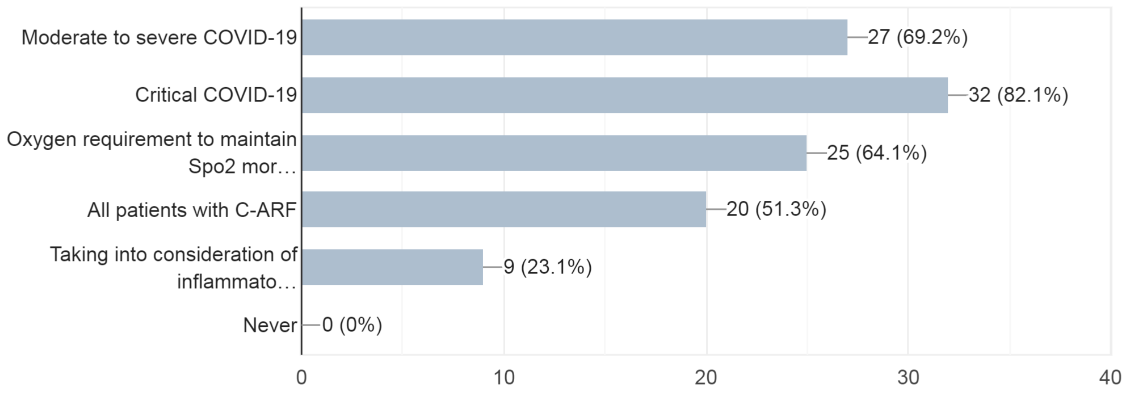

13. Which corticosteroid is your preferred choice in patients with C-ARF?

39 responses

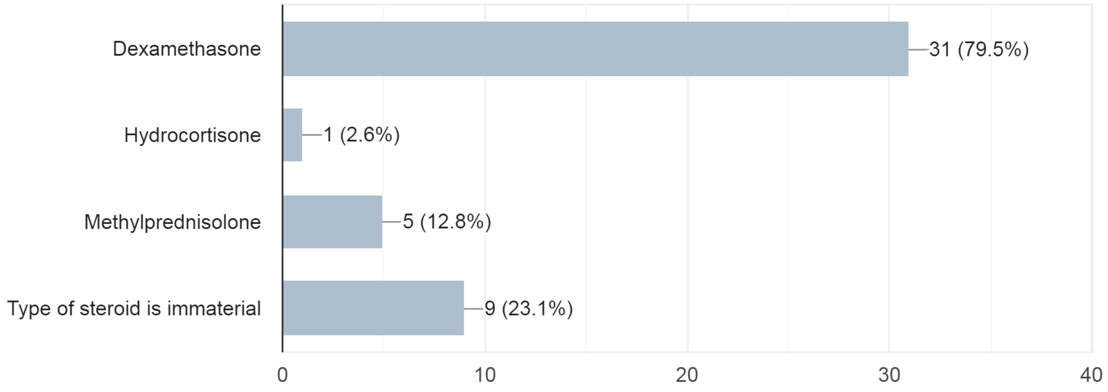

14. What daily dose of corticosteroid (equivalent dose of dexamethasone) you prescribe for C-ARF?

39 responses

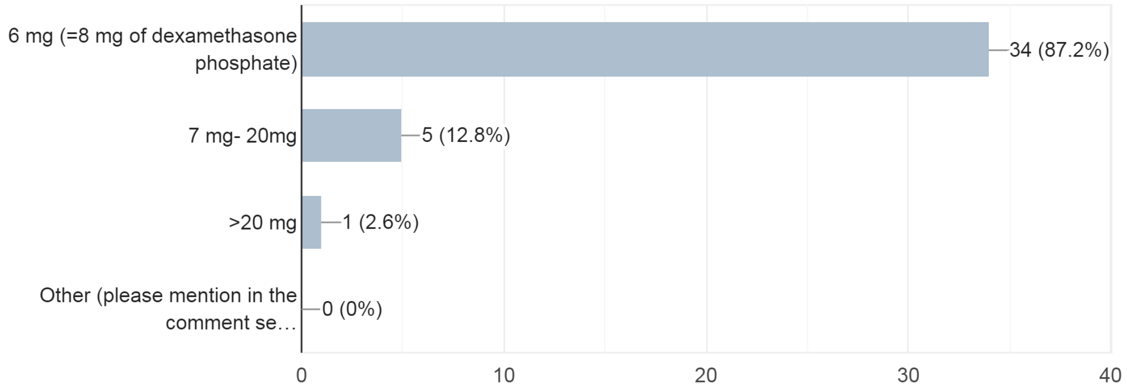

15. What duration of corticosteroid use would you prefer for patients with C-ARF?

39 responses

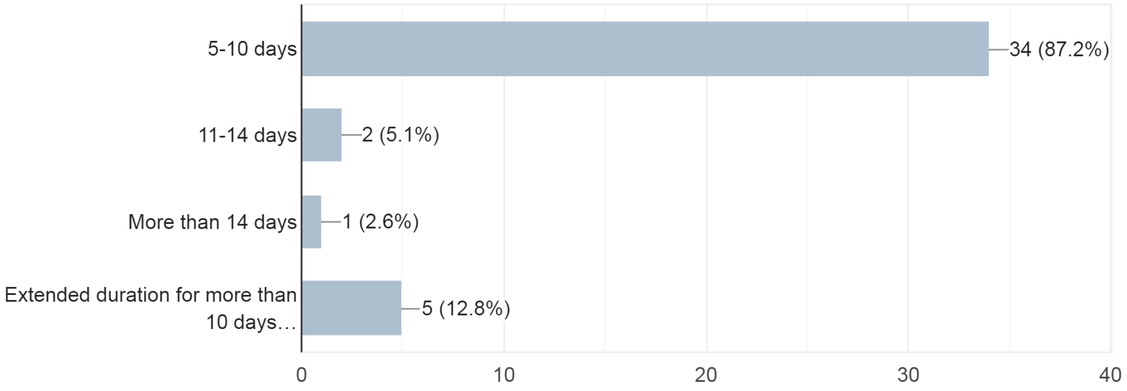

Section-2: Invasive Mechanical Ventilation

1. Which of the following options may be considered as an appropriate trigger for tracheal intubation in C-ARF?

39 responses

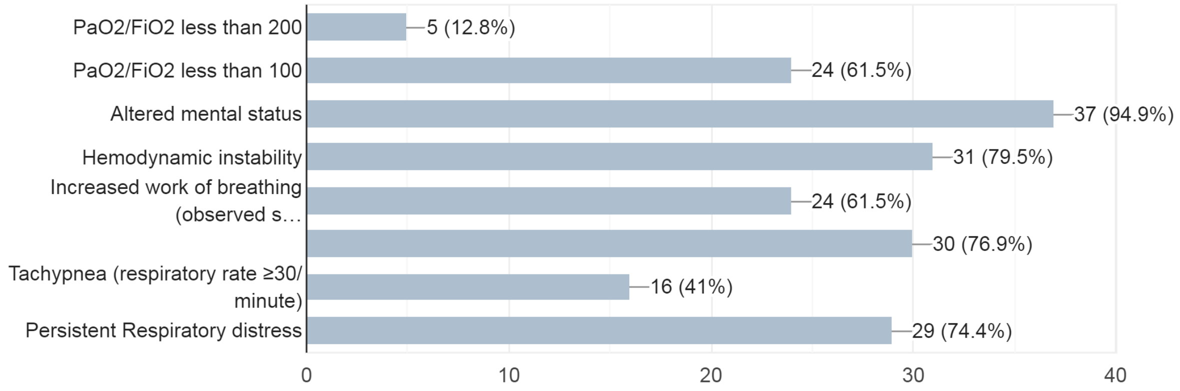

2. The lung protective ventilation (target Plateau pressure less than 30 cm of H20) strategy can be MODIFIED based on the lung compliance for C-ARF.

39 responses

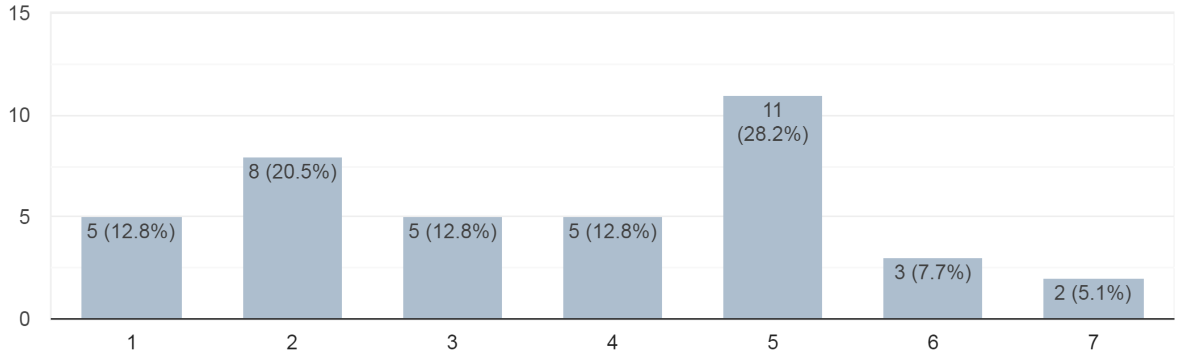

3. A low PEEP strategy (≤ 10 cm of H2O) is usually considered during invasive mechanical ventilation of C-ARF.

39 responses

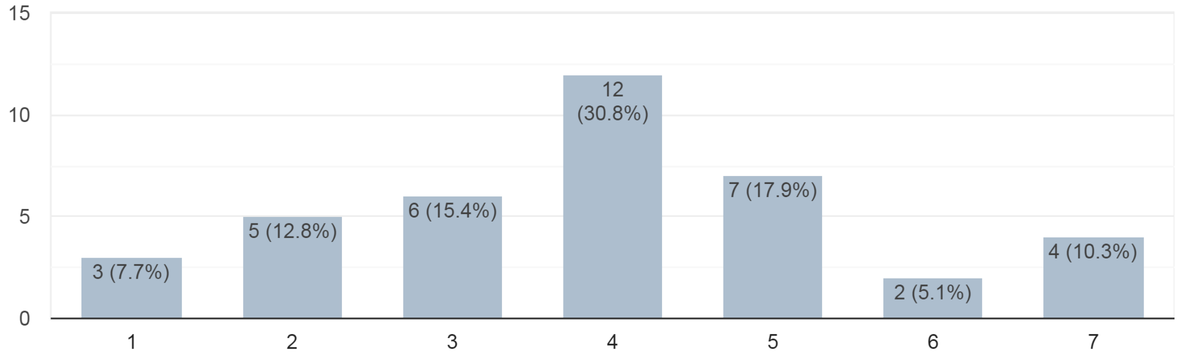

4. How would you select PEEP during invasive mechanical ventilation of C-ARF?

39 responses

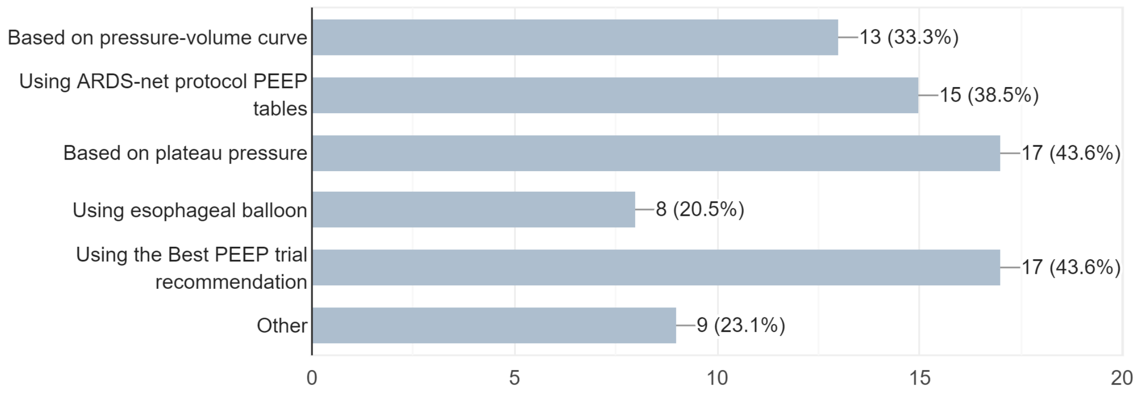

5. Neuromuscular blockade may be considered during early phase of the invasive mechanical ventilation of C-ARF to avoid patient-ventilator dyssynchrony.

39 responses

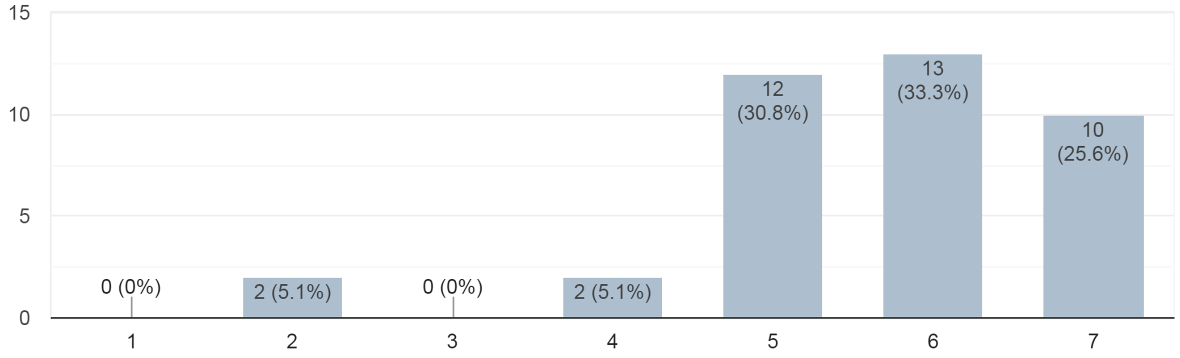

6. The invasive mechanical ventilation strategy in C-ARF should be targeted to the following?

39 responses

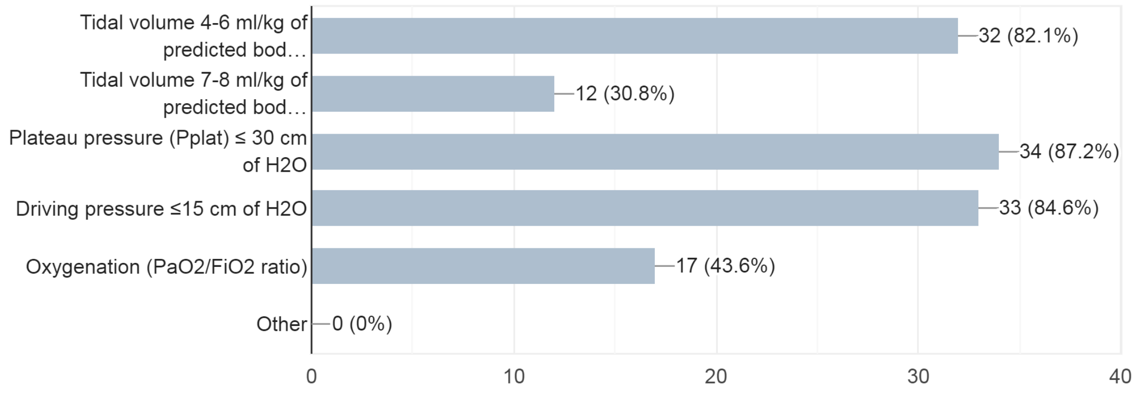

Section-3: Refractory Hypoxemia:

1. The use of recruitment maneuvers in patients with refractory hypoxemia in the setting of C-ARF needs to be personalized to the individual patient in view of its potential deleterious effects.

39 responses

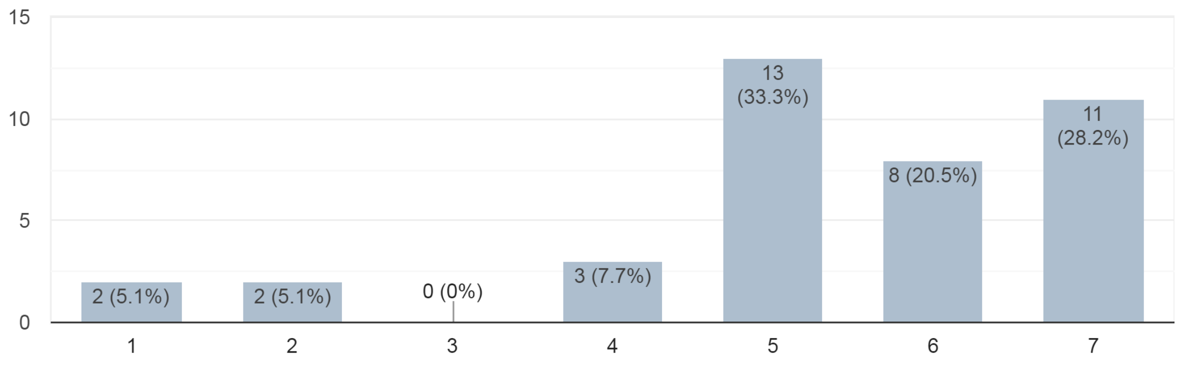

2. Prone position during invasive mechanical ventilation of C-ARF IMPROVES OXYGENATION.

39 responses

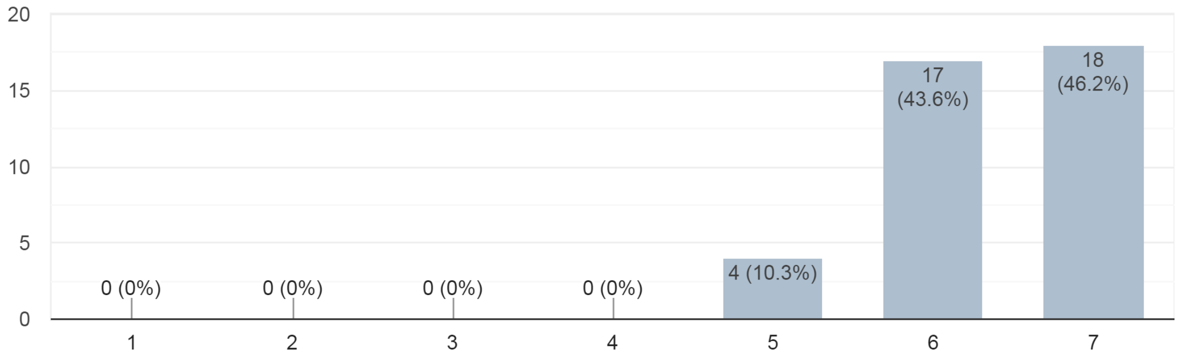

3. Prone position during invasive mechanical ventilation of C-ARF is effective when done for (duration per session)?

39 responses

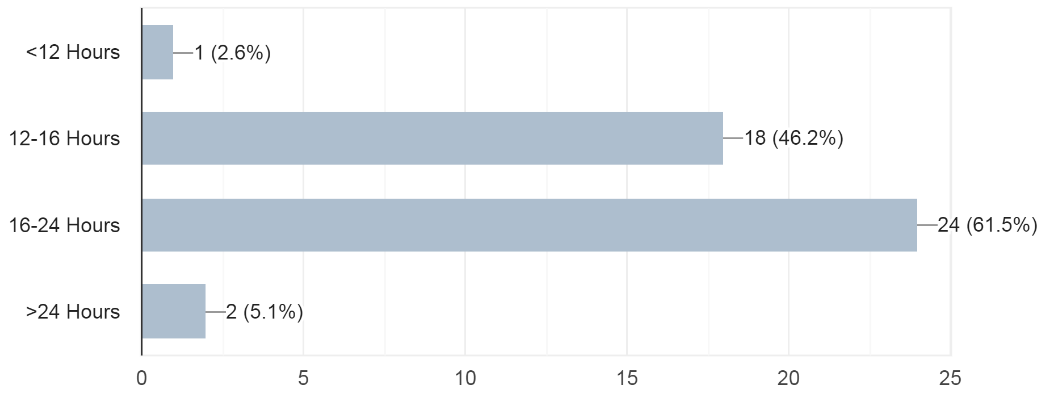

4. Advanced mechanical ventilation (APRV, PRVC, etc.) modes may be BENEFICIAL in refractory hypoxemia with C-ARF.

39 responses

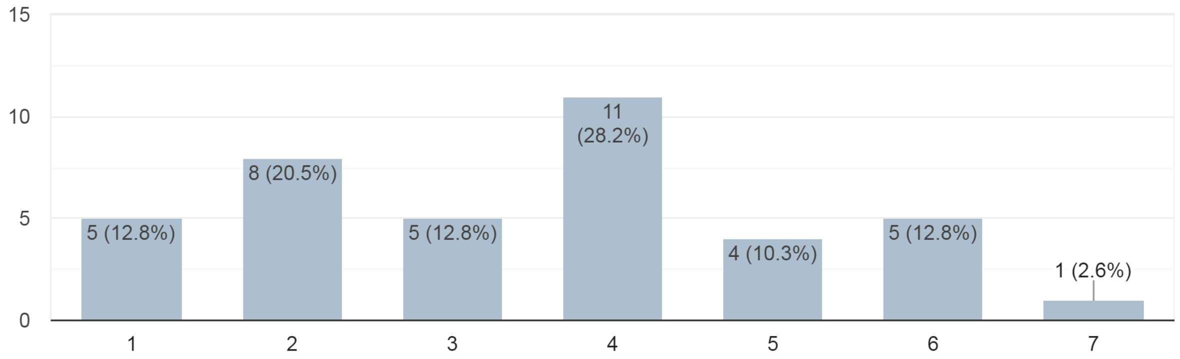

5. The following ADJUVANT therapies are effective in refractory hypoxemia with C-ARF?

39 responses

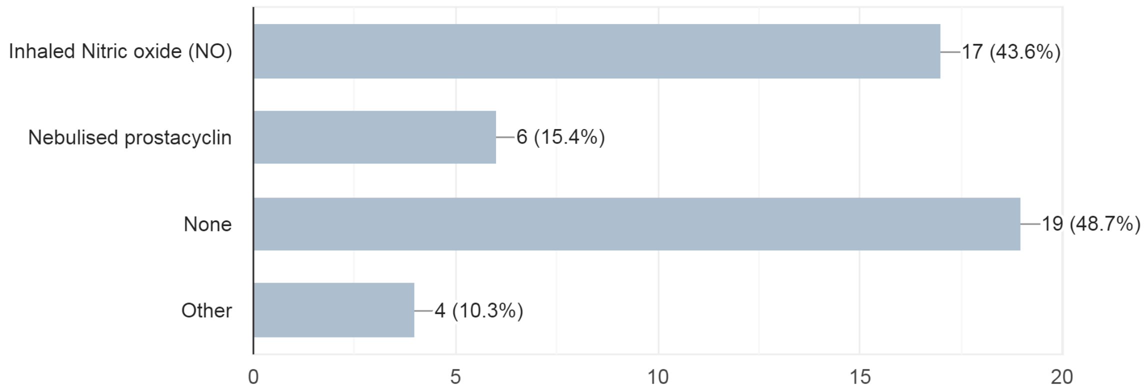

6. Veno-Venous Extracorporeal membrane oxygenation (V-V ECMO) may be considered in C-ARF patients on invasive mechanical ventilation?

39 responses

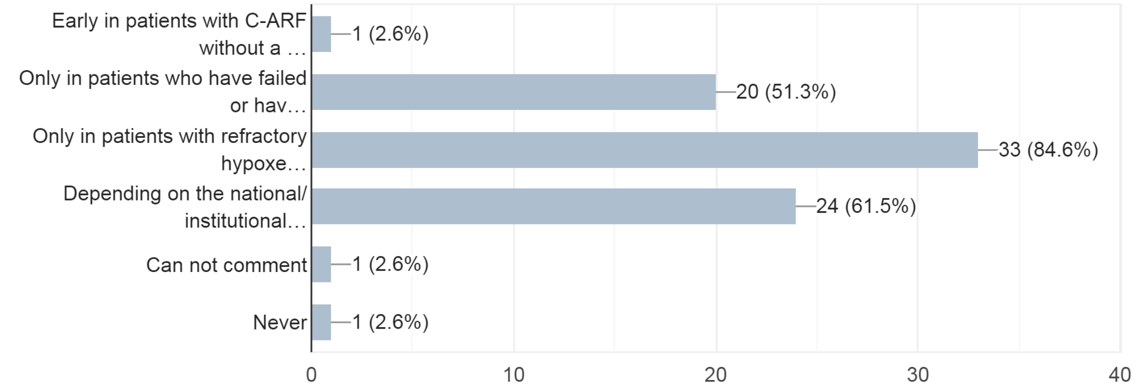

Section-4: Infection Control

1. The following are considered as aerosol-generating procedures (AGPs)?

39 responses

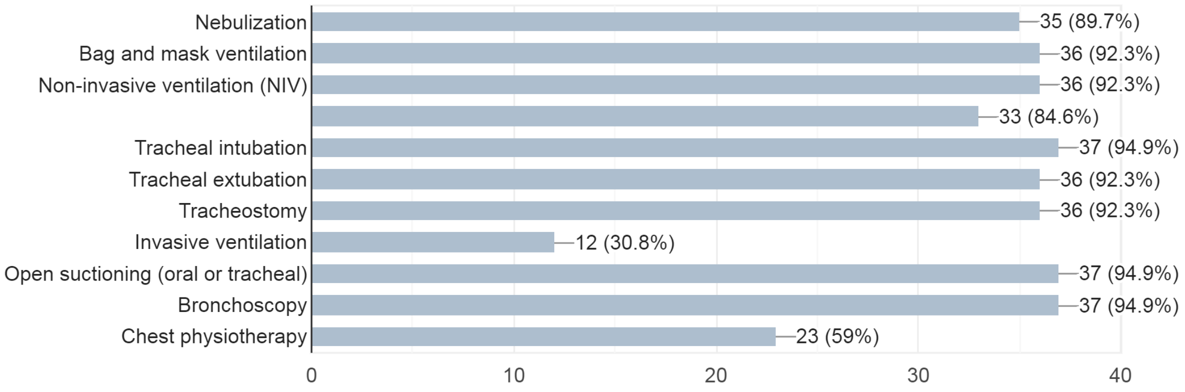

2. High flow nasal oxygen (HFNO) produces less aerosols as compared to non-invasive ventilation (NIV) with face mask.

39 responses

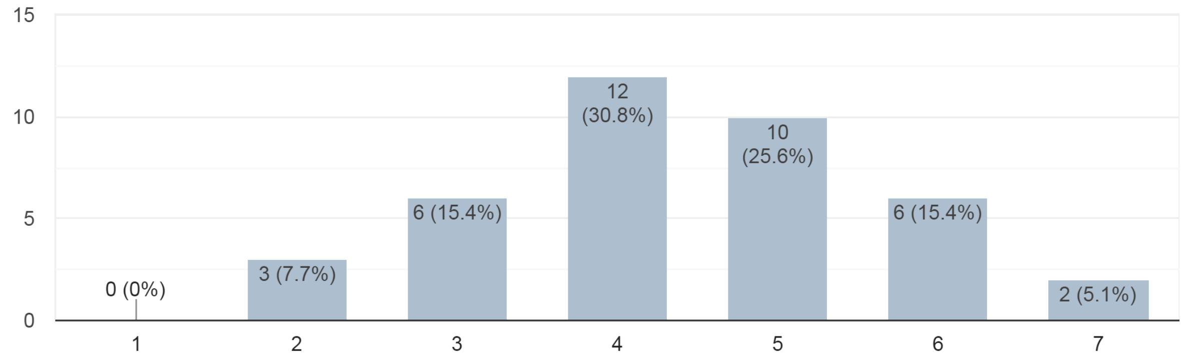

3. The following measures may be considered in the ICU to prevent cross-transmission of SARS-CoV-2?

39 responses

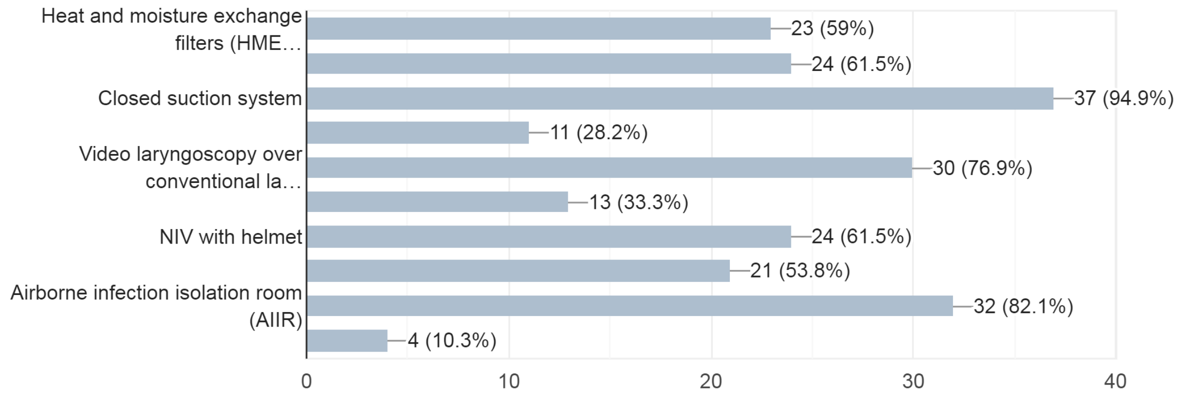

4. Which personal protective equipments (PPE) is acceptable for use during an AGP in ICU?

39 responses

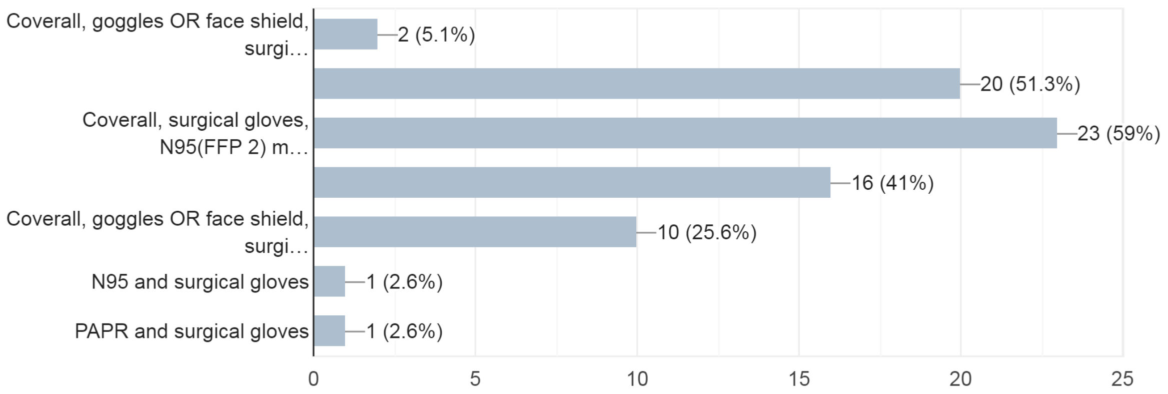

Section-5: Weaning and Tracheostomy

1. Which weaning strategy would you prefer for liberation from invasive mechanical ventilation in patients with C-ARF?

39 responses

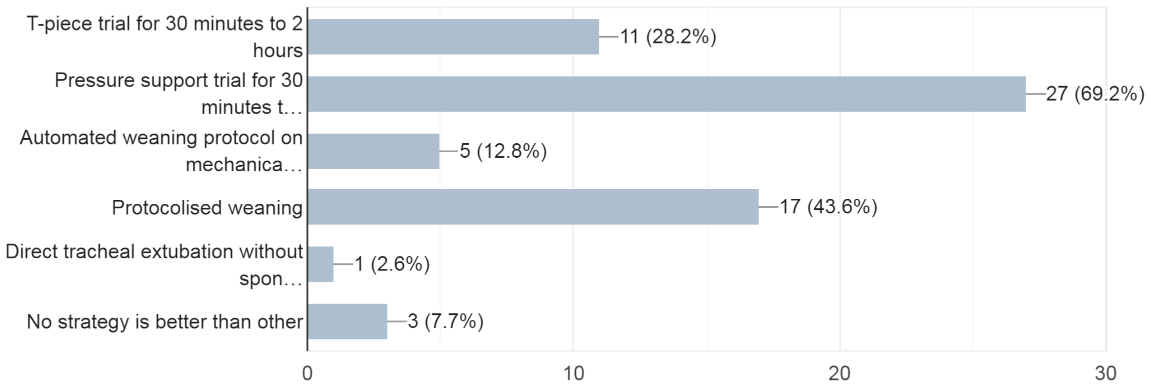

2. Chest physiotherapy could potentially benefit in the improvement of respiratory mechanics in patients with C-ARF.

39 responses

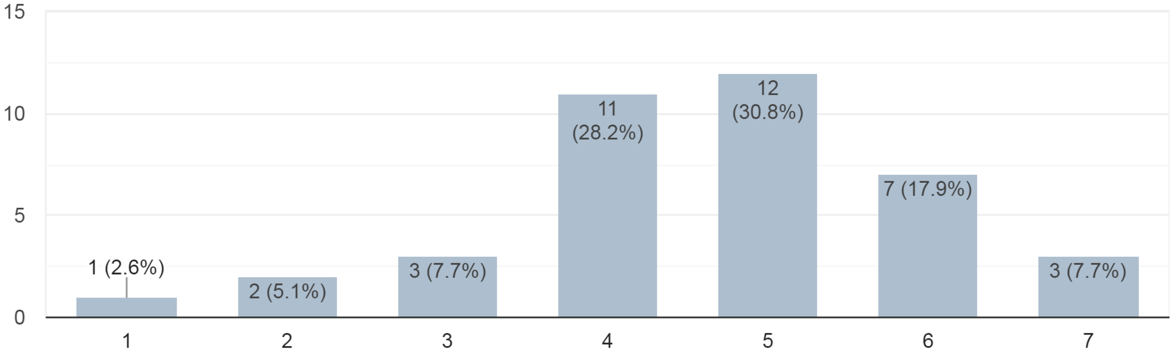

3. Early mobilization of patients is BENEFICIAL in patients on respiratory support for C-ARF.

39 responses

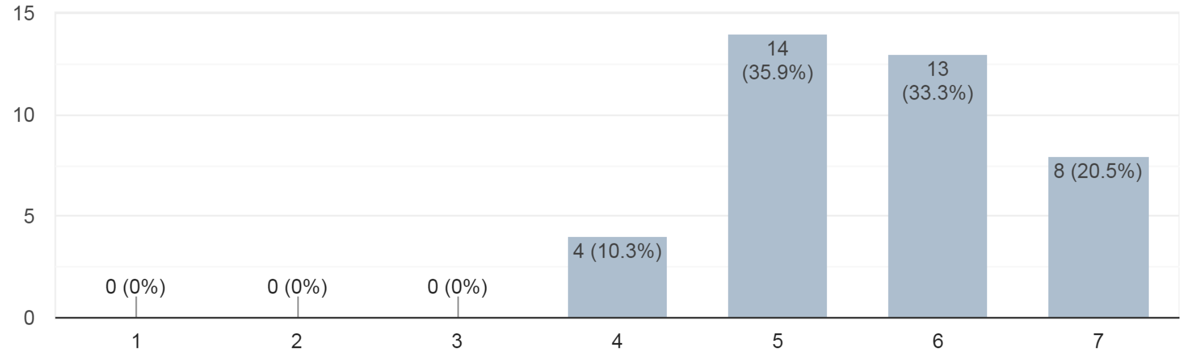

4. Delay in liberation (> 7 days) from invasive mechanical ventilation has lower risk of reintubation in patients with C-ARF.

39 responses

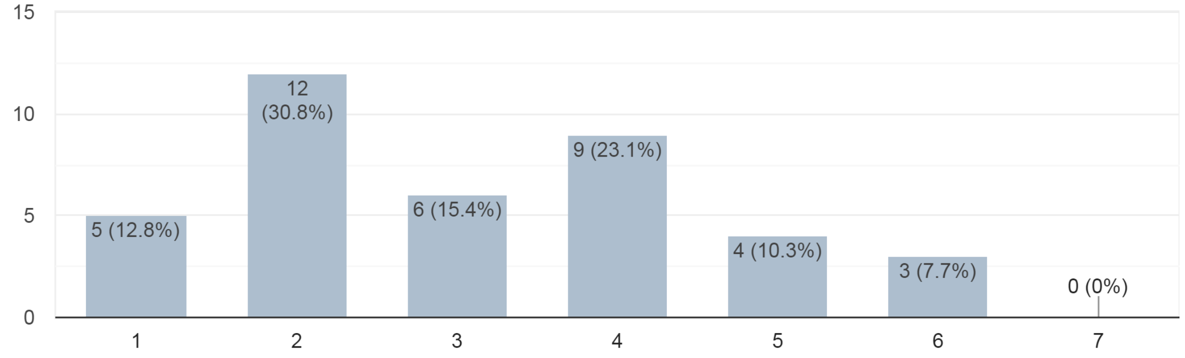

5. When should tracheostomy be considered to facilitate weaning from invasive mechanical ventilation?

39 responses

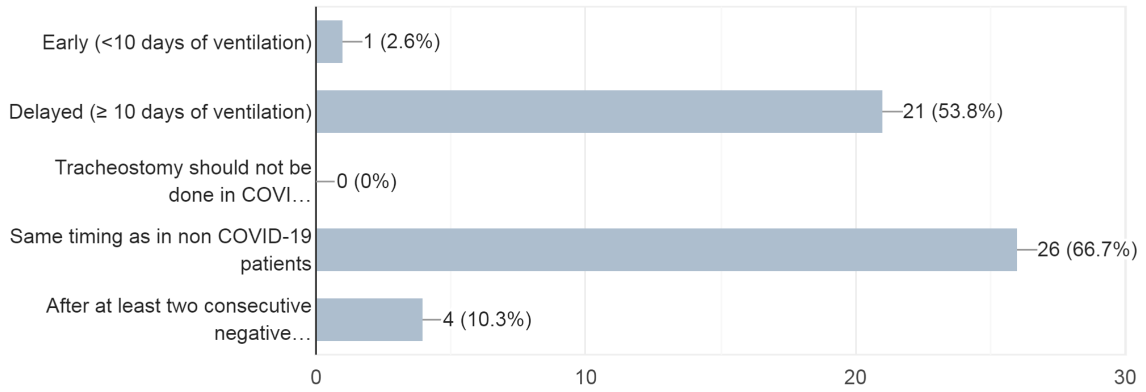

6. Which of the following technique of performing tracheostomy is preferred in patients with C-ARF ?

39 responses

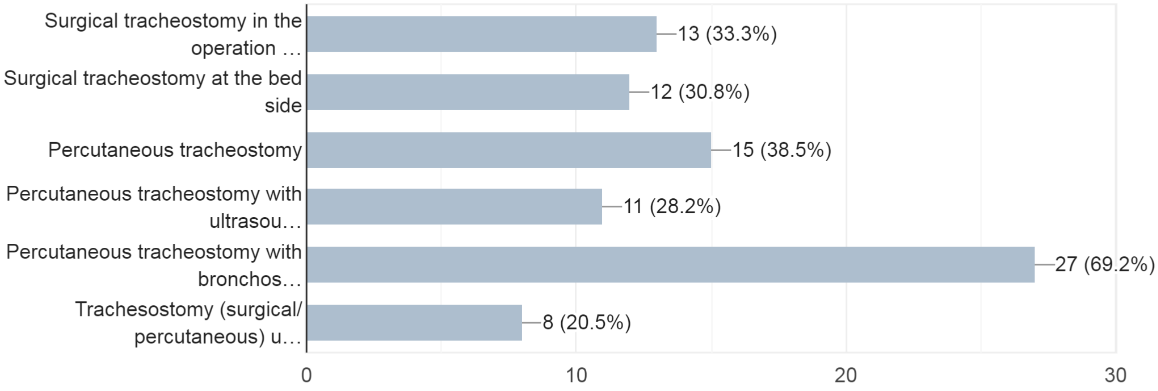

Supplement: Supplementary file 4 — Additional file 4. Survey Report 2. [file 13054_2021_3491_MOESM4_ESM.pdf]
