# Supplementary material for: Expert consensus statements for the management of COVID-19-related acute respiratory failure using a Delphi method
Source: Crit Care. 2021 Mar 16;25:106. doi: 10.1186/s13054-021-03491-y (PMC7962430; doi:10.1186/s13054-021-03491-y)
Supplement: Supplementary file 5 — Additional file 5. Survey Report 3. [file 13054_2021_3491_MOESM5_ESM.pdf]

Report of Survey Questionnaire Three

Section-1: Non-invasive Respiratory Interventions

1. The pathophysiology of COVID-19 related to acute respiratory failure (C-ARF) is similar to that of acute respiratory distress syndrome (ARDS).

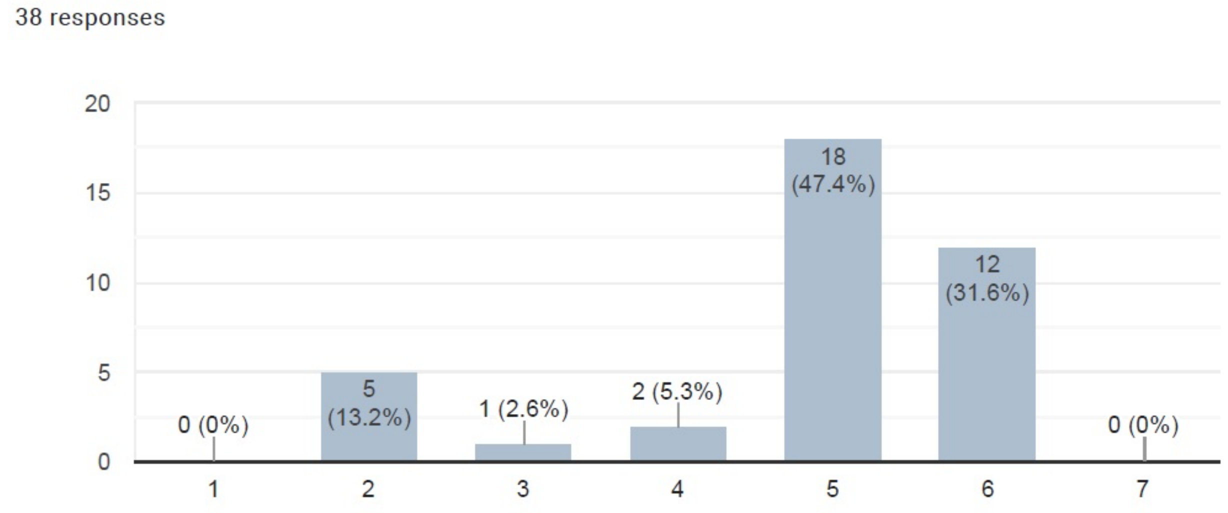

Consensus: Towards agreement
Stability: Not achieved

2. Based on your experience, awake self Proning MAY IMPROVE OXYGENATION in patients with C-ARF.

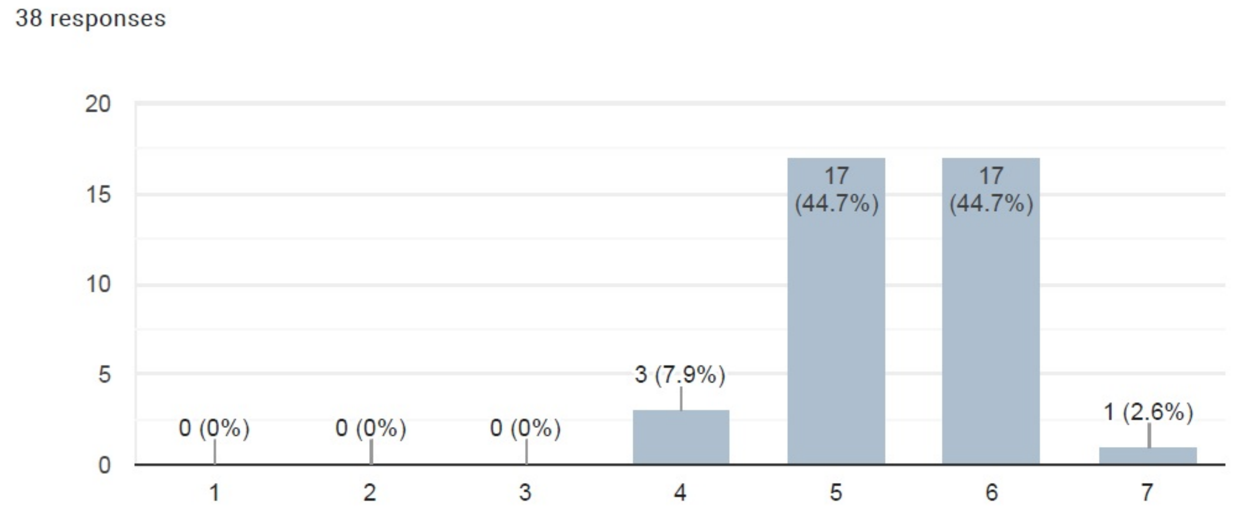

Consensus: Towards agreement
Stability: Stable

3. Based on your experience, awake self proning MAY prevent the need for invasive MECHANICAL VENTILATION in patients with C-ARF.

38 responses

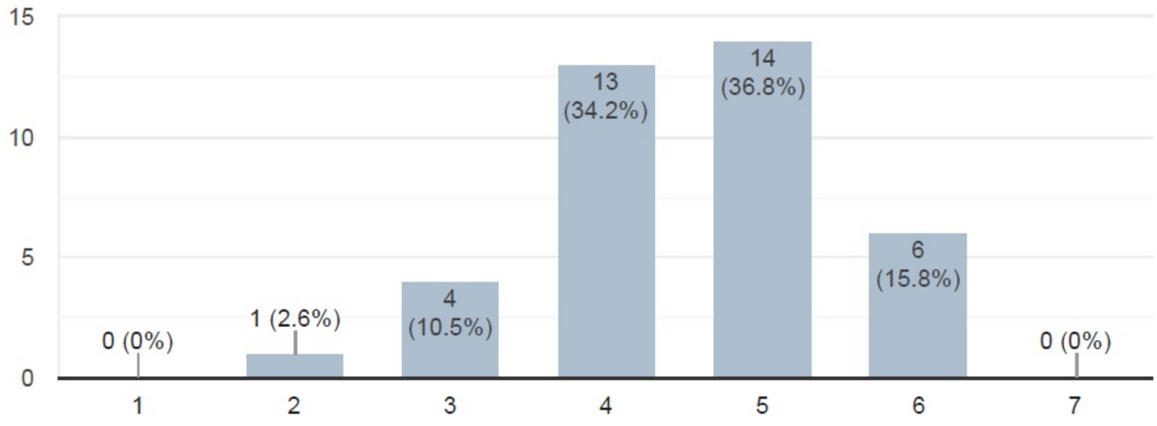

Consensus: Towards Agreement
Stability: Stable

4. In which of the following clinical scenarios should awake self proning be initiated in patients with C-ARF?

38 responses

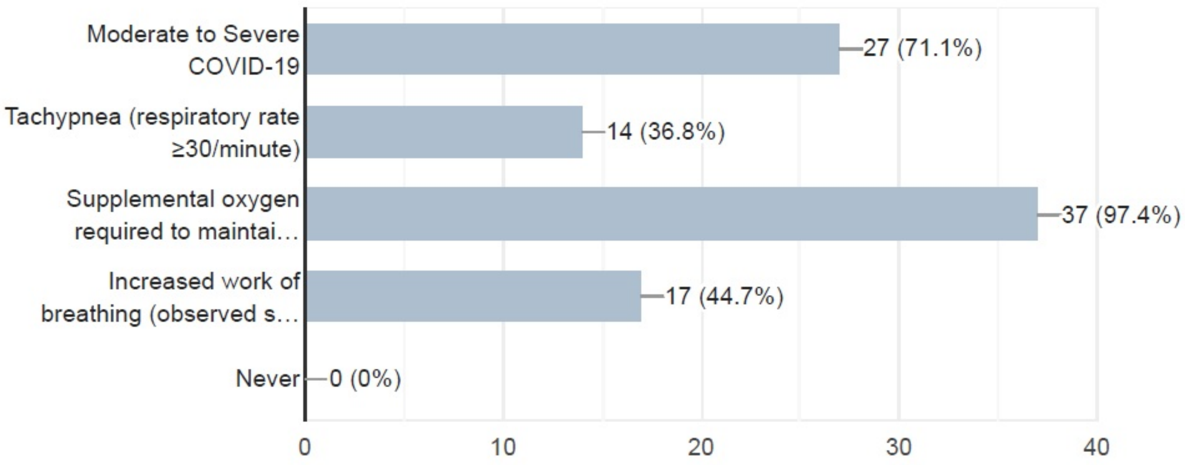

Consensus: Towards agreement on option 3
Stability: Stable

5. High flow nasal oxygen (HFNO) can be considered as an ALTERNATIVE STRATEGY for oxygen support before invasive mechanical ventilation.

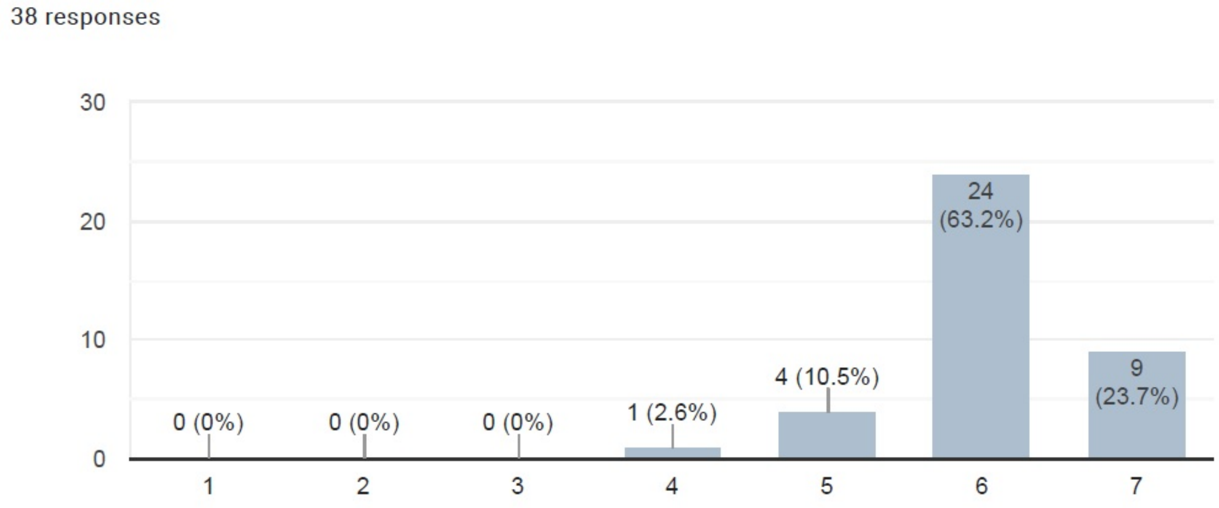

Consensus: Towards Agreement
Stability: Stable

6. When do you initiate HFNO in patients with C-ARF?

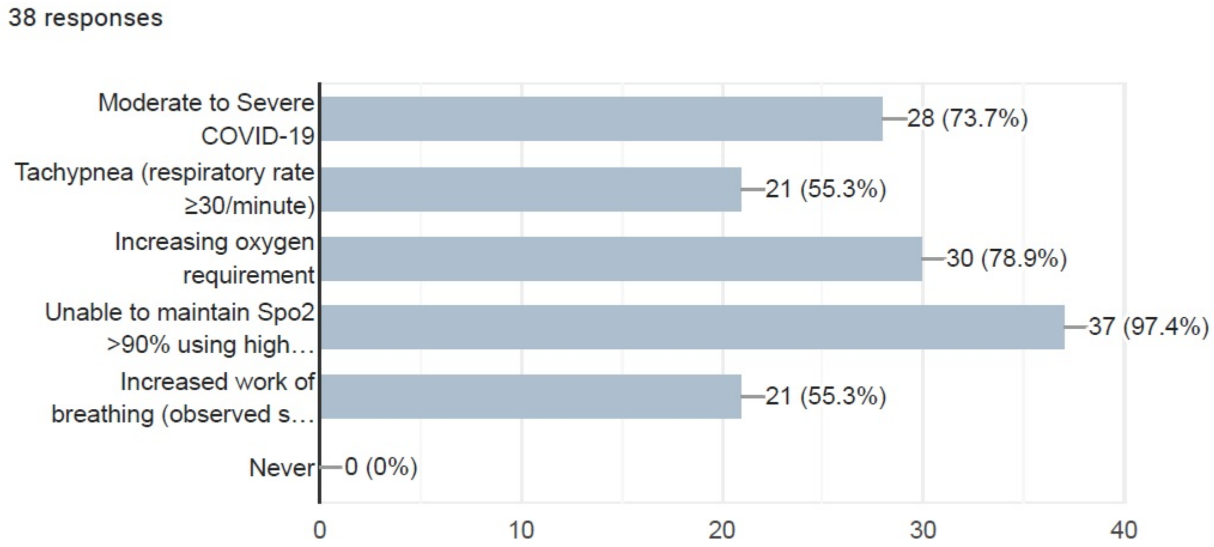

Consensus: Option 4 towards agreement
Stability: Stable

7. Based on your experience, HFNO may avoid the need for tracheal intubation and INVASIVE MECHANICAL VENTILATION in patients with C-ARF.

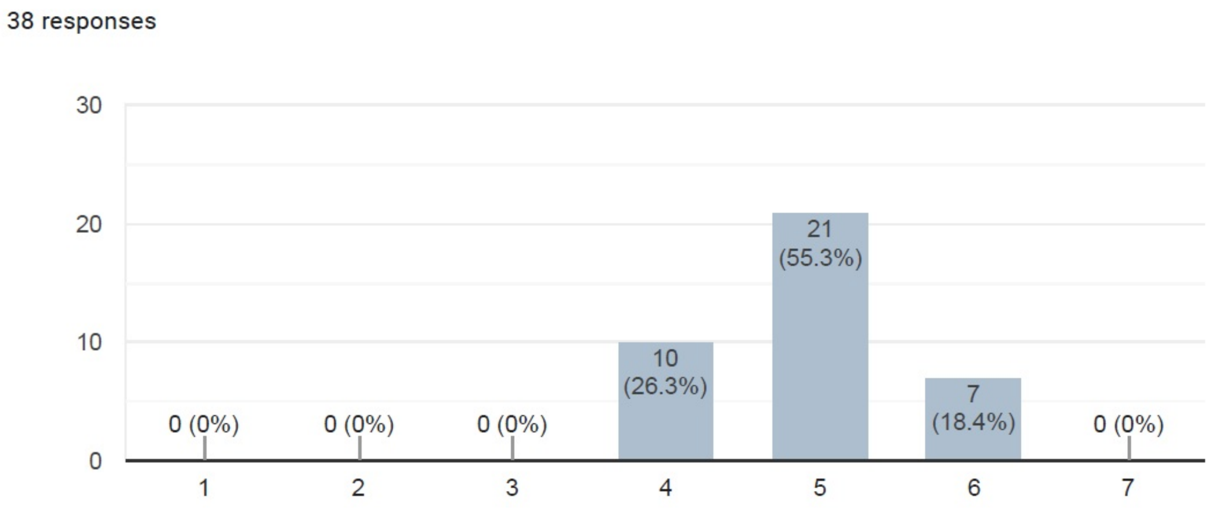

Consensus: Towards Agreement
Stability: Not stable.

8. Non-invasive ventilation (NIV) can be considered as an ALTERNATIVE STRATEGY for oxygen support before invasive mechanical ventilation.

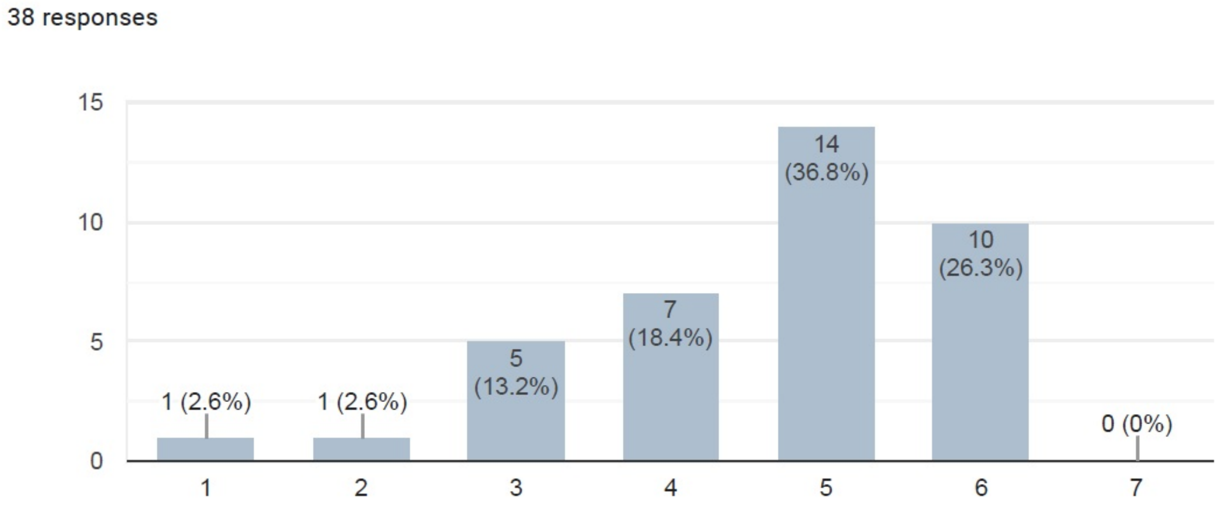

Consensus: Towards Agreement
Stability: Stable

9. NIV may be considered in the following clinical scenarios in patients with C-ARF?

38 responses

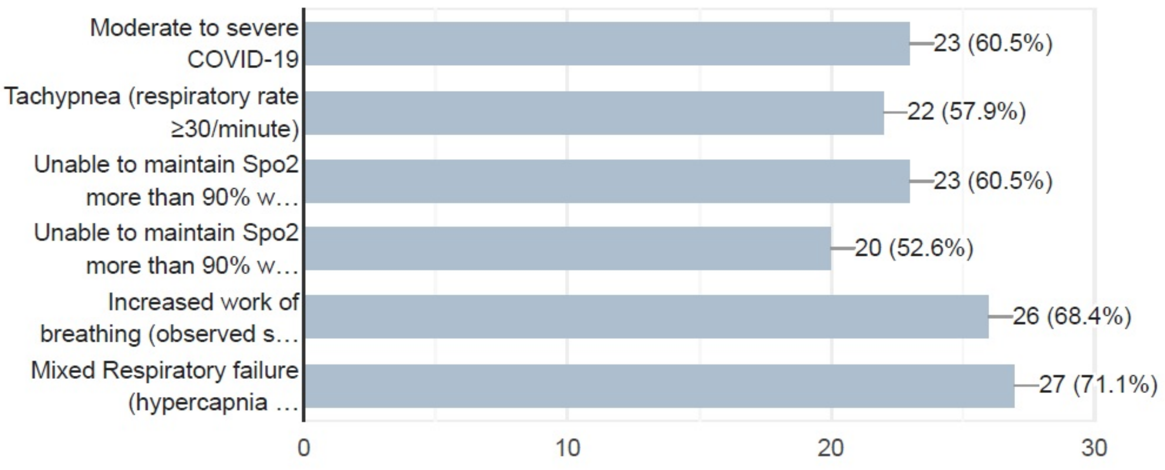

Consensus: None
Stability: Comparison not available, as language of this question was changed in round three.

10. Based on your experience, NIV may avoid the need for tracheal intubation and INVASIVE MECHANICAL VENTILATION in patients with C-ARF.

38 responses

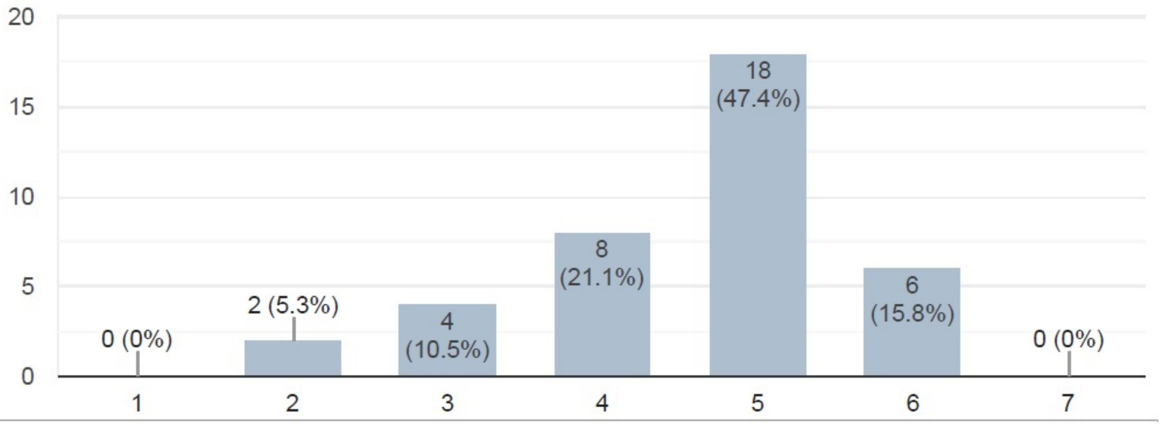

Consensus: None
Stability: Stable

11. The use of systemic steroids could potentially avoid the need for tracheal intubation and INVASIVE MECHANICAL VENTILATION in C-ARF.

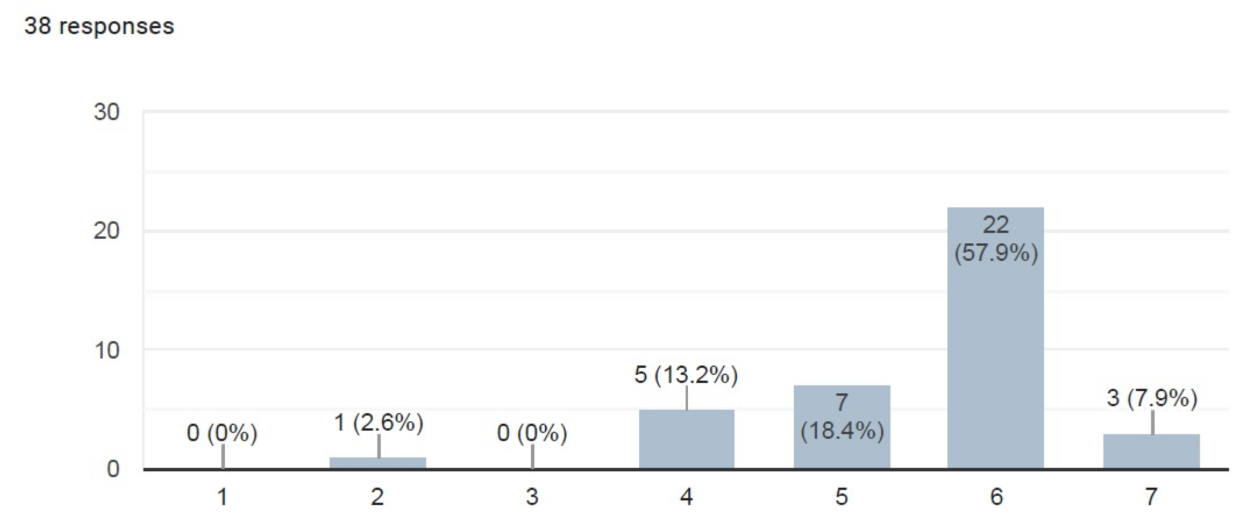

Consensus: Towards Agreement
Stability: Stable

12. In which clinical context would you choose to initiate corticosteroids in C-ARF?

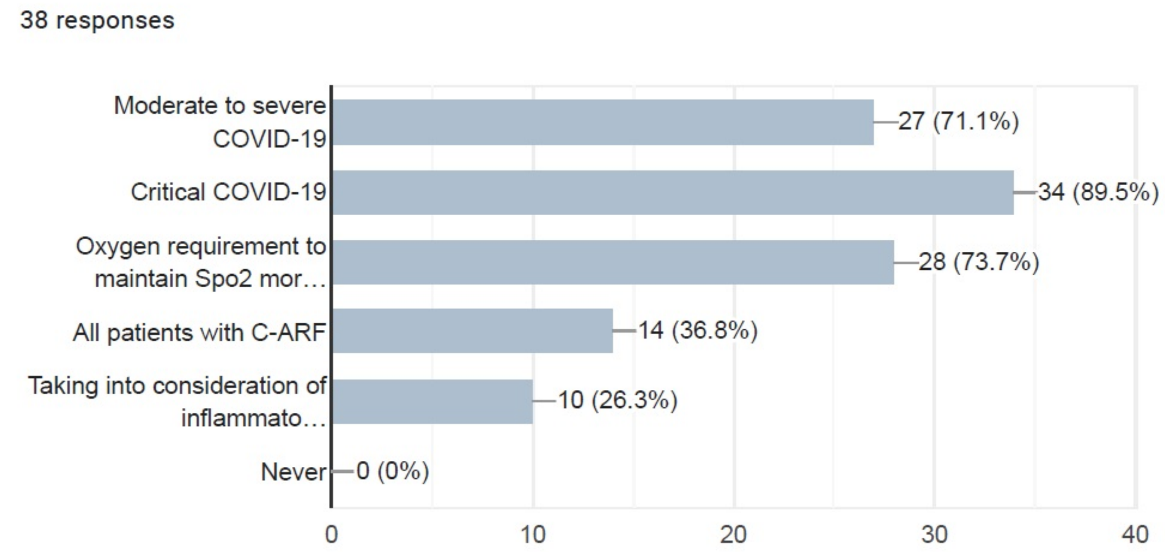

Consensus: Option 2 towards agreement
Stability: Stable

13. Which corticosteroid is your preferred choice in patients with C-ARF?

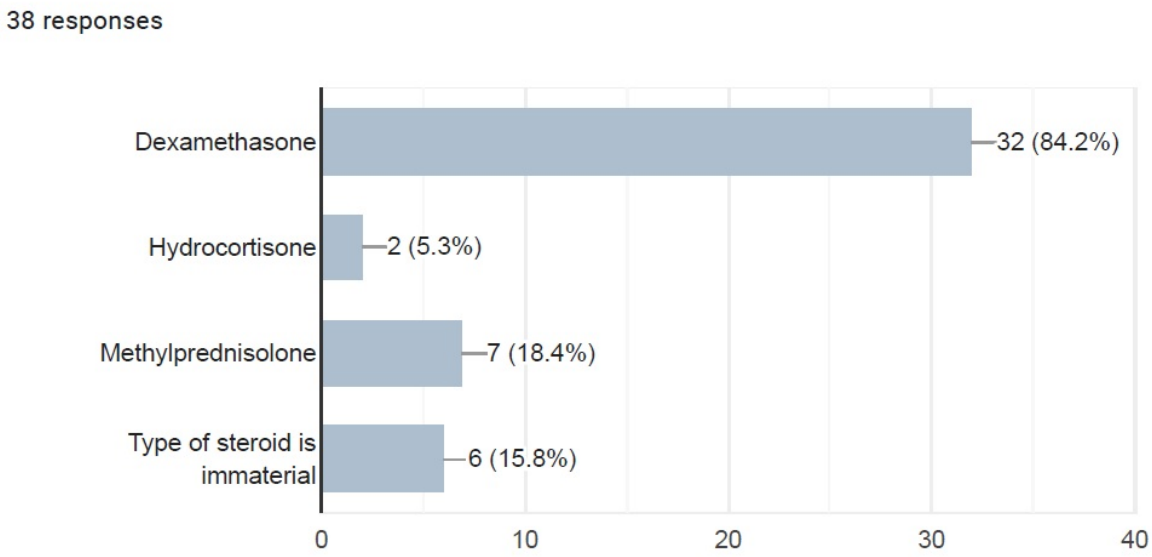

Consensus: Option 1 towards agreement
Stability: Stable

14. What daily dose of corticosteroid (equivalent dose of dexamethasone) you prescribe for C-ARF?

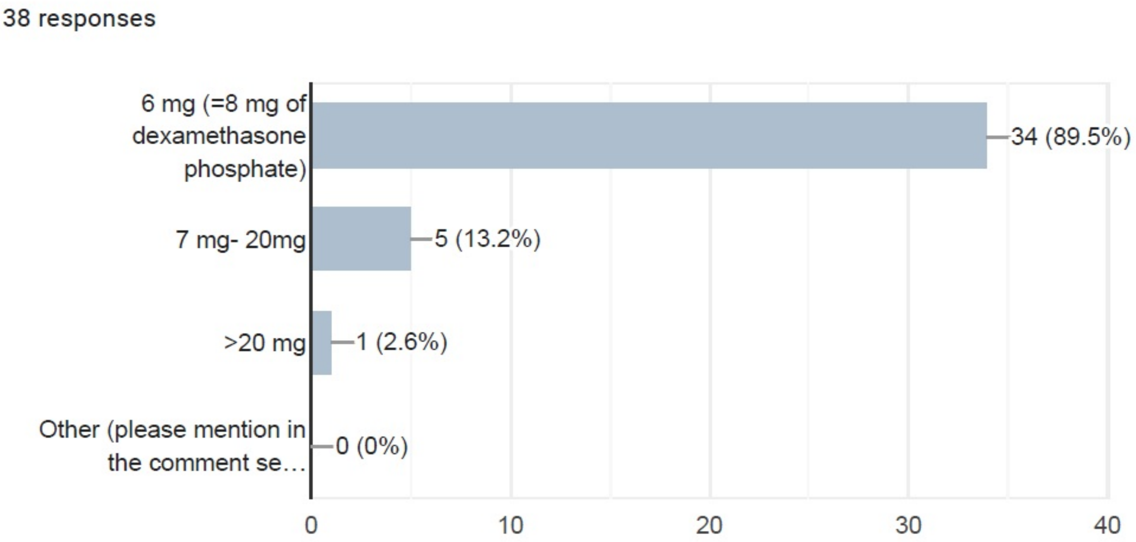

Consensus: Option 1 towards agreement
Stability: Stable

15. What duration of corticosteroid use would you prefer for patients with C-ARF?

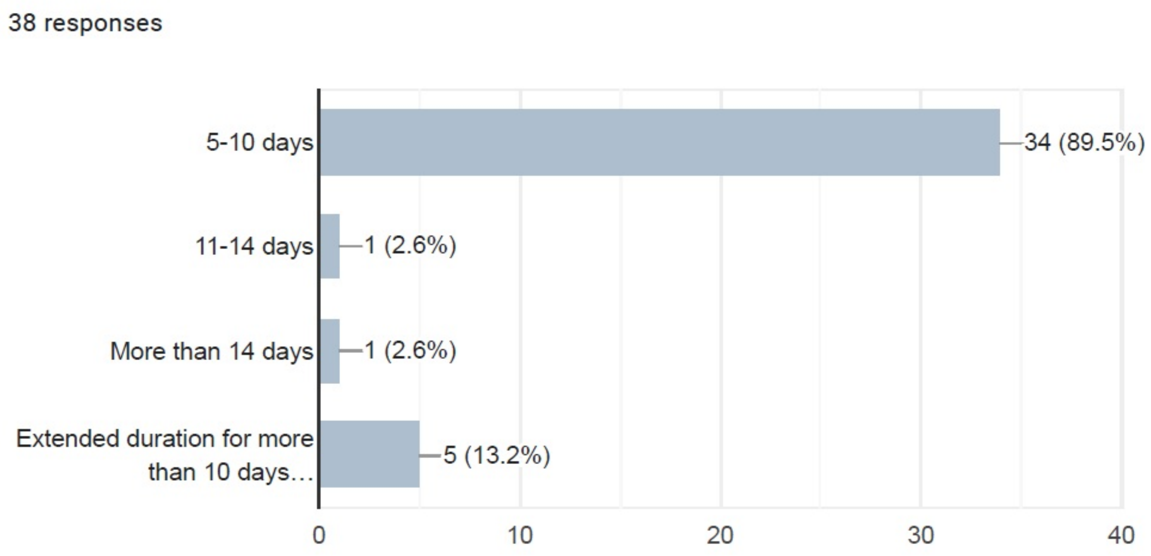

Consensus: Option 1 towards agreement.
Stability: Stable

Section-2: Invasive Mechanical Ventilation

1. Which of the following options may be considered as an appropriate trigger for tracheal intubation in C-ARF?

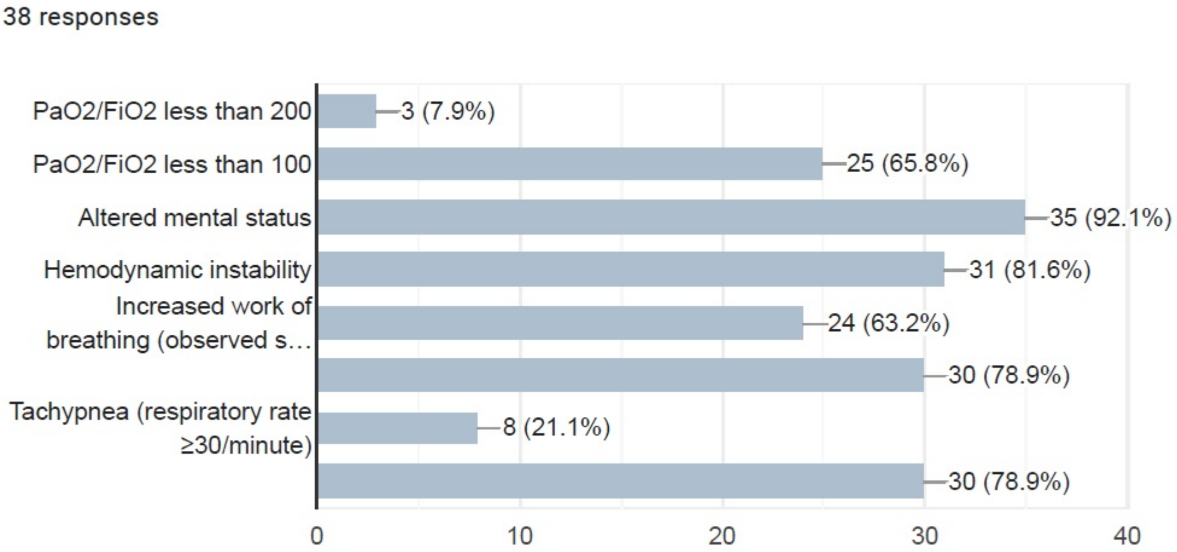

Consensus: Option 3 & 4 towards agreement
Stability: Stable

2. "Lung protective ventilation" should be used for patients with C-ARF on invasive mechanical ventilation.

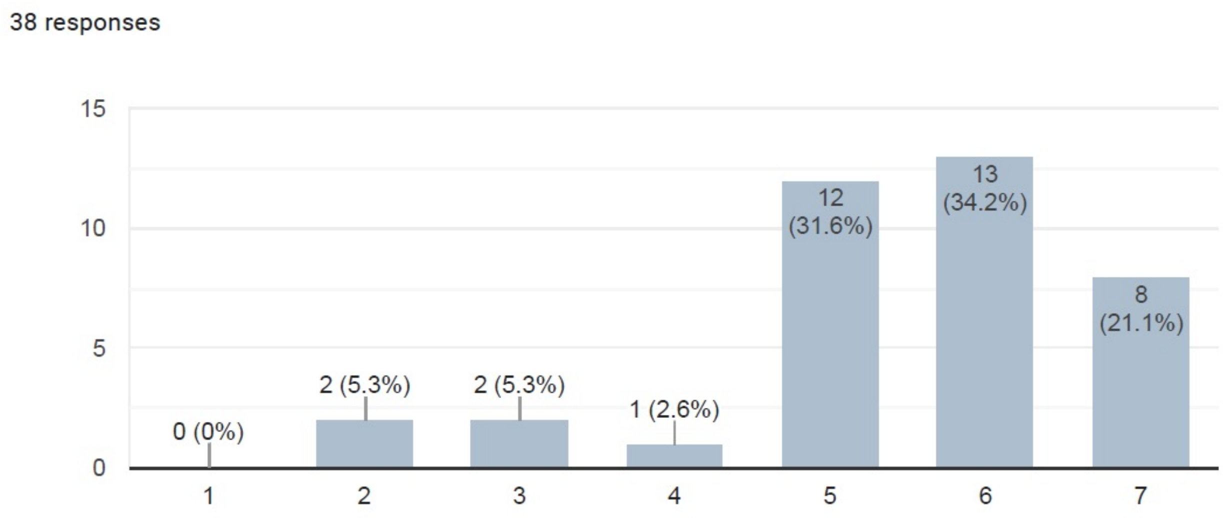

Consensus: Towards Agreement
Stability: Comparison not available, as language of this question was changed in round three.

3. A low PEEP strategy (≤ 10 cm of H2O) is usually considered during invasive mechanical ventilation of C-ARF.

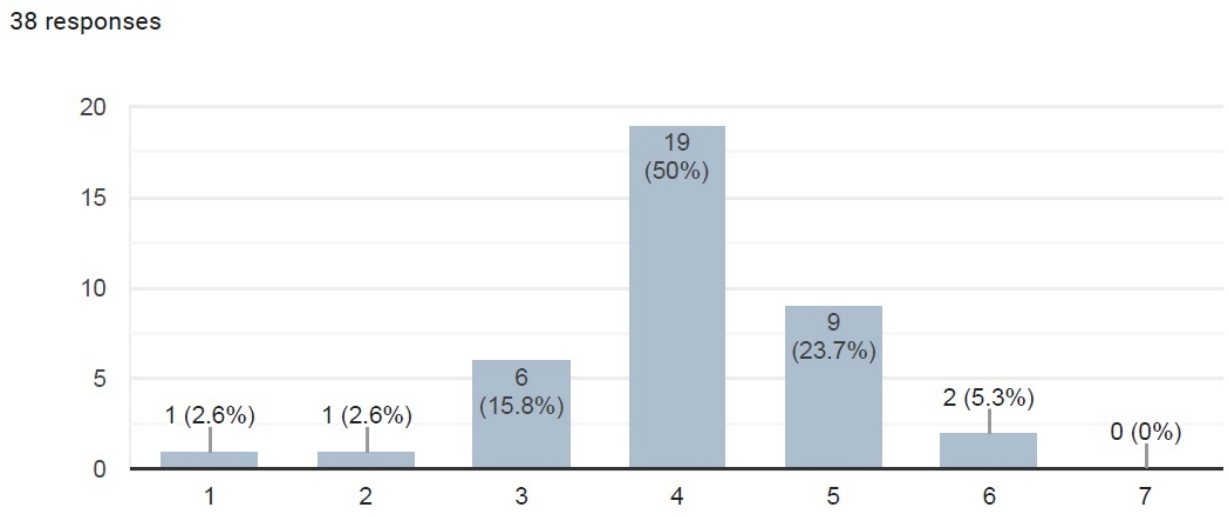

Consensus: None
Stability: Not stable

4. How would you select PEEP in a patient of C-ARF on invasive mechanical ventilation with thorax CT scan showing bilateral pulmonary infiltrates, PaO2/FiO2 ratio less than 100, plateau pressure 27 cm of H20, and PEEP of6 cm of H20?

38 responses

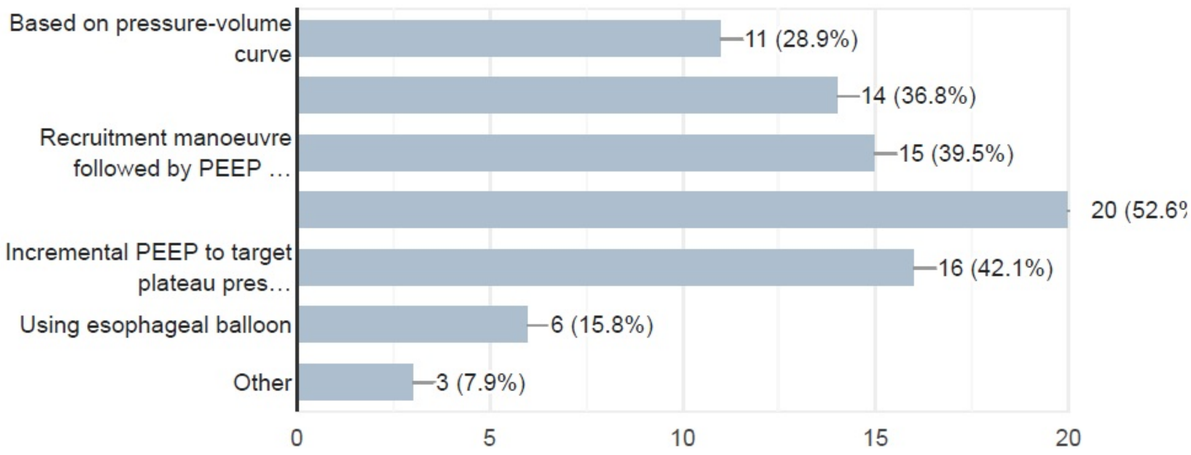

Consensus: None
Stability: Comparison not available, as language of this question was changed in round three.

5. Neuromuscular blockade MAY BE considered during the early phase of the invasive mechanical ventilation of C-ARF to avoid patient-ventilator dyssynchrony.

38 responses

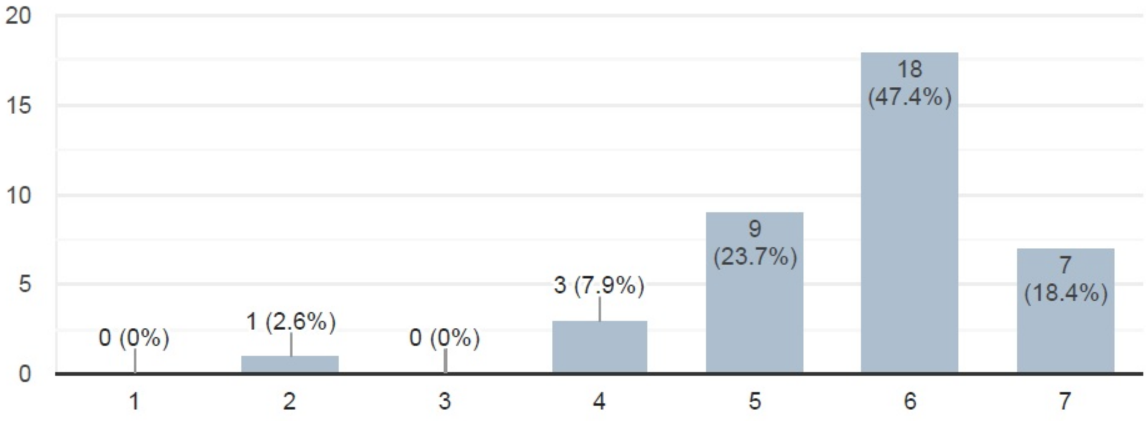

Consensus: Towards agreement
Stability: Stable

6. The invasive mechanical ventilation strategy in C-ARF should be targeted to the following?

38 responses

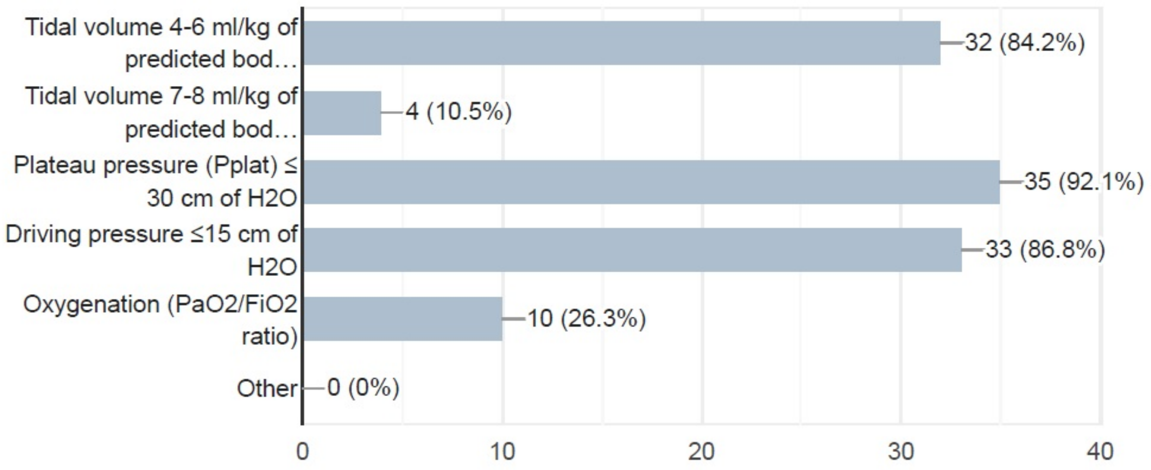

Consensus: Options 1,3, & 4 towards agreement
Stability: Not stable.

Section-3: Refractory Hypoxemia

1. The use of recruitment maneuvers in patients with refractory hypoxemia in the setting of C-ARF needs to be personalized to the individual patient in view of its potential deleterious effects.

38 responses

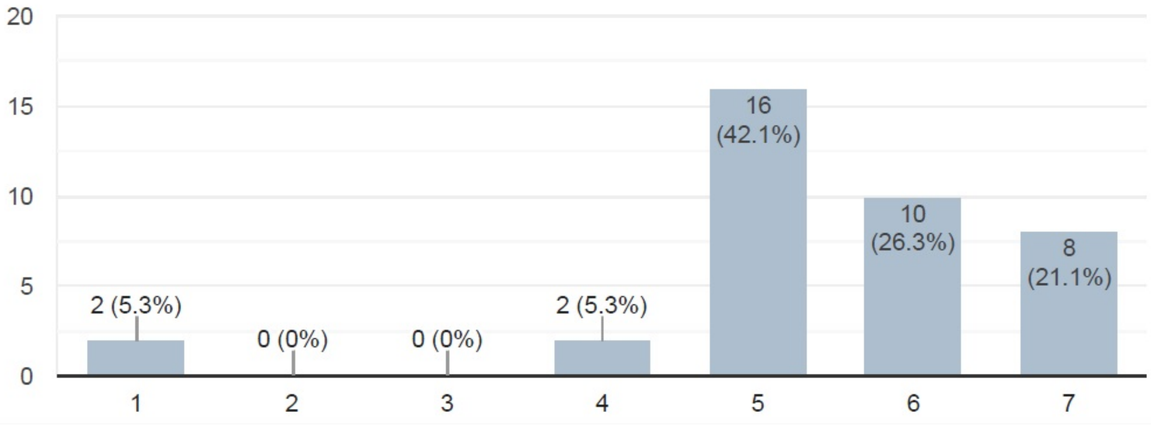

Consensus: Towards agreement
Stability: Stable.

2. Prone position during invasive mechanical ventilation of C-ARF IMPROVES OXYGENATION.

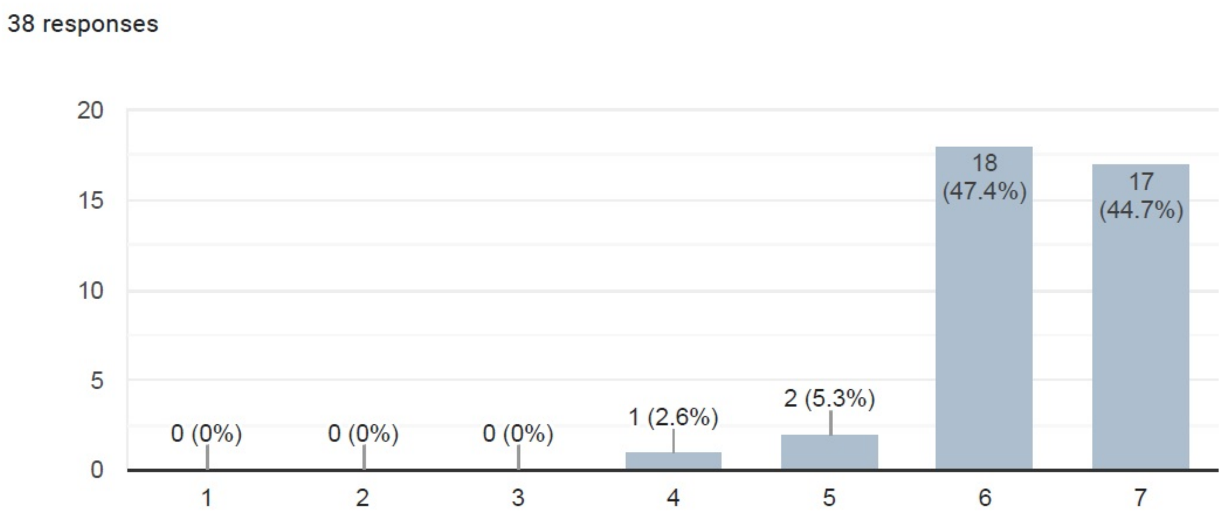

Consensus: Towards agreement
Stability: Stable

3. Prone position during invasive mechanical ventilation of C-ARF is effective when done for (duration per session)?

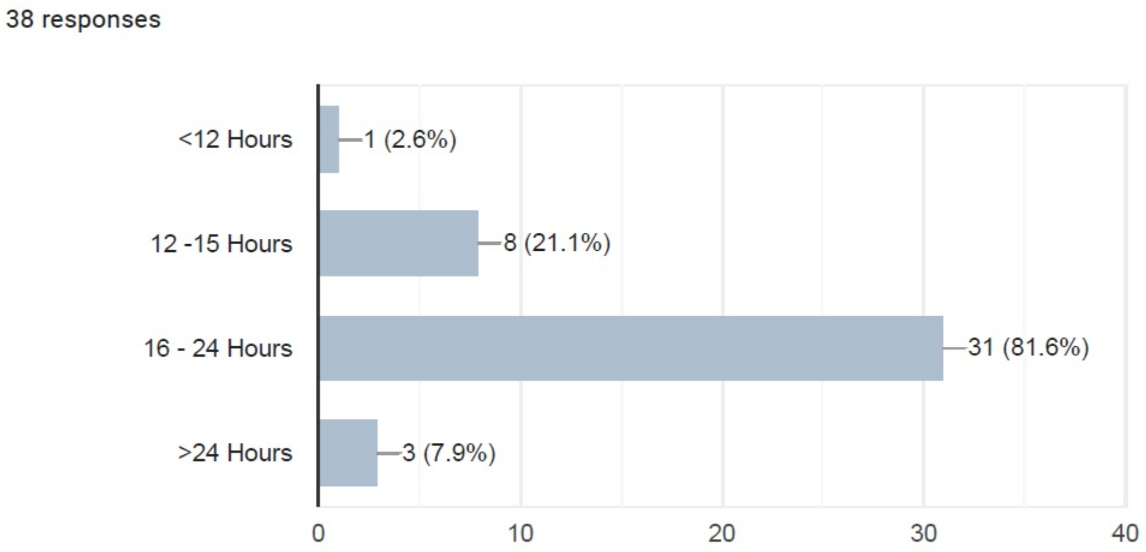

Consensus: Option 3 towards agreement.
Stability: Comparison not available, as language of this question was changed in round three.

4. Advanced mechanical ventilation (APRV, PRVC, etc.) modes may be BENEFICIAL in refractory hypoxemia with C-ARF.

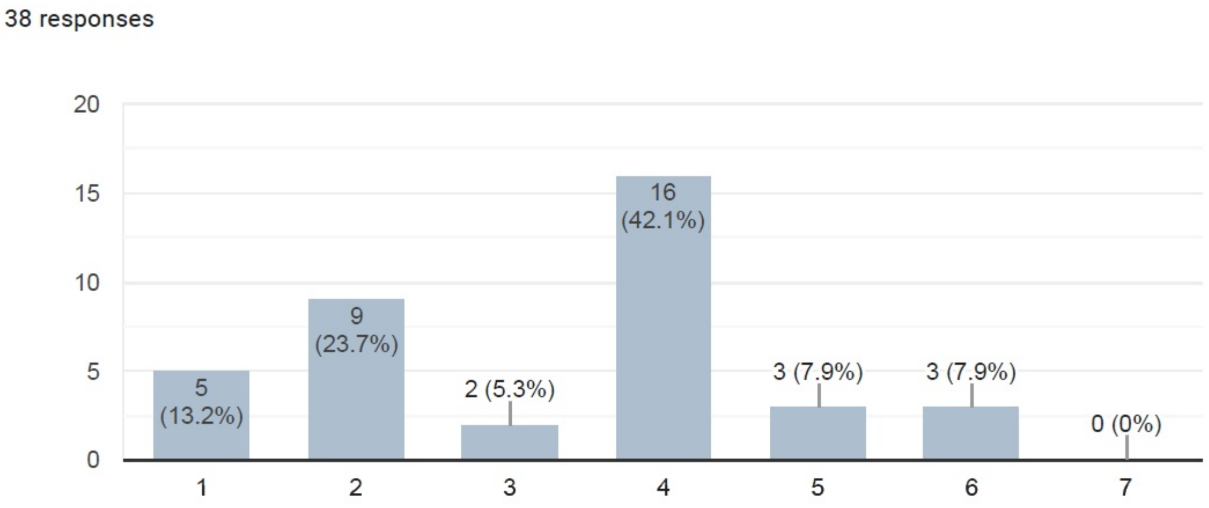

Consensus: None
Stability: Not stable.

5. The following ADJUVANT therapies are effective in refractory hypoxemia with C-ARF?

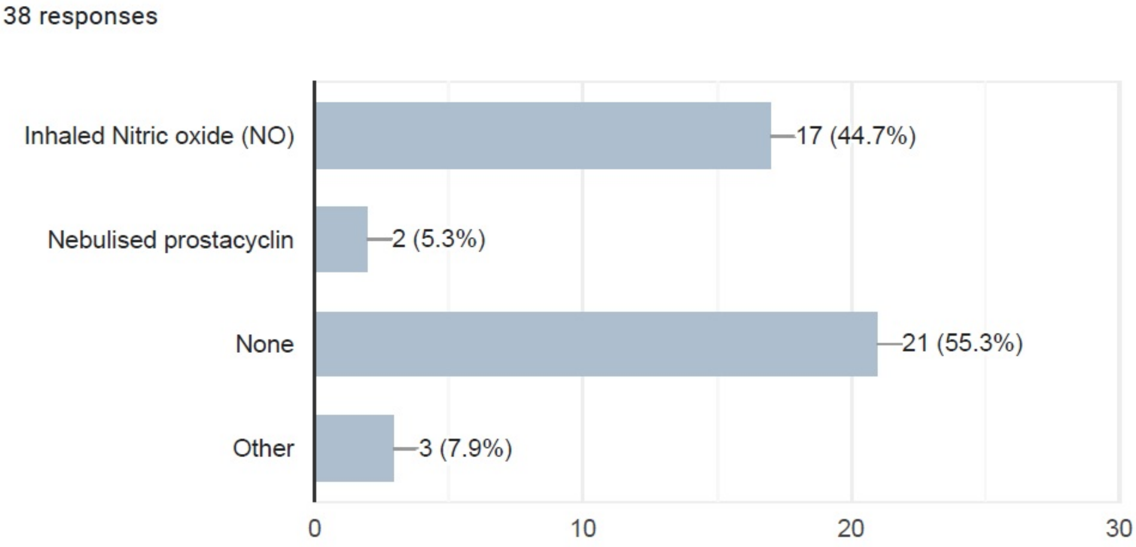

Consensus: None
Stability: Stable

6. Veno-Venous Extracorporeal membrane oxygenation (V-V ECMO) may be considered in C-ARF patients on invasive mechanical ventilation?

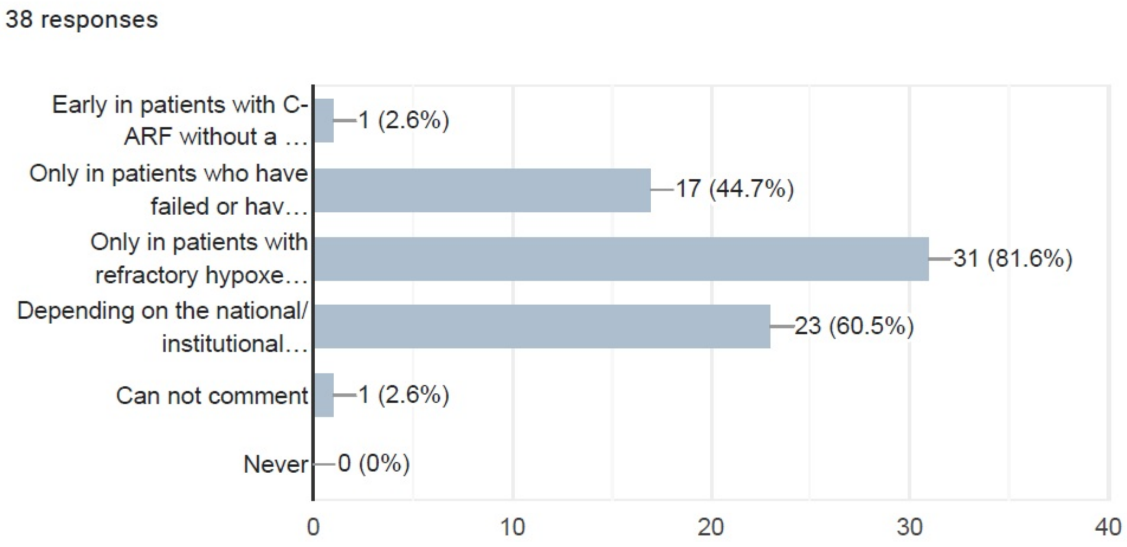

Consensus: Option 3 towards agreement
Stability: Stable

Section-4: Infection Control

1. The following are considered aerosol-generating procedures (AGPs)?

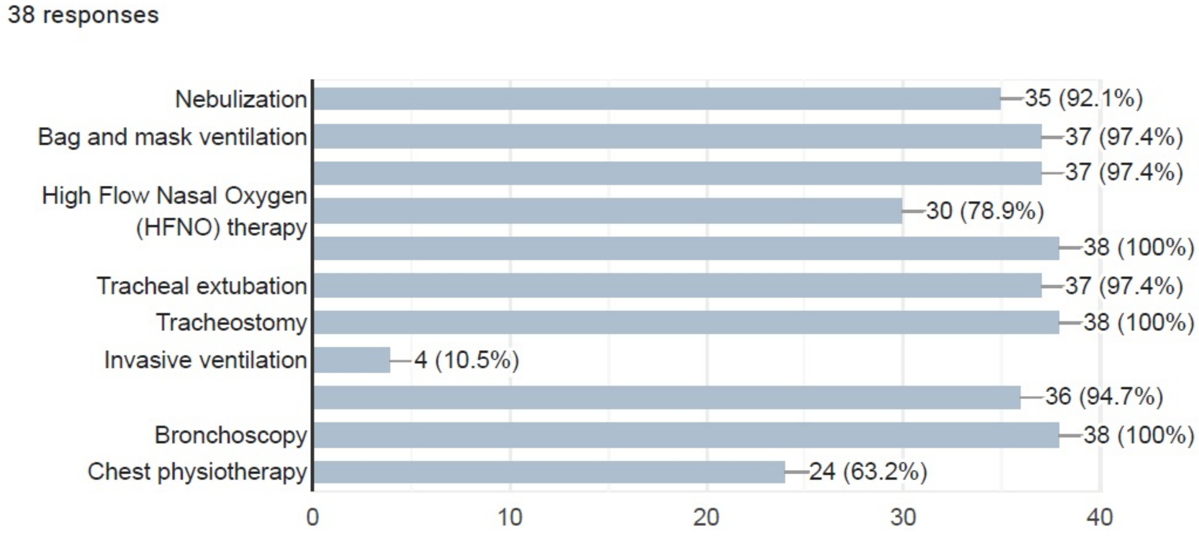

Consensus: Options 1, 2, 3, 5, 6, 7, 9 & 10 towards agreement
Stability: Stable

2. High flow nasal oxygen (HFNO) produces less aerosols as compared to non-invasive ventilation (NIV) with a face mask.

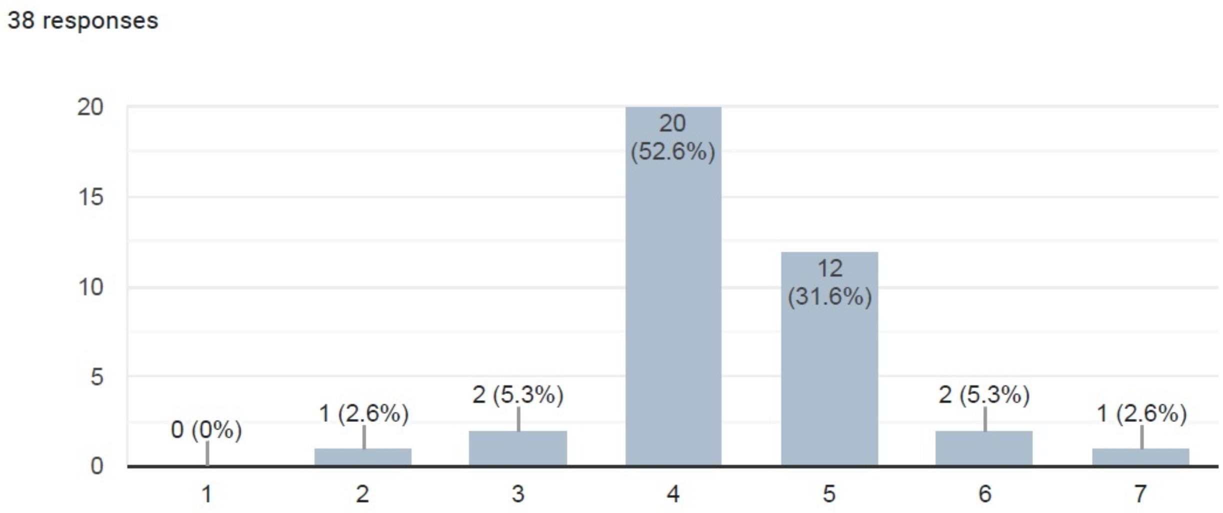

Consensus: None
Stability: Not stable

3. The following measures may be considered in the ICU to prevent cross-transmission of SARS-CoV-2?

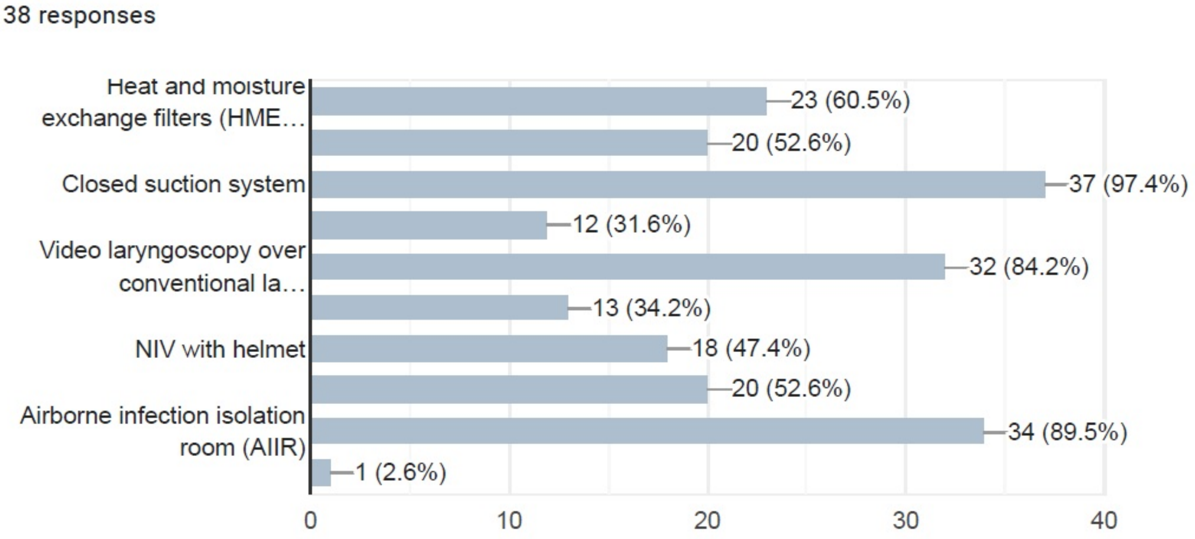

Consensus: Options 3,5 & 9 towards agreement
Stability: Stable

4. Which personal protective equipment (PPE) is acceptable for use during an AGP in ICU?

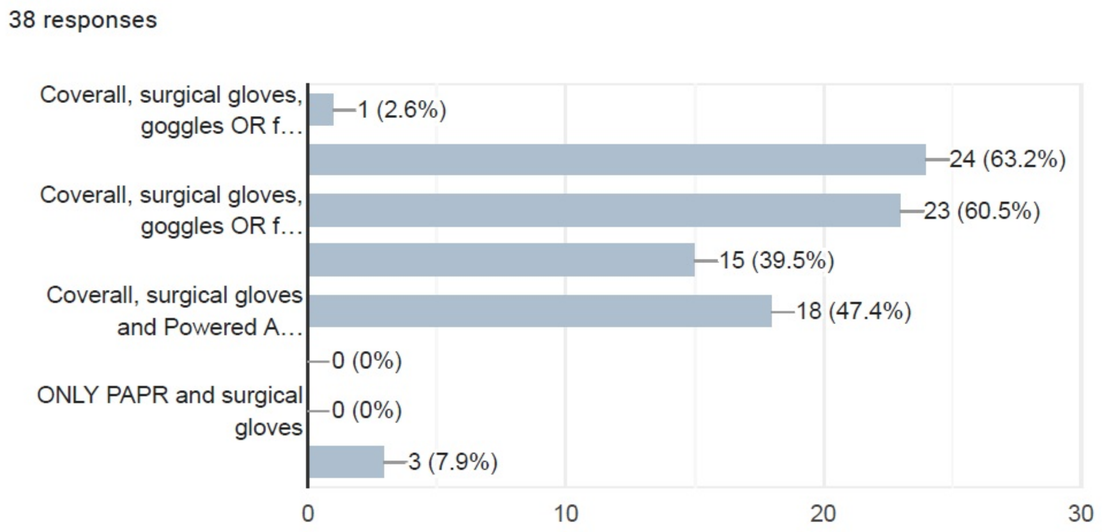

Consensus: None
Stability: Options were reshuffled in this round however, results seem stable.

Section-5: Weaning and Tracheostomy

1. Which weaning strategy would you prefer for liberation from invasive mechanical ventilation in patients with C-ARF?

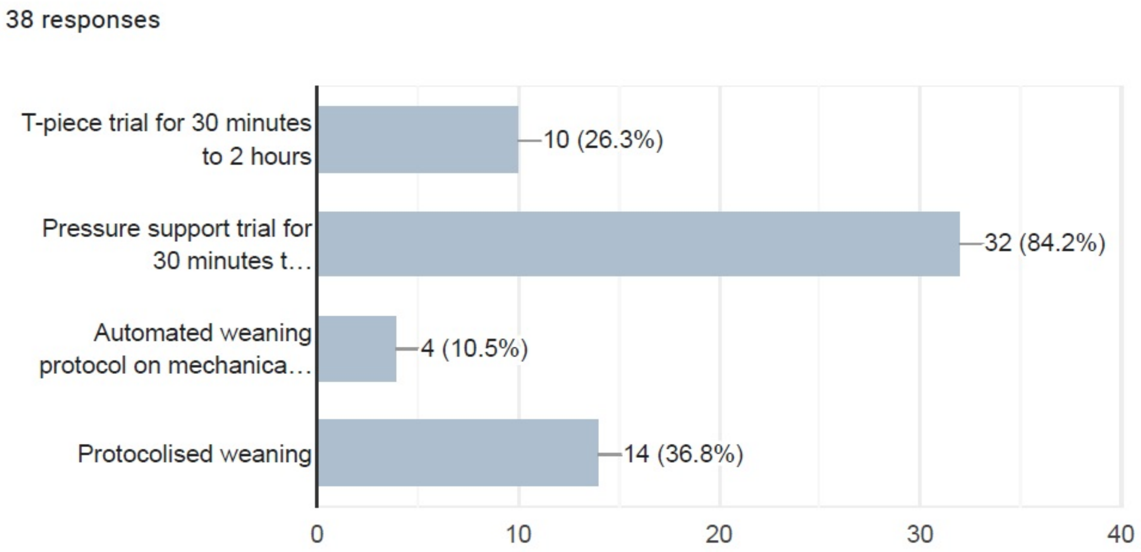

Consensus: Option 2 towards agreement
Stability: Comparison not available, as language of this question was changed in round three.

2. Chest physiotherapy could be beneficial in patients with C-ARF.

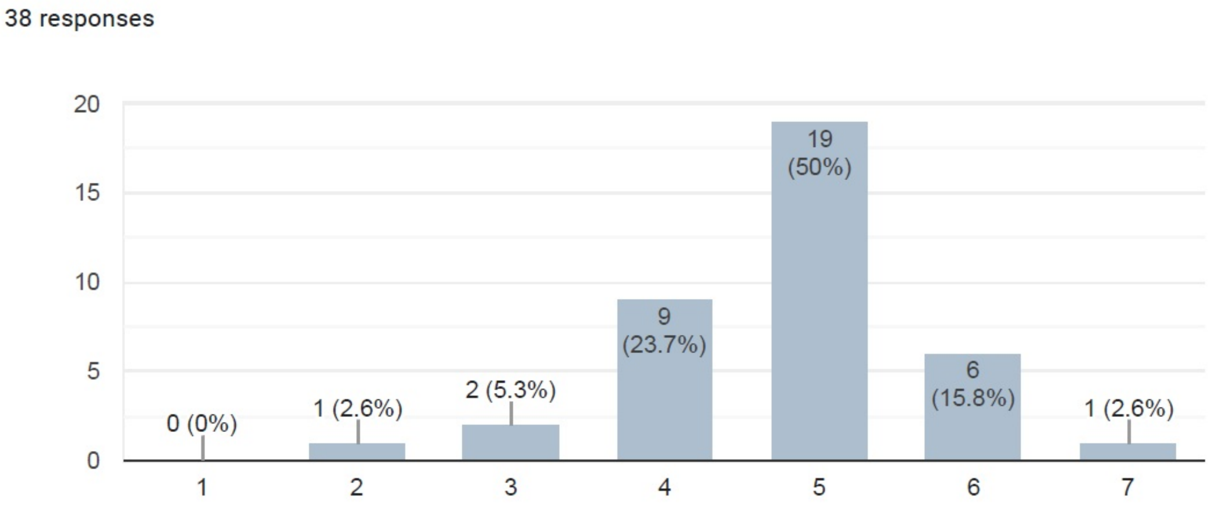

Consensus: None
Stability: Comparison not available, as language of this question was changed in round three.

3. Early mobilization of patients is BENEFICIAL in patients on respiratory support for C-ARF.

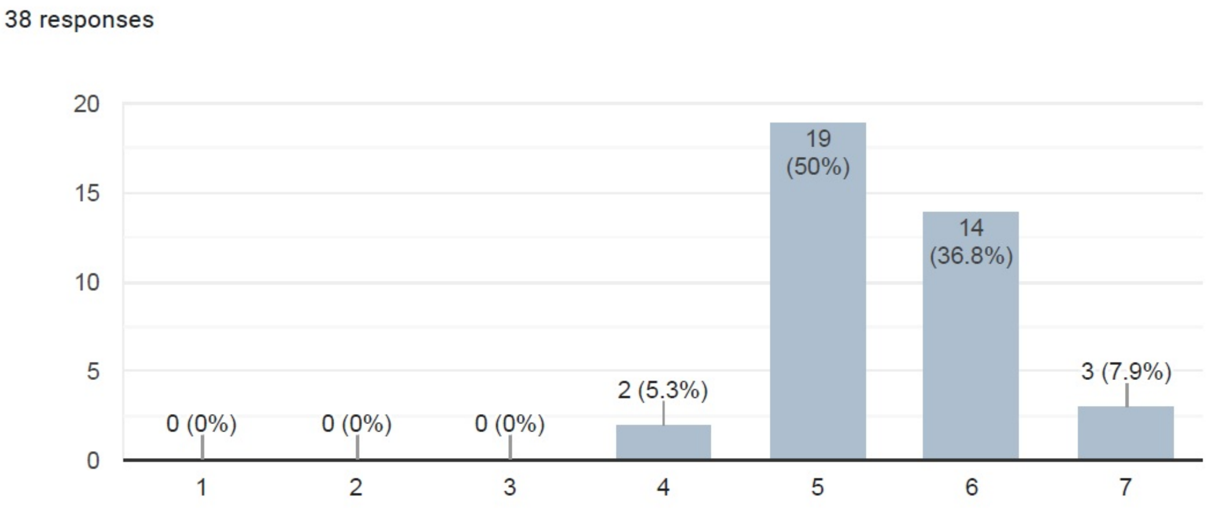

Consensus: Towards agreement
Stability: Stable

4. Delay in liberation from invasive mechanical ventilation has a lower risk of reintubation in patients with C-ARF.

38 responses

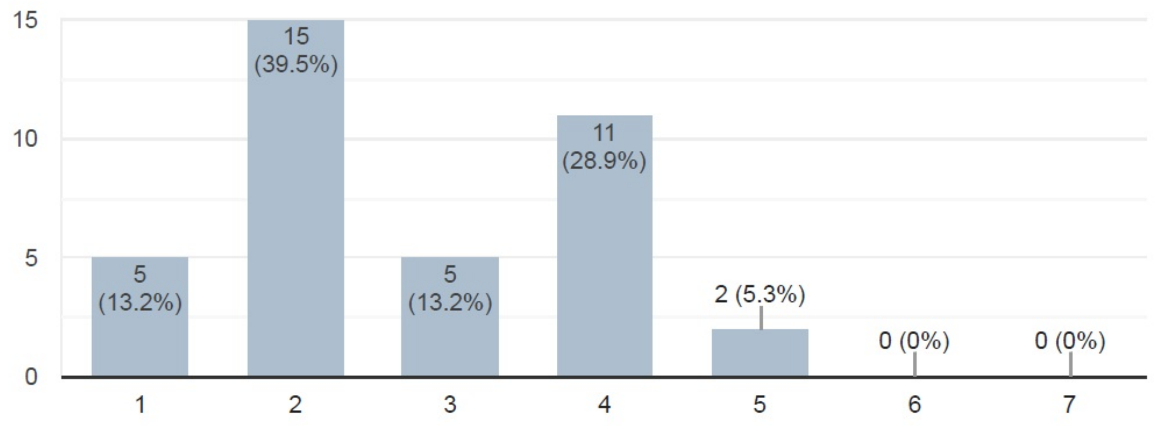

Consensus: None
Stability: Comparison not available, as language of this question was changed in round three.

5. When should tracheostomy be considered to facilitate weaning from invasive mechanical ventilation?

38 responses

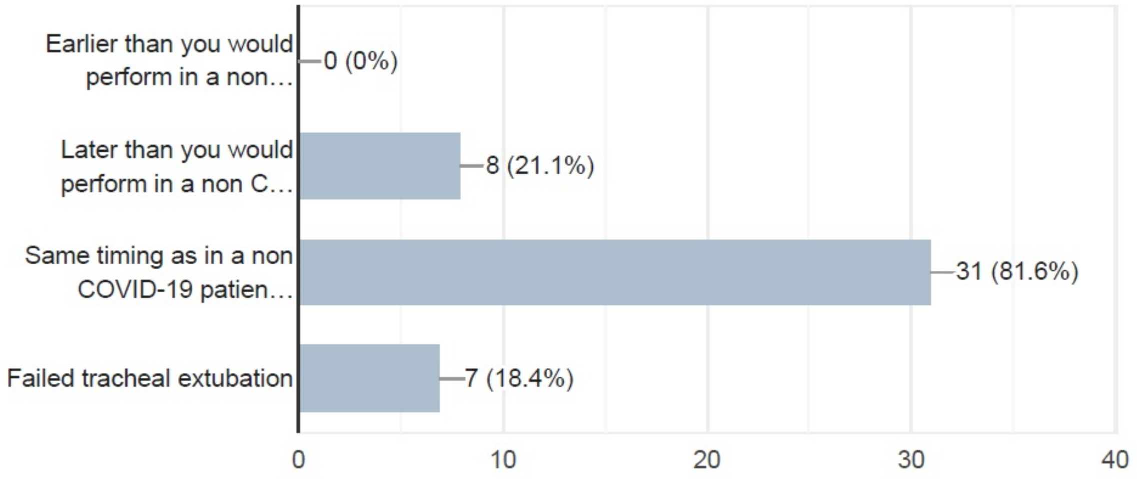

Consensus: Option 3 towards agreement
Stability: Comparison not available, as language of this question was changed in round three.

6. Which of the following technique of performing tracheostomy is preferred in patients with C-ARF?

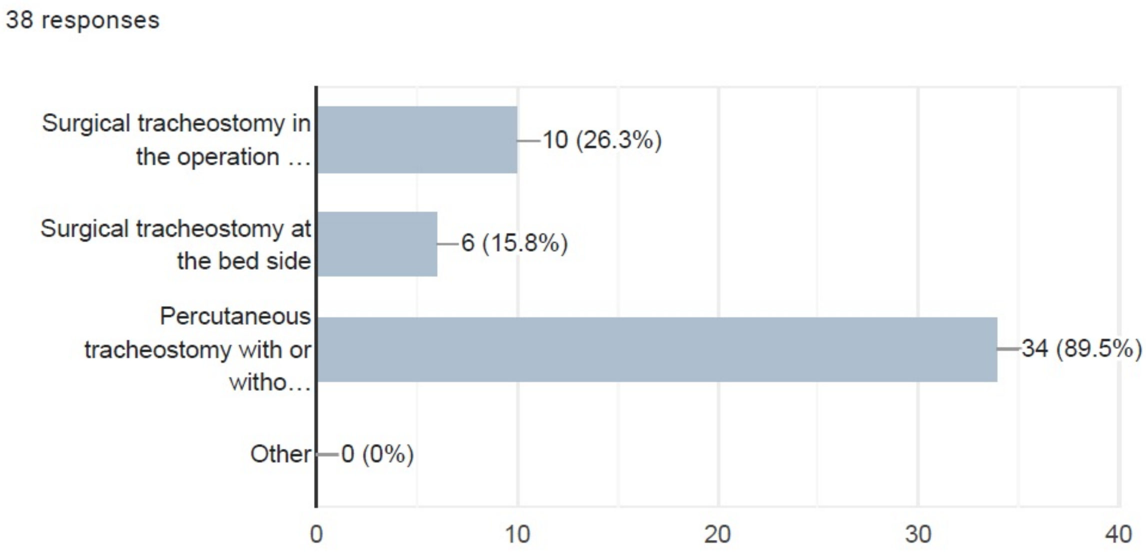

Consensus: Option 3 towards agreement
Stability: Comparison not available, as language of this question was changed in round three.
