# Supplementary material for: Expert consensus statements for the management of COVID-19-related acute respiratory failure using a Delphi method
Source: Crit Care. 2021 Mar 16;25:106. doi: 10.1186/s13054-021-03491-y (PMC7962430; doi:10.1186/s13054-021-03491-y)

Report of Survey Questionnaire Four

Section 1: Non-invasive respiratory interventions

1. The pathophysiology of COVID-19 related acute respiratory failure (C-ARF) is similar to that of acute respiratory distress syndrome (ARDS).

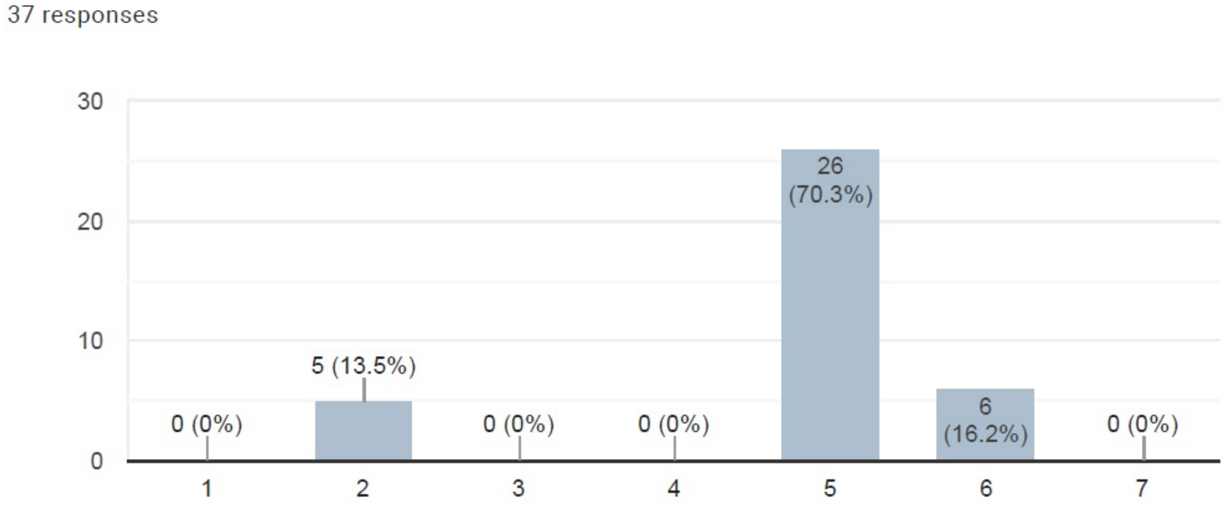

2. Based on your experience, HFNO may avoid the need for tracheal intubation and INVASIVE MECHANICAL VENTILATION in patients with C-ARF.

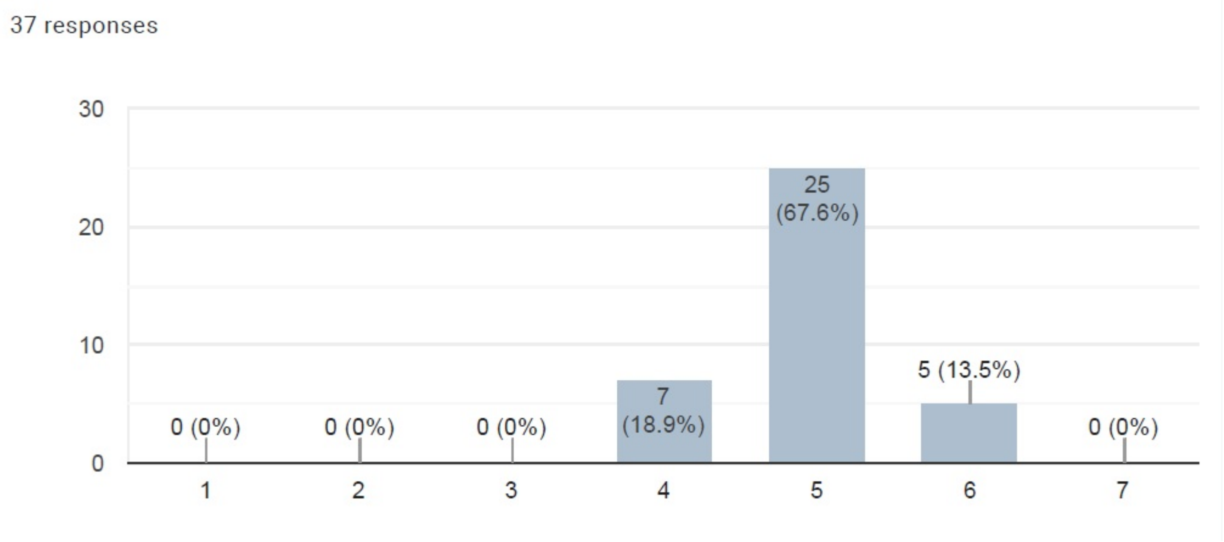

3. NIV may be considered in the following clinical scenarios in patients with C-ARF?

37 responses

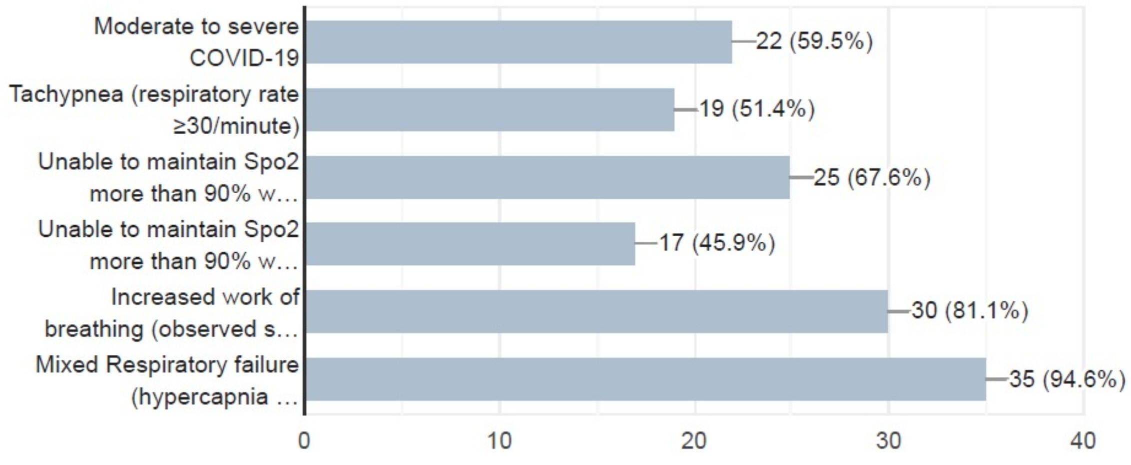

Section 2: Invasive Mechanical Ventilation

1. "Lung protective ventilation" should be used for patients with C-ARF on invasive mechanical ventilation.

37 responses

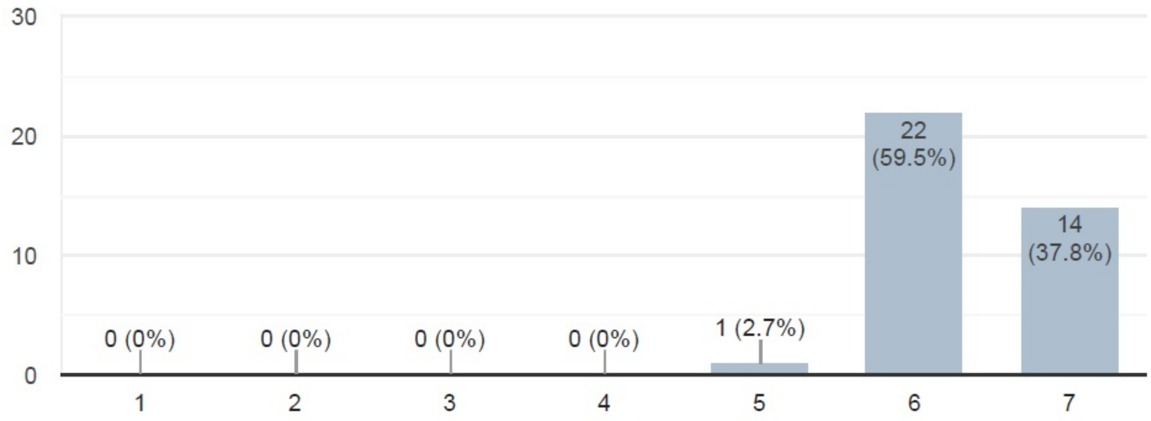

2. The invasive mechanical ventilation strategy in C-ARF should be targeted to the following?

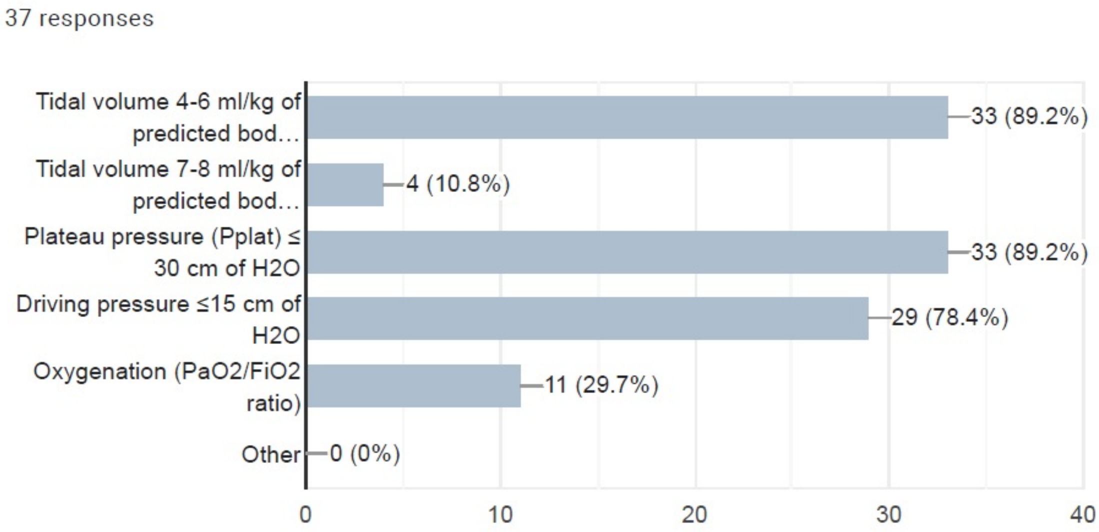

Section 3: Refractory Hypoxemia

1. Prone position during invasive mechanical ventilation of C-ARF is effective when done for (duration per session)?

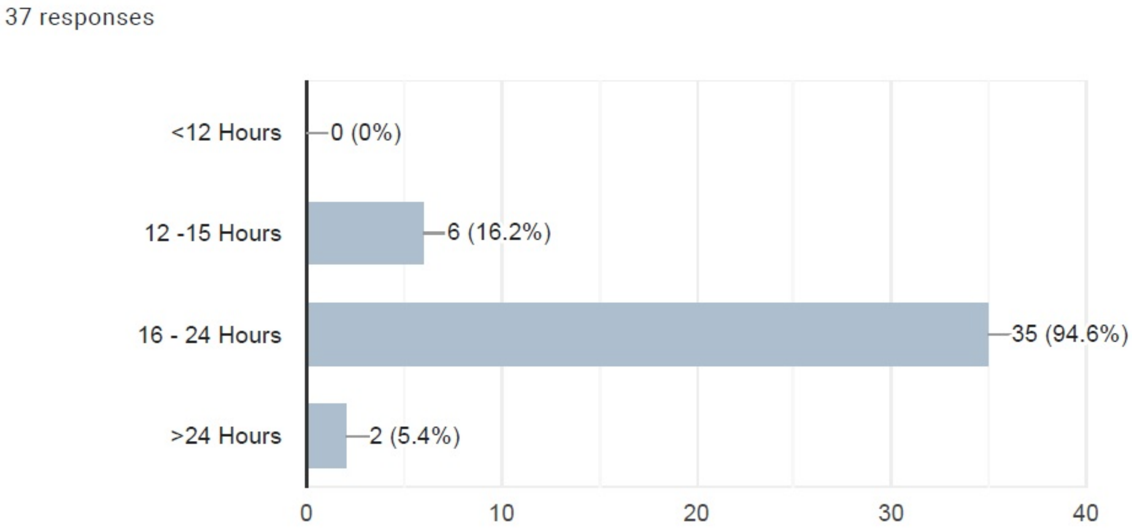

Section 5: Weaning and Tracheostomy

1. Which weaning strategy would you prefer for liberation from invasive mechanical ventilation in patients with C-ARF?

37 responses

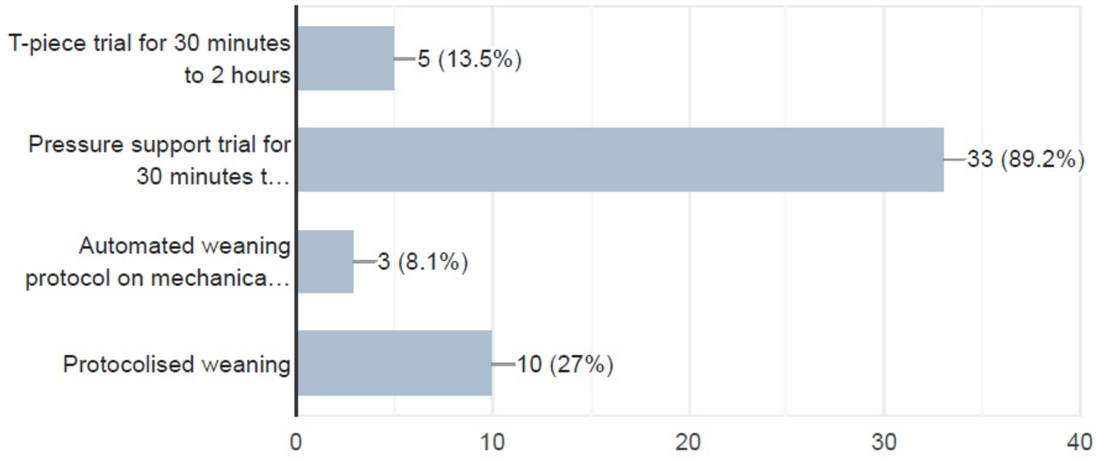

2. Chest physiotherapy could be beneficial in patients with C-ARF.

37 responses

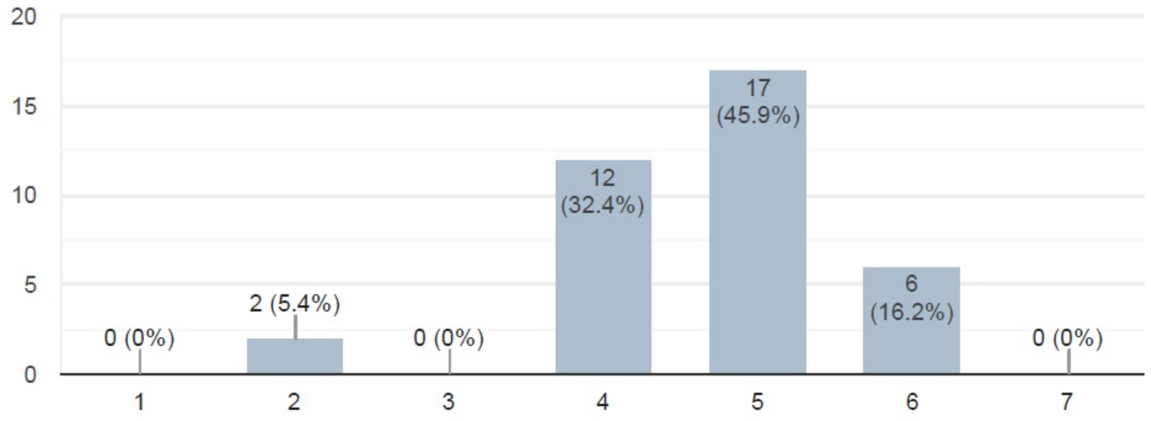

3. Delay in liberation from invasive mechanical ventilation has lower risk of reintubation in patients with C-ARF.

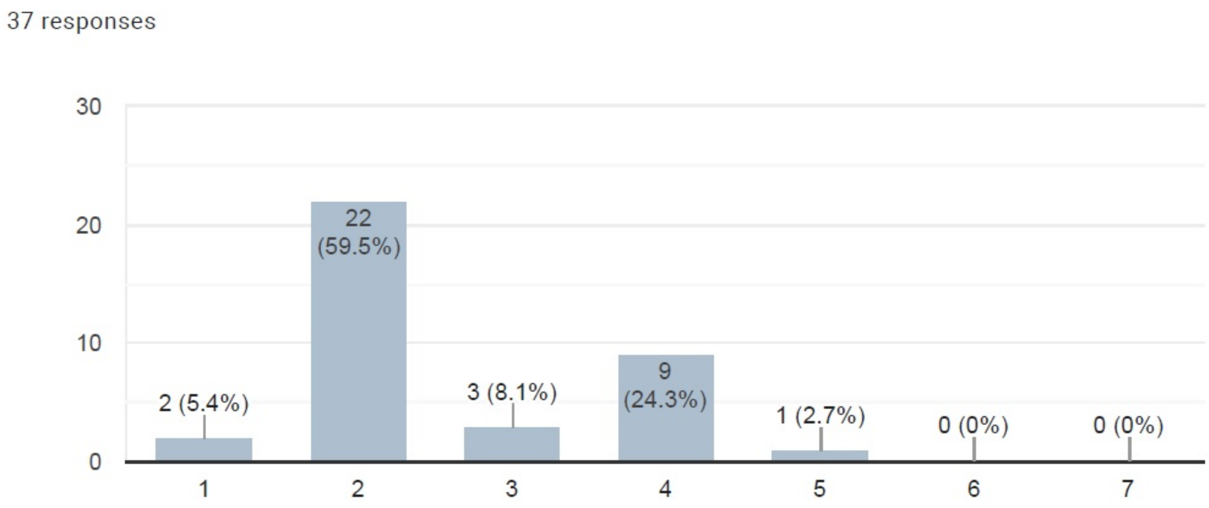

4. When should tracheostomy be considered to facilitate weaning from invasive mechanical ventilation?

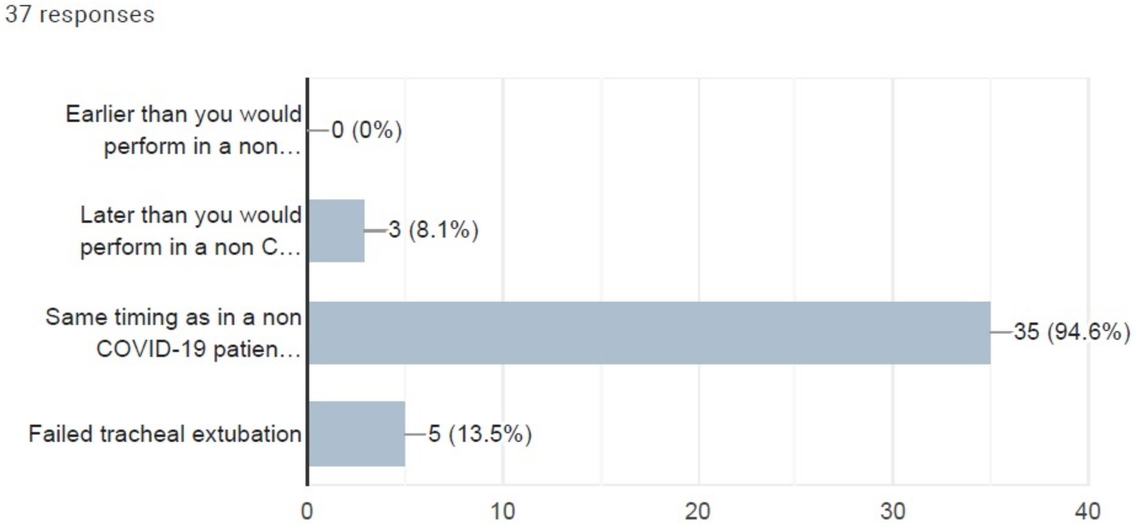

5. Which of the following technique of performing tracheostomy is preferred in patients with C-ARF?

37 responses

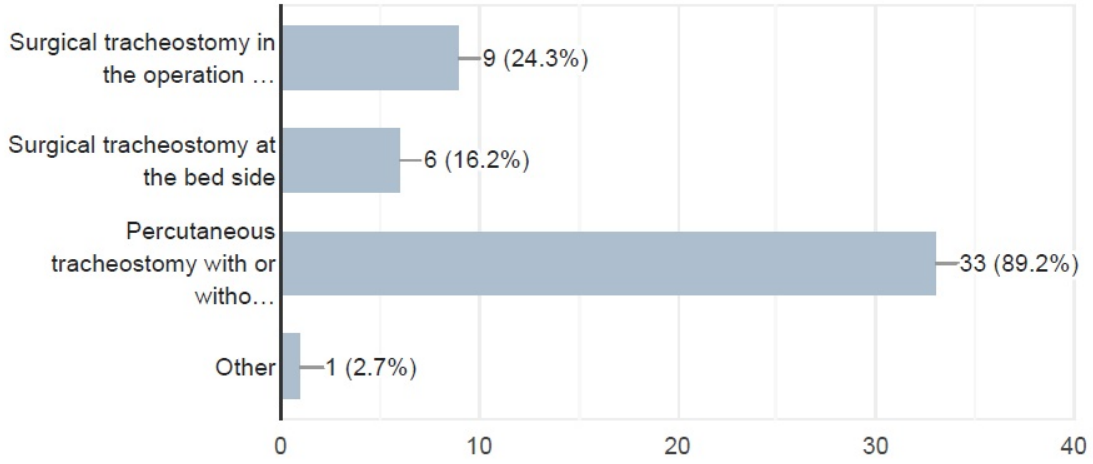

Supplement: Supplementary file 6 — Additional file 6. Survey Report 4. [file 13054_2021_3491_MOESM6_ESM.pdf]
